# Association Between Cardiac Natriuretic Peptides and Lipid Profile: a Systematic Review and Meta-Analysis

**DOI:** 10.1038/s41598-019-55680-z

**Published:** 2019-12-16

**Authors:** Francesco Spannella, Federico Giulietti, Marica Bordicchia, John C. Burnett, Riccardo Sarzani

**Affiliations:** 1 Internal Medicine and Geriatrics, IRCCS INRCA, Via della Montagnola 81, Ancona, Italy; 2 Department of Clinical and Molecular Sciences, University “Politecnica delle Marche”, Via Tronto 10/a, Ancona, Italy; 3 Cardiorenal Research Laboratory, Division of Cardiovascular Diseases, Mayo Clinic and Mayo Clinic College of Medicine, Rochester, Minnesota USA

**Keywords:** Diagnostic markers, Risk factors, Dyslipidaemias, Dyslipidaemias, Metabolic syndrome

## Abstract

Cardiac natriuretic peptides (NPs) play a fundamental role in maintaining cardiovascular (CV) and renal homeostasis. Moreover, they also affect glucose and lipid metabolism. We performed a systematic review and meta-analysis of studies investigating the association of NPs with serum lipid profile. A PubMed and Scopus search (2005–2018) revealed 48 studies reporting the association between NPs and components of lipid profile [total cholesterol (TC), low-density lipoprotein cholesterol (LDLc), high-density lipoprotein cholesterol (HDLc) and triglycerides (TG)]. Despite high inconsistency across studies, NPs levels were inversely associated with TC [k = 32; pooled r = −0.09; I^2^ = 90.26%], LDLc [k = 31; pooled r = −0.09; I^2^ = 82.38%] and TG [k = 46; pooled r = −0.11; I^2^ = 94.14%], while they were directly associated with HDLc [k = 41; pooled r = 0.06; I^2^ = 87.94%]. The relationship with LDLc, HDLc and TG lost significance if only studies on special populations (works including subjects with relevant acute or chronic conditions that could have significantly affected the circulating levels of NPs or lipid profile) or low-quality studies were taken into account. The present study highlights an association between higher NP levels and a favorable lipid profile. This confirms and extends our understanding of the metabolic properties of cardiac NPs and their potential in CV prevention.

## Introduction

The natriuretic peptide (NP) system is a family of cardiovascular (CV) hormones that includes A-type NPs, B-type NPs and C-type NPs. The first two peptides are collectively called “cardiac NPs”, because primarily produced in cardiomyocytes^1^ and act as hormones, while C-type NP is synthesized mostly by endothelium in several vascular beds^2^ and has an important role in bone growth. Cardiac NPs are released by the heart as a result of muscular wall stretch due to increased intraventricular volume and/or cardiac transmural pressure^3,4^, being well known useful biomarkers with key clinical implications. Cardiac NPs are synthesized as precursor proteins and cleaved into the N-terminal fragments (NT-proANP and NT-proBNP) and the active hormones (ANP and BNP) on release into the circulation^5^. Both A-type and B-type NPs play a fundamental role in maintaining CV and renal homeostasis, by regulating arterial blood pressure (BP), blood volume and sodium balance^4,5^. More recently, cardiac NPs were found to have a physiological role in glucose and lipid metabolism in adipose and muscle tissues, by stimulating lipolysis, increasing energy expenditure, enhancing lipid oxidation, browning of white adipocytes and decreasing inflammatory cytokines and insulin resistance^6,7^. Insulin-induced glucose entry favors triglycerides accumulation in adipocytes by shutting down NP-induced lipolysis through a sharp increase in the expression of the clearance receptor for NPs^7^. It is therefore understandable why these hormones are actually considered very relevant in the assessment of cardiometabolic pathophysiology and clinical risk^8,9^. Furthermore, recent evidences suggest that cardiac NPs could be able to directly affect circulating cholesterol levels by reducing the expression of proprotein convertase subtilisin/kexin type 9 (PCSK9) in adipocytes^10^. Guided by their multiple actions on lipid metabolism, several clinical studies in the past decade showed the associations between circulating NPs levels, metabolic syndrome (MetS) and serum lipid profile. Higher NPs levels are likely associated with a cardiometabolic protection with less MetS and a favorable profile of its components [i.e. lower insulin resistance, lower blood pressure, higher levels of high-density lipoprotein cholesterol (HDLc)]^11,12^. However, the several studies that evaluated the relationship between circulating levels of cardiac NPs and the main components of serum lipid profile have led to mixed results. In some studies, the association appears to be restricted only to certain components of the lipid profile, with a discrepancy between one study and another. Moreover, there is a huge heterogeneity regarding the studied population (in reference to age, NPs levels, CV and no-CV clinical settings), the NP analyzed and the NPs assay used. Many studies took into account only some lipid components, for example those related to MetS. Lastly, many studies have a small sample size, which limits their strength of scientific evidence. On this basis, we performed a systematic review and meta-analysis of studies that evaluated the association between circulating cardiac NPs (A-type NPs and B-type NPs) levels and serum lipid profile, to comprehensively assess how the circulating levels of these hormones associate with serum total cholesterol (TC), low-density lipoprotein cholesterol (LDLc), HDLc and triglycerides (TG).

## Methods

This report adheres to the Meta-analysis Of Observational Studies in Epidemiology (MOOSE) guidelines^13^.

### Eligibility criteria and search strategy

Studies were eligible for inclusion if they were observational studies (prospective or retrospective cohort, case-control or cross-sectional studies) or clinical trials investigating the association between cardiac NPs and components of lipid profile (TC, LDLc, HDLc and TG). Medline (PubMed) and Scopus were searched. The main search was run on 11^th^ Dec 2018 and updated weekly until May 2019. The keywords regarding cardiac NPs and lipid profile were typed in various combinations using boolean operators (see the detailed search strategy in Supplemental Methods). Hand searches of reference lists of articles and relevant literature reviews were used to complement the computer search. The search was limited to English language studies published in peer-reviewed journals. No limits in the sample size were taken into account. We excluded studies focused on patients aged <18 years.

### Study selection and data extraction

Two independent investigators (F.S., F.G.) screened all identified records (title and abstract) and assessed the selected full-text articles for eligibility. Disagreements were resolved through discussion. Descriptive, methodological and outcome data were extracted from all the eligible studies by the two reviewers who worked independently using a predefined data extraction form. The following data were collected: study design, number of enrolled subjects, mean age, sex, body mass index (BMI), renal function [estimated glomerular filtration rate (eGFR)], prevalence of diabetes and hypertension, prevalence of lipid-lowering treatment, NPs levels, circulating lipid levels (TC, LDLc, HDLc, TG), NPs assay. In studies reporting median values, the mean and variance were estimated from the median, range, and the sample size, according to Wan X *et al*.^14^.

In the manuscript, we included both “NT-proBNP” and “BNP” with the term “B-type NPs”, and, similarly, we included “NT-proANP”, “MR-proANP” and “ANP” with the term “A-type NPs”.

### Assessment of risk of bias and study quality

The Newcastle-Ottawa Scale (NOS) for assessing the quality of nonrandomized studies in meta-analyses^15^ was adapted for use in the current review, as previously reported^16^. The studies was assessed in 3 domains: (1) the selection (representativeness of the sample population, appropriate sample size, and ascertainment of the exposure; (2) the comparability (appropriate control of confounding factors); and (3) the outcome (appropriate assessment of the outcome and appropriate description of the statistical test). From the original quality assessment tool, the item “respondents comparability and response rate” was excluded because not applicable to the current review. Instead, the item “appropriate sample size” was added in the section “selection” (a sample size of 100 or more was justified as satisfactory). The maximum attainable quality score was 6. A score of 5 was chosen as cutoff to indicate studies of high quality (Supplemental Table 1).

### Statistical analysis

Four distinct meta-analyses were performed for each individual component of the lipid profile (TC, LDLc, HDLc, TG). Correlation coefficient (r) has been chosen as the effect estimate for the data synthesis. As the majority of studies reported univariate analyses for the outcome of interest, Pearson or Spearman correlations were extracted. Adjusted beta was extracted if available in the absence of the univariate analyses. If the authors reported different effect estimates (i.e. one-way analysis of variance), data were extracted and used for the reconstruction of effect size (ES) and data synthesis. In studies reporting the outcome of interest in subgroups of the study population, we analyzed each subgroup separately in order to consider the ES of each one. Data were synthesized using meta-analytic methods^17^, and statistically pooled by the standard meta-analysis approach, i.e. studies were weighted by the inverse of the sampling variance. The DerSimonian and Laird random effects model was used as a conservative approach to account for different sources of variation among studies. Forest plots were constructed to graphically represent the results. Q statistics were used to assess heterogeneity among studies. A significant Q value indicates a lack of homogeneity of findings among studies^17^. I^2^ statistics were then used to quantify the proportion of observed inconsistency across study results not explained by chance^18^. I^2^ values of <25%, 50% and >75% represent low, moderate and high inconsistency, respectively^18^. Several variables were identified and their effects on outcome examined. Sensitivity analyses were performed by excluding studies with possible confounders in order to assess the influence of confounders on the pooled ES. Categorical variables were treated as moderators and the ES was assessed and compared across subgroups formed by these moderators (study design, age classes, BMI classes, cardiac NP type, special populations, NT-proBNP assays, BNP assays, study quality). According to each study sample, we defined “studies on special populations” those works including subjects with relevant acute or chronic conditions that could have significantly affected the circulating levels of NPs or lipid profile, and therefore their association: patients with human immunodeficiency virus, kidney transplant patients, women with polycystic ovary syndrome, patients in peritoneal dialysis, subjects with active systemic lupus erythematosus, patients with acute myocardial infarction, patients with acute ischemic stroke, patients hospitalized for worsening heart failure. NP assay was considered as moderator for B-type NPs, while the low number of included studies (k = 5) and the different assays used did not allow us to perform this sensitivity analysis for A-type NPs. Continuous variables were examined as covariates using random effects meta-regression (age, BMI, NT-proBNP levels, prevalence of males, eGFR, prevalence of diabetics, prevalence of hypertensives, prevalence of lipid-lowering therapy). Sub-group analyses were performed to assess the effect of study quality (NOS score, risk of bias) on the calculated estimates. Given the possible important bias linked to the lipid-lowering treatment, we also evaluated the overall ES after considering only the included studies explicitly conducted on untreated subjects (subjects without lipid-lowering therapy). The presence of publication bias was investigated through funnel plots both visually and formally by trim and fill analysis and Eggers’s linear regression method^19^. A *p* value less than 0.05 was used to indicate statistical significance. All analyses were conducted using a computer software package (ProMeta Version 2, Italy).

## Results

### Included studies

**Figure 1:**
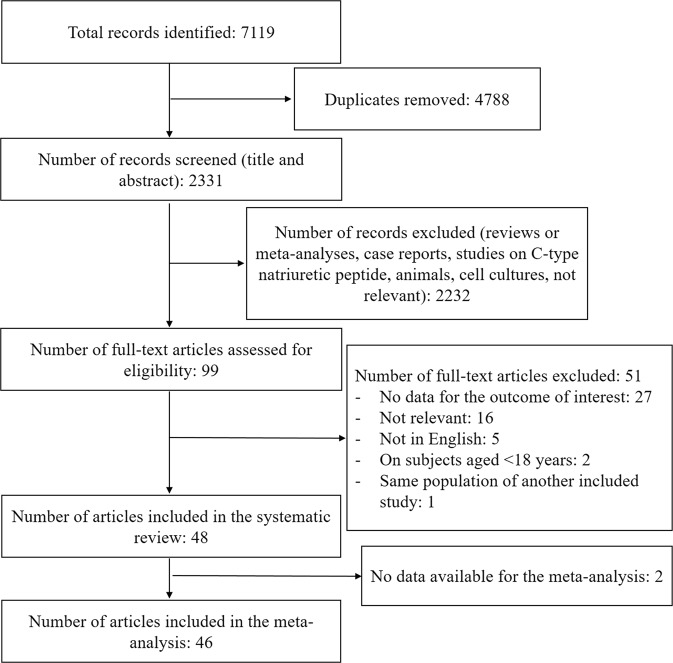
Flow-chart showing the study selection process.

The study selection process is described in Fig. 1. Among the initial 7119 records, 48 studies published between 2005 and 2018 met our inclusion criteria and 46 were included in the meta-analysis^20–65^. The characteristics of the 46 included studies are described in Supplemental Table 2. Of the 46 resulting studies, the majority was cross-sectional (n° 28), 16 were cohort and 2 were case-control studies. Ten studies reported the outcome of interest in subgroups of study population, therefore the ES was considered accordingly. The majority of studies (n° 27) were focused on N-terminal pro B-type natriuretic peptide (NT-proBNP), 14 on BNP and only 5 on A-type NPs. The mean of cardiac NPs levels varied substantially across the available studies. In the sensitivity analyses, 13 studies included special populations^21,31,35,36,38,39,41,43,46,49,56,60,63^. Regarding lipid-lowering treatment, 23 studies reported the prevalence of treated subjects, and only 11 studies were conducted on untreated subjects^20,21,32,36,42,43,52,54,55,64,66^. Thirty-seven studies reported the assays for B-type NPs. All the assays had a good coefficient of variations (<10%), except for one study, although it reported a coefficient of variation <15%^32^. Regarding NT-proBNP, 22 studies used electrochemiluminescence immunoassay (ECLIA)^20,22–25,27–29,33,37,38,40,41,45,48,51–55,57,61^, 2 studies used enzyme immunoassay (EIA)^60,64^, 1 study used immunofluorescence assay (IFA)^39^ and 1 study used chemiluminescence immunoassay (CLIA)^46^. Regarding BNP, 7 studies used assays for NH_2_-terminal fragment^26,32,43,49,50,63,66^, 3 studies used assays for COOH-terminal fragment^42,44,62^ and 1 study used radioimmunoassay (RIA)^21^. In the systematic review, we found two studies that have not been included in the meta-analysis due to the lack of usable data. One article investigated the link between NT-proBNP and lipids based on metabolomics profile determined by ^1^H-NMR spectroscopy in 872 subjects^67^. In this study, higher NT-proBNP levels were linearly associated with a beneficial lipoprotein profile, including lower very low-density lipoprotein (VLDL), intermediate-density lipoprotein (IDL), and LDL-particles along with higher large HDL particle measures, lower small dense HDL particle measures and lower TG. Instead, the other study showed a negative correlation between LDLc and NT-proBNP/BNP ratio on 195 in-patients with acute heart failure (Rho = −0.36, p < 0.01)^68^.

### Associations between cardiac NPs and lipid profile

**Figure 2:**
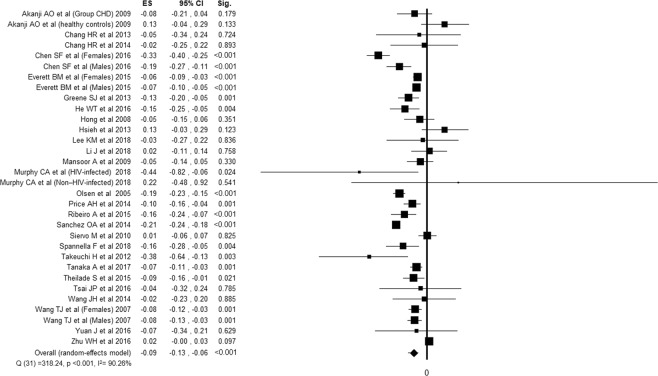
Forest plot showing individual and overall ES of studies that evaluated the association between cardiac NPs and TC (k = 32). The size of the boxes is inversely proportional to the size of the result study variance, so that more precise studies have larger boxes. The ES is expressed as correlation coefficient (r) and the correspondent 95% confidence interval (CI). ES = effect size; CI = confidence interval; Sig. = p-value.

**Figure 3:**
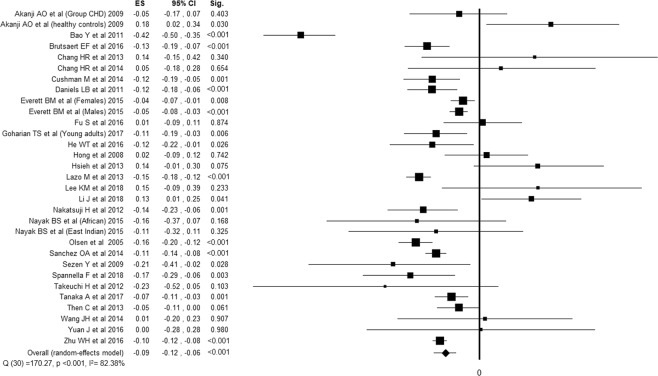
Forest plot showing individual and overall ES of studies that evaluated the association between cardiac NPs and LDLc (k = 31). The size of the boxes is inversely proportional to the size of the result study variance, so that more precise studies have larger boxes. The ES is expressed as correlation coefficient (r) and the correspondent 95% confidence interval (CI). ES = effect size; CI = confidence interval; Sig. = p-value.

**Figure 4:**
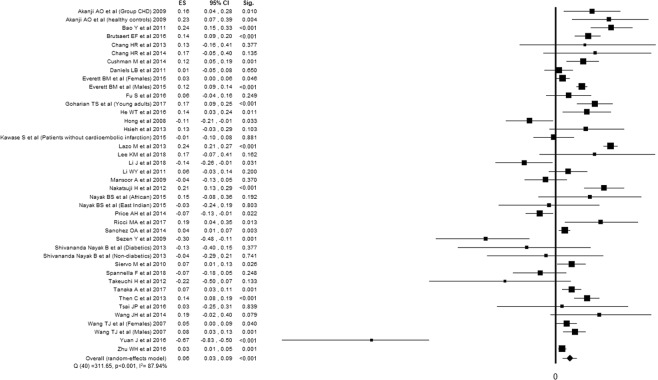
Forest plot showing individual and overall ES of studies that evaluated the association between cardiac NPs and HDLc (k = 41). The size of the boxes is inversely proportional to the size of the result study variance, so that more precise studies have larger boxes. The ES is expressed as correlation coefficient (r) and the correspondent 95% confidence interval (CI). ES = effect size; CI = confidence interval; Sig. = p-value.

**Figure 5:**
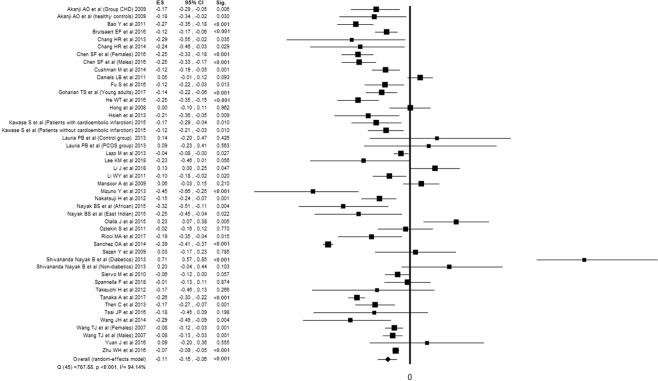
Forest plot showing individual and overall ES of studies that evaluated the association between cardiac NPs and triglycerides (k = 46). The size of the boxes is inversely proportional to the size of the result study variance, so that more precise studies have larger boxes. The ES is expressed as correlation coefficient (r) and the correspondent 95% confidence interval (CI). ES = effect size; CI = confidence interval; Sig. = p-value.

Twenty-seven studies^20,21,24–26,31–34,38,39,41,43,48,49,51,54–58,60–64,66^ reported the association between cardiac NPs and TC, for a total of 43428 subjects evaluated. Twenty-eight studies^20–25,27–30,32–34,37–39,44,45,51,52,55–57,59,61,63,64,66^ reported the association between cardiac NPs and LDLc, for a total of 44829 subjects evaluated. Thirty-six studies^20,21,24–26,31–34,38,39,41,43,48,49,51,54–58,60–64,66^ reported the association between cardiac NPs and HDLc, for a total of 49951 subjects evaluated. Thirty-nine studies^20–30,32–42,44–47,50–57,59–64^ reported the association between cardiac NPs and TG, for a total of 39649 subjects evaluated. All the components of lipid profile was associated with NPs. Despite high inconsistency between studies, TC, LDLc and TG were inversely associated with NPs, while HDLc showed a positive association (Figs. 2–5).

### Sensitivity analyses

In order to search for possible sources of heterogeneity related to different characteristics of studies and populations, the moderator analysis was performed (Supplemental Tables 3–6). Regarding TC, studies on subjects with a mean age ≥65 years or a mean BMI <25 kg/m^2^ showed a stronger association. Moreover, the association was confirmed only in those studies that used ECLIA for NT-proBNP and was stronger in those studies that used assay for NH_2_-terminal fragment of BNP.

The associations with LDLc, HDLc and TG lost significance in studies on special populations and with low quality for LDLc and HDLc. Moreover, the association with LDLc was not confirmed for the studies that used RIA for NT-proBNP. Regarding HDLc, the positive association with NPs was stronger with A-type NPs compared to B-type NPs, while no difference between the two cardiac NP types emerged for the other lipids. Regarding TG, the negative association was confirmed only for those studies that used ECLIA and EIA for NT-proBNP, while the studies that used assay for NH_2_-terminal fragment of BNP showed the strongest association. No other analyzed moderators affected the association between NPs and lipids.

After considering only the included studies conducted on untreated subjects (subjects without lipid-lowering therapy), the associations with NPs confirmed their statistical significance for TC [k = 10; N = 26454; pooled r = −0.07 (95% IC −0.13–−0.01); *p* = 0.014; I^2^ = 91.87], HDLc [k = 8; N = 24452; pooled r = 0.05 (95% IC 0.00–0.10); *p* = 0.039; I^2^ = 87.71], LDLc [k = 8; N = 25522; pooled r = −0.09 (95% IC −0.13–−0.05); *p* < 0.001; I^2^ = 84.15] and TG [k = 9; N = 13514; pooled r = −0.10 (95% IC −0.17–−0.03); *p* = 0.004; I^2^ = 71.83].

In the meta-regression analyses for TC (Supplemental Fig. 1), the absolute value of the ES increased with the increase of age (k = 28; Beta = −0.004609; *p* = 0.005), with the decrease of BMI (k = 22; Beta = 0.02; *p* = 0.040) and with the increase of NT-proBNP levels (k = 13; Beta = −0.000023; *p* = 0.011), while sex, eGFR, prevalence of diabetics, prevalence of hypertensives, prevalence of lipid-lowering therapy had no significant effect on the observed ES. In the meta-regression analyses for LDLc, HDLc and TG (Supplemental Figs. 2–4), all the covariates analyzed (age, BMI, NT-proBNP levels, sex, eGFR, prevalence of diabetics, prevalence of hypertensives, prevalence of lipid-lowering therapy) had no significant effect on the observed ES.

### Publication bias

The funnel plots for TC, HDLc and TG did not show any publication bias (Egger’s linear regression test for TC: *p* = 0.389; for HDLc: *p* = 0.572; for TG: *p* = 0.349), as shown in Supplemental Figs. 5,7,8.

Regarding the association between NPs and LDLc, the funnel plot showed asymmetry with a slightly lower estimated ES [estimated ES = −0.10 (−0.13–−0.07), *p* < 0.001; number of trimmed studies: 4], although the Egger’s linear regression test was not significant (*p* = 0.588), as shown in Supplemental Fig. 6.

## Discussion

**Figure 6:**
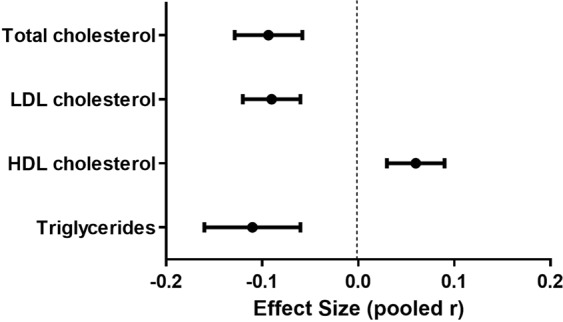
Associations between cardiac natriuretic peptides and lipid profile (Correlation coefficients).

The present systematic review and meta-analysis of 46 studies shows a continuous linear association between cardiac NPs and serum lipid parameters. Higher NPs levels are associated with a more favorable lipid profile, in particular with lower LDLc and TG, higher HDLc, as summarized in Fig. 6. These findings confirm a positive role of this hormonal system on circulating lipid levels and therefore on CV risk.

Dyslipidemia is one of the most common CV risk factors, and a major determinant of CV disease and mortality^69,70^. In addition to the well-established role of LDLc in determining atherosclerosis, all the other components of the lipid profile are also implicated in vascular damage^71^. Our meta-analysis highlights the association between cardiac NPs levels and all the components of the lipid profile.

The first clinical study on this topic was performed by Olsen and colleagues^20^ in 2005. They found that patients with dyslipidemia had lower NT-proBNP levels that were inversely associated with higher levels of both TC and TG. Afterwards, many other studies investigated the relationship between cardiac NPs and lipid profile, particularly in the context of the MetS^20,22,24,25,30,45,47,60,62,64^. Indeed, a close association between NPs system and MetS is well recognized^72^. Previous clinical studies found an association between higher NPs levels and less development of diabetes mellitus and MetS^11^. At the same time, hyperinsulinemia, a classical marker of MetS, induces an overexpression of the NPs clearance receptor (NPRC) in human adipocytes, contributing to lower NPs circulating levels in these subjects^7^. A genetic variant of the NPs precursor A gene (NPPA) is associated with higher circulating levels of both A-type and B-type NPs, and with higher HDLc levels and lower prevalence of obesity and MetS^12,73,74^. In our meta-analysis, no differences emerged between A-type and B-type NPs in the associations with lipid parameters (see moderator analysis in the Supplemental Material). However, HDLc was more strongly associated with A-type NPs than B-type NPs, although far fewer clinical studies on A-type NPs were available in literature (5 studies for A-type NPs vs 41 studies for B-type NPs). Interestingly, this is in line with the previous evidence that A-type NPs could exert more a cardiometabolic function compared to B-type NPs, which may be more related to cardiac remodeling^8^.

Several mechanisms that could explain the clinical relationship between circulating NPs and serum lipid levels are emerging. Cardiac NPs have the ability to positively affect distant target organs. In fact, NP receptors are expressed in several organs and tissues, such as vessels, kidney, skeletal muscle and adipose tissue^5^. In human adrenocortical cells, BNP was found to inhibit cholesterol biosynthesis stimulated by angiotensin II^75^. Adipose tissue and the kidney are the organs where NP receptors are mainly expressed^76^. Indeed, cardiac NPs exert several actions on adipocytes and lipid metabolism. They promote the “browning” of white adipocytes^77^ increasing the uptake of triglycerides derived from plasma triglyceride-rich lipoproteins. Brown adipose tissue is also actively involved in the metabolic flux of HDLc to the liver^78^. Furthermore, NPs promote the degradation of TG and the oxidation of the circulating non-esterified fatty acids, inducing mitochondrial biogenesis and thermogenesis^77,79^, hence the inverse association between NP and TG found in our work. Recently, based on a metabolomics approach, NT-proBNP had an inverse association with aromatic and branched-chain aminoacids and related degradation intermediates, likely due to the induction of mitochondrial biogenesis by NP^67^. Most of these metabolic actions are mediated by the binding of cardiac NPs to the NP receptor A (NPRA) that induces the generation of the second messenger cyclic guanosine monophosphate (cGMP)^80^. Overexpressing BNP or genetic/pharmacological cGMP augmentation in several animal models induced adipose tissue browning and lipid oxidation, promoted mitochondrial biogenesis and fat oxidation in skeletal muscle, preventing obesity and glucose intolerance^77,79,81^. Finally, cardiac NPs were recently found to exert a direct role on the main determinants of LDLc levels, such as LDLc receptor (LDLR) and PCSK9^10^. PCSK9 is expressed in human adipose tissue and regulated by insulin and ANP, which act in opposite ways. In fact, ANP is able to reduce the insulin-mediated induction of PCSK9, thereby reducing the degradation of LDLR^10^. All these mechanisms, together with the well-established protective role against MetS and diabetes mellitus, can lead to a more favorable circulating lipid profile.

Our systematic review and meta-analysis focused on cardiac NPs (A-type NPs and B-type NPs) given the extensive literature regarding their relationship with CV risk factors, including MetS and lipid metabolism, based on their well-known endocrine effects. Although not taken into account in our work, recent evidence is emerging on the CV and metabolic role of C-type NP. Very recently, C-type NP has been identified as a good early marker of renal damage in diabetic patients, probably reflecting renal inflammation and fibrosis^82^. Moreover, an important role on blood pressure, through its anti-fibrotic and vasodilatory effect^83^ and on lipid metabolism has been found in transgenic mice, in which the overexpression of C-type-NP led to increase in fatty acid β-oxidation and lipolysis and decrease in insulin-resistance and fat weight^84,85^.

Several moderators were taken into account in our meta-analysis. Sex did not affect our outcome, likely due to the average age of most populations studied with most menopausal women, as well as renal function, prevalence of hypertension and diabetes mellitus. On the other hand, age, BMI and NT-proBNP levels were likely to play a role in the associations between cardiac NPs and lipid profile, at least for TC. The association was found to be stronger in older subjects compared to younger ones. This finding could be explained, at least in part, by the fact that older patients are more likely to have both higher NPs levels, caused by cardiac and non-cardiac comorbidities^86^, and lower TC levels, due to lipid-lowering therapy or clinical conditions such as malnutrition and frailty^87,88^. On the other hand, the association between cardiac NPs and TC was weaker in overweight/obese subjects. A possible explanation could be the lower ratio between NPRA and NPRC in the obese subjects that may attenuate the beneficial actions of NPs^89^. It is well known that obese subjects have lower circulating NPs levels^90^. However, the relationship between NPs and obesity is still not fully understood^91–93^, as well as the precise role of obesity in the development of dyslipidemia^94^. Regarding the role of NPs levels, we took into account only NT-proBNP in the sensitivity analysis. Sanchez *et al*.^51^ found that the initial linear association with blood lipids was followed by a tendency towards a plateau at higher NT-proBNP concentrations, given the continuous resetting between active NPs levels and NPRA activity. On the contrary, our extensive meta-analysis confirmed this association even for higher NT-proBNP values.

Our meta-analysis showed a high proportion of observed inconsistency across studies. This could be explained both by the considerable heterogeneity in study population (Supplemental Table 2) and by the quality of the included studies. We identified those studies performed on populations that were likely to interfere with our outcome and grouped them (studies on special populations) in the moderator analysis. The relationship with LDLc, HDLc and TG lost significance if only studies on special populations were taken into account. Same results were found for low-quality studies. Moreover, the inconsistency of the relationship with LDLc and TG decreased from high to moderate after taking into account only those studies on subjects without lipid-lowering therapy, although the association with HDLc lost its significance. Part of the heterogeneity could also be explained by the use of different NP assays in the included studies. We tried to classify them in the sensitivity analyses and the strength of the association differed through the several assays considered. In fact, the assays developed for both BNP and NT-proBNP lack standardization and the results obtained using different assays are not transferable^95^. A significant cross-reactivity among BNP, NT-proBNP, and proBNP peptides, variable for each assay, is present in all the conventional BNP and NT-proBNP immunoassays^96^. A study with mass spectrometry showed that proBNP 1–108 is the most common circulating form detected by the iteration of BNP assays in patients with chronic heart failure^97^. After comparing several NP immunoassays commercially available, the B-type NPs levels differed from one assay to another with a lack of inter-assay transferability. Moreover, also the methods using the same calibrator with or without the same antibodies targeting the same epitopes, did not guarantee an agreement, likely due to a difference in reagent formulation and signal detection, hardware, assay parameters^95^. Although almost all the included studies had a good coefficient of variation, the use of different NP assays is likely one main driver of the heterogeneity of our study results.

### Clinical implications

Our results should stimulate the basic research to better investigate the pathophysiological mechanisms implicated in the regulation of lipid metabolism by NPs and their impact on the circulating lipid levels.

Designer NPs and modulators of cGMP signaling are being developed and investigated for the treatment of the cardiometabolic diseases^98,99^.

Recently, an innovative drug (Sacubitril/Valsartan) that simultaneously inhibits both RAAS and the degradation of NPs (angiotensin II receptor-neprilysin inhibitor, ARNI) has been approved for the treatment of heart failure with reduced ejection fraction^100^. The potential benefits of this drug could go beyond the treatment of heart failure^101^, given the actions of NPs on lipid and glucose metabolism. Recent evidences suggest that all these beneficial effects appear to be driven by a potentiation of ANP rather than BNP^102,103^. Sacubitril/Valsartan improved glycemic control by increasing the insulin-sensitivity and the levels of glucagon-like peptide 1^102,104,105^. On the other side, its effects on lipid metabolism are controversial without apparent benefits on exercise-induced lipolysis and substrate oxidation in obese patients with hypertension^105^. A post-hoc analysis of the PARADIGM-HF trial showed, in addition to a reduction of glycated hemoglobin, an increase in HDLc levels in diabetics receiving Sacubitril/Valsartan compared with those receiving Enalapril^106^. Many factors could play a role in the discrepancy of evidence on this drugs: the other neprilysin substrates that potentially modulate lipid metabolism in the opposite way, the potential anti-lipolytic effects of insulin in adipose tissue, the role played by NPRC (scavenger receptor) in adipocytes, that could be more important than neprilysin activity^105,107^. Further ad hoc trials are needed to clarify the possible beneficial effects of this drug class on dyslipidemia and lipid metabolism.

### Study limitations

Our analysis has some limitations. Firstly, all the included studies were observational investigations and therefore we cannot support any causality between the cardiac NPs levels and the different components of serum lipid profile. Although we performed an extensive review of the main electronic databases, we cannot be sure to have included all relevant studies on this topic. The symmetry of the funnel plots indicates the absence of publication bias that may have influenced our results. However, we found high inconsistency between study findings and high heterogeneity among studies. Therefore, the quality of evidence is moderate at most, and the results of this meta-analysis should be taken with caution. We performed several sensitivity analyses in order to evaluate possible sources of heterogeneity, but we cannot exclude the influence of unmeasured confounding factors. However, taken together, our results show an inverse relationship between NP levels and TC, LDLc and triglyceride, but a positive association with higher HDLc, indicating that a biological positive effect of NPs on lipid profile is very likely and, on the contrary, it’s very unlikely that these results were generated by spurious association due to meta-analysis limitations or by chance. Our meta-analysis focused on TC, LDLc, HDLc and TG. The presence in literature of only one article regarding NPs and ApoB^21^ did not allow us to include this lipid parameter.

## Conclusions

The present systematic review and meta-analysis of 46 studies highlights an association between higher cardiac NPs levels and a more favorable lipid profile. This both confirms and extends our understanding of the metabolic properties of cardiac NPs and their potential in CV prevention.

## Supplementary information

Supplementary Information

## References

[1] Mukoyama M (1990). Human brain natriuretic peptide, a novel cardiac hormone. Lancet.

[2] Suga S (1992). Endothelial production of C-type natriuretic peptide and its marked augmentation by transforming growth factor-beta. Possible existence of “vascular natriuretic peptide system”. The Journal of clinical investigation.

[3] Volpe M (2014). Natriuretic peptides and cardio-renal disease. International journal of cardiology.

[4] Sarzani R, Salvi F, Dessi-Fulgheri P, Rappelli A (2008). Renin-angiotensin system, natriuretic peptides, obesity, metabolic syndrome, and hypertension: an integrated view in humans. Journal of hypertension.

[5] Sarzani R (2017). Cardiac Natriuretic Peptides, Hypertension and Cardiovascular Risk. High blood pressure & cardiovascular prevention: the official journal of the Italian Society of Hypertension.

[6] Schlueter N (2014). Metabolic actions of natriuretic peptides and therapeutic potential in the metabolic syndrome. Pharmacology & therapeutics.

[7] Bordicchia M (2016). Insulin/glucose induces natriuretic peptide clearance receptor in human adipocytes: a metabolic link with the cardiac natriuretic pathway. American journal of physiology. Regulatory, integrative and comparative physiology.

[8] Volpe M, Rubattu S, Burnett J (2014). Natriuretic peptides in cardiovascular diseases: current use and perspectives. European heart journal.

[9] McKie PM, Burnett JC (2016). NT-proBNP: The Gold Standard Biomarker in Heart Failure. Journal of the American College of Cardiology.

[10] Bordicchia Marica, Spannella Francesco, Ferretti Gianna, Bacchetti Tiziana, Vignini Arianna, Di Pentima Chiara, Mazzanti Laura, Sarzani Riccardo (2019). PCSK9 is Expressed in Human Visceral Adipose Tissue and Regulated by Insulin and Cardiac Natriuretic Peptides. International Journal of Molecular Sciences.

[11] Musani SK (2013). Aldosterone, C-reactive protein, and plasma B-type natriuretic peptide are associated with the development of metabolic syndrome and longitudinal changes in metabolic syndrome components: findings from the Jackson Heart Study. Diabetes care.

[12] Cannone V (2011). A genetic variant of the atrial natriuretic peptide gene is associated with cardiometabolic protection in the general community. Journal of the American College of Cardiology.

[13] Stroup DF (2000). Meta-analysis of observational studies in epidemiology: a proposal for reporting. Meta-analysis Of Observational Studies in Epidemiology (MOOSE) group. Jama.

[14] Wan X, Wang W, Liu J, Tong T (2014). Estimating the sample mean and standard deviation from the sample size, median, range and/or interquartile range. BMC medical research methodology.

[15] Wells G. *et al*. The Newcastle-Ottawa Scale (NOS) for assessing the quality of nonrandomized studies in meta-analyses. The Ottawa Hospital website, http://www.ohri.ca/programs/clinical_epidemiology/oxford.asp. Published 2018. Accessed September 4 (2018).

[16] Pranger IG (2019). Fatty acids as biomarkers of total dairy and dairy fat intakes: a systematic review and meta-analysis. Nutrition reviews.

[17] Borenstein, M., Hedges, L. V., Higgins, J. P. T. & Rothstein, H. R. Introduction to Meta-Analysis. Chichester, UK: John Wiley and Sons, Ltd; 2009; Lipsey MW, Wilson DB, eds. Practical Meta-Analysis. Vol 49. Thousand Oaks, CA: Sage (2001).

[18] Higgins JP, Thompson SG, Deeks JJ, Altman DG (2003). Measuring inconsistency in meta-analyses. Bmj.

[19] Sterne JA, Egger M (2001). Funnel plots for detecting bias in meta-analysis: guidelines on choice of axis. Journal of clinical epidemiology.

[20] Olsen MH (2005). N-terminal pro brain natriuretic peptide is inversely related to metabolic cardiovascular risk factors and the metabolic syndrome. Hypertension.

[21] Akanji AO, Suresh CG, Al-Radwan R, Fatania HR (2009). Body mass and atherogenic dyslipidemia as major determinants of blood levels of B-type natriuretic peptides in Arab subjects with acute coronary syndromes. Metab Syndr Relat Disord.

[22] Bao Y (2011). Relationship between N-terminal pro-B-type natriuretic peptide levels and metabolic syndrome. Arch Med Sci.

[23] Brutsaert EF (2016). Longitudinal assessment of N-terminal pro-B-type natriuretic peptide and risk of diabetes in older adults: The cardiovascular health study. Metabolism.

[24] Chang HR (2013). Inverse association of N-terminal pro-B-type natriuretic peptide with metabolic syndrome in patients with congestive heart failure. PloS one.

[25] Chang HR (2014). N-terminal pro-B-type natriuretic peptide is inversely associated with metabolic syndrome in hypertensive patients. The American journal of the medical sciences.

[26] Chen SF (2016). Impact of Protein Nutritional Status on Plasma BNP in Elderly Patients. J Nutr Health Aging.

[27] Cushman M (2014). N-terminal pro-B-type natriuretic peptide and stroke risk: the reasons for geographic and racial differences in stroke cohort. Stroke.

[28] Daniels LB (2011). Elevated natriuretic peptide levels and cognitive function in community-dwelling older adults. Am J Med.

[29] Fu S, Ping P, Luo L, Ye P (2016). Deep analyses of the associations of a series of biomarkers with insulin resistance, metabolic syndrome, and diabetes risk in nondiabetic middle-aged and elderly individuals: results from a Chinese community-based study. Clin Interv Aging.

[30] Goharian TS (2017). Associations of Proatrial Natriuretic Peptide with Components of the Metabolic Syndrome in Adolescents and Young Adults from the General Population. Am J Hypertens.

[31] Greene SJ (2013). Prognostic significance of serum total cholesterol and triglyceride levels in patients hospitalized for heart failure with reduced ejection fraction (from the EVEREST Trial). The American journal of cardiology.

[32] He WT, Mori M, Yu XF, Kanda T (2016). Higher BNP levels within physiological range correlate with beneficial nonfasting lipid profiles in the elderly: a cross-sectional study. Lipids in health and disease.

[33] Hong SN (2008). N-terminal pro-B-type natriuretic peptide level is depressed in patients with significant coronary artery disease who have high body mass index. Int Heart J.

[34] Hsieh JC (2013). Low serum long-acting natriuretic peptide level correlates with metabolic syndrome in hypertensive patients: a cross-sectional study. Arch Med Res.

[35] Kawase S (2015). Plasma Brain Natriuretic Peptide is a Marker of Prognostic Functional Outcome in Non-Cardioembolic Infarction. J Stroke Cerebrovasc Dis.

[36] Lauria PB, Del Puerto HL, Reis AM, Candido AL, Reis FM (2013). Low plasma atrial natriuretic peptide: a new piece in the puzzle of polycystic ovary syndrome. J Clin Endocrinol Metab.

[37] Lazo M (2013). NH2-terminal pro-brain natriuretic peptide and risk of diabetes. Diabetes.

[38] Lee KM, Lee MC, Lee CJ, Chen YC, Hsu BG (2018). Inverse Association of N-terminal ProB-type Natriuretic Peptide Level With Metabolic Syndrome in Kidney Transplant Patients. Transplant Proc.

[39] Li J (2018). Effects of serum N-terminal pro B-type natriuretic peptide and D-dimer levels on patients with acute ischemic stroke. Pakistan journal of medical sciences.

[40] Li WY, Chiu FC, Chien YF, Lin JW, Hwang JJ (2011). Association of amino-terminal pro-brain natriuretic peptide with metabolic syndrome. Intern Med.

[41] Mansoor A (2009). Elevated NT-pro-BNP levels are associated with comorbidities among HIV-infected women. AIDS research and human retroviruses.

[42] Mizuno Y (2013). Cardiac production of B-type natriuretic peptide is inversely related to the plasma level of free fatty acids in obese individuals - possible involvement of the insulin resistance. Endocrine journal.

[43] Murphy CA (2018). Excessive Adiposity and Metabolic Dysfunction Relate to Reduced Natriuretic Peptide During RAAS Activation in HIV. J Clin Endocrinol Metab.

[44] Nakatsuji H, Kishida K, Funahashi T, Nakagawa T, Shimomura I (2012). Hyperinsulinemia correlates with low levels of plasma B-type natriuretic peptide in Japanese men irrespective of fat distribution. Cardiovascular diabetology.

[45] Nayak BS (2015). Evaluation of N-terminal pro-B-type natriuretic peptide and high-sensitivity C-reactive protein relationship with features of metabolic syndrome in high-risk subgroups for cardiovascular disease. Int J Appl Basic Med Res.

[46] Olalla J (2015). Factors related to NT-proBNP levels in HIV patients aged over 40 years. AIDS Res Ther.

[47] Oztekin S, Karakurt O, Yazihan N, Unal I (2011). Relationship of brain natriuretic peptide with metabolic syndrome parameters: an observational study. Anadolu Kardiyol Derg.

[48] Price AH (2014). N-terminal pro-brain natriuretic peptide and risk of cardiovascular events in older patients with type 2 diabetes: the Edinburgh Type 2 Diabetes Study. Diabetologia.

[49] Ribeiro A (2015). Predictors of natriuretic peptide non-response in patients hospitalized with acute heart failure. Am J Cardiol.

[50] Ricci MA (2017). Determinants of low levels of brain natriuretic peptide in morbid obesity. Clin Nutr.

[51] Sanchez OA (2014). The associations between metabolic variables and NT-proBNP are blunted at pathological ranges: the Multi-Ethnic Study of Atherosclerosis. Metabolism.

[52] Sezen Y (2009). N-terminal pro-brain natriuretic peptide in cases with metabolic syndrome and its relationship with components of metabolic syndrome and left ventricular mass index. Clin Biochem.

[53] Shivananda Nayak B, Teelucksingh S, Jagessar A, Maharaj S, Maharaj N (2013). A cross sectional study comparing traditional risk factors with N-terminal pro-BNP in high risk groups for cardiovascular disease in Trinidad, West Indies. Diabetes Metab Syndr.

[54] Siervo M (2010). Angiogenesis and biomarkers of cardiovascular risk in adults with metabolic syndrome. Journal of internal medicine.

[55] Spannella F (2018). N-terminal pro B-Type natriuretic peptide is inversely correlated with low density lipoprotein cholesterol in the very elderly. Nutr Metab Cardiovasc Dis.

[56] Takeuchi H, Sata M (2012). The relationship among brain natriuretic peptide (BNP), cholesterol and lipoprotein. Heart Asia.

[57] Tanaka A (2017). N-terminal pro-brain natriuretic peptide and associated factors in the general working population: a baseline survey of the Uranosaki cohort study. Scientific reports.

[58] Theilade S, Hansen TW, Goetze JP, Rossing P (2015). Increased plasma concentrations of midregional proatrial natriuretic Peptide is associated with risk of cardiorenal dysfunction in type 1 diabetes. Am J Hypertens.

[59] Then C (2013). Plasma MR-proANP levels are associated with carotid intima-media thickness in the general community: the KORA F4 study. Atherosclerosis.

[60] Tsai, J. P. L. C., Wang, C. H., Lai, Y. H., Lin, Y. L., Hsu, B. G. Inverse association of long-acting natriuretic peptide with metabolic syndrome in peritoneal dialysis patients. *Int J Clin Exp Pathol* **9** (2016).

[61] Wang JH, Lee CJ, Hsieh JC, Chen YC, Hsu BG (2014). N-terminal pro-B-type natriuretic peptide level inversely associates with metabolic syndrome in elderly persons. Diabetology & metabolic syndrome.

[62] Wang TJ (2007). Association of plasma natriuretic peptide levels with metabolic risk factors in ambulatory individuals. Circulation.

[63] Yuan J, Li LI, Wang Z, Song W, Zhang Z (2016). Dyslipidemia in patients with systemic lupus erythematosus: Association with disease activity and B-type natriuretic peptide levels. Biomed Rep.

[64] Zhu WH (2016). Correlation between B type natriuretic peptide and metabolic risk factors. Arch Med Sci.

[65] Agra Bermejo RM (2017). Nutritional status is related to heart failure severity and hospital readmissions in acute heart failure. Int J Cardiol.

[66] Everett BM, Zeller T, Glynn RJ, Ridker PM, Blankenberg S (2015). High-sensitivity cardiac troponin I and B-type natriuretic Peptide as predictors of vascular events in primary prevention: impact of statin therapy. Circulation.

[67] Masuch A (2018). Metabolomic profiling implicates adiponectin as mediator of a favorable lipoprotein profile associated with NT-proBNP. Cardiovascular diabetology.

[68] Dai Y (2017). In-hospital and long-term outcomes of congestive heart failure: Predictive value of B-type and amino-terminal pro-B-type natriuretic peptides and their ratio. Experimental and therapeutic medicine.

[69] Spannella Francesco, Giulietti Federico, Di Pentima Chiara, Sarzani Riccardo (2019). Prevalence and Control of Dyslipidemia in Patients Referred for High Blood Pressure: The Disregarded “Double-Trouble” Lipid Profile in Overweight/Obese. Advances in Therapy.

[70] Catapano AL (2016). ESC/EAS Guidelines for the Management of Dyslipidaemias. European heart journal.

[71] Emerging Risk Factors C (2009). Major lipids, apolipoproteins, and risk of vascular disease. Jama.

[72] Jordan J, Birkenfeld AL, Melander O, Moro C (2018). Natriuretic Peptides in Cardiovascular and Metabolic Crosstalk: Implications for Hypertension Management. Hypertension.

[73] Cannone V (2013). The atrial natriuretic peptide genetic variant rs5068 is associated with a favorable cardiometabolic phenotype in a Mediterranean population. Diabetes care.

[74] Cannone V (2017). A favorable cardiometabolic profile is associated with the G allele of the genetic variant rs5068 in African Americans: The Multi-Ethnic Study of Atherosclerosis (MESA). PLoS One.

[75] Liang F (2007). B-Type natriuretic peptide inhibited angiotensin II-stimulated cholesterol biosynthesis, cholesterol transfer, and steroidogenesis in primary human adrenocortical cells. Endocrinology.

[76] Sarzani R, Dessi-Fulgheri P, Paci VM, Espinosa E, Rappelli A (1996). Expression of natriuretic peptide receptors in human adipose and other tissues. Journal of endocrinological investigation.

[77] Bordicchia M (2012). Cardiac natriuretic peptides act via p38 MAPK to induce the brown fat thermogenic program in mouse and human adipocytes. The Journal of clinical investigation.

[78] Khedoe PP (2015). Brown adipose tissue takes up plasma triglycerides mostly after lipolysis. Journal of lipid research.

[79] Miyashita K (2009). Natriuretic peptides/cGMP/cGMP-dependent protein kinase cascades promote muscle mitochondrial biogenesis and prevent obesity. Diabetes.

[80] Pfeifer A, Kilic A, Hoffmann LS (2013). Regulation of metabolism by cGMP. Pharmacology & therapeutics.

[81] Hoffmann LS (2015). Stimulation of soluble guanylyl cyclase protects against obesity by recruiting brown adipose tissue. Nature communications.

[82] Prickett TCR, Lunt H, Warwick J, Heenan HF, Espiner EA (2019). Urinary Amino-Terminal Pro-C-Type Natriuretic Peptide: A Novel Marker of Chronic Kidney Disease in Diabetes. Clinical chemistry.

[83] Spiranec K (2018). Endothelial C-Type Natriuretic Peptide Acts on Pericytes to Regulate Microcirculatory Flow and Blood Pressure. Circulation.

[84] Bae CR (2017). Overexpression of C-type Natriuretic Peptide in Endothelial Cells Protects against Insulin Resistance and Inflammation during Diet-induced Obesity. Scientific reports.

[85] Bae CR (2018). Adipocyte-specific expression of C-type natriuretic peptide suppresses lipid metabolism and adipocyte hypertrophy in adipose tissues in mice fed high-fat diet. Scientific reports.

[86] Sarzani R (2016). NT-proBNP and Its Correlation with In-Hospital Mortality in the Very Elderly without an Admission Diagnosis of Heart Failure. PloS one.

[87] Volpato S, Palmieri E, Fellin R, Zuliani G (2000). Acute phase markers are associated with reduced plasma lipid levels in a population of hospitalized elderly patients. Gerontology.

[88] Rockwood K, McMillan M, Mitnitski A, Howlett SE (2015). A Frailty Index Based on Common Laboratory Tests in Comparison With a Clinical Frailty Index for Older Adults in Long-Term Care Facilities. Journal of the American Medical Directors Association.

[89] Kovacova Z (2016). Adipose tissue natriuretic peptide receptor expression is related to insulin sensitivity in obesity and diabetes. Obesity.

[90] Wang TJ (2004). Impact of obesity on plasma natriuretic peptide levels. Circulation.

[91] Collins S (2014). A heart-adipose tissue connection in the regulation of energy metabolism. Nature reviews. Endocrinology.

[92] Glode A (2017). Divergent effects of a designer natriuretic peptide CD-NP in the regulation of adipose tissue and metabolism. Molecular metabolism.

[93] Mueller C (2019). Heart Failure Association of the European Society of Cardiology practical guidance on the use of natriuretic peptide concentrations. European journal of heart failure.

[94] Kotsis V (2018). Obesity and cardiovascular risk: a call for action from the European Society of Hypertension Working Group of Obesity, Diabetes and the High-risk Patient and European Association for the Study of Obesity: part A: mechanisms of obesity induced hypertension, diabetes and dyslipidemia and practice guidelines for treatment. Journal of hypertension.

[95] Collin-Chavagnac D (2015). Head-to-head comparison of 10 natriuretic peptide assays. Clinical chemistry and laboratory medicine.

[96] Saenger AK (2017). Specificity of B-Type Natriuretic Peptide Assays: Cross-Reactivity with Different BNP, NT-proBNP, and proBNP Peptides. Clinical chemistry.

[97] Miller WL (2011). Comparison of mass spectrometry and clinical assay measurements of circulating fragments of B-type natriuretic peptide in patients with chronic heart failure. Circulation. Heart failure.

[98] Meems LMG, Burnett JC (2016). Innovative Therapeutics: Designer Natriuretic Peptides. JACC. Basic to translational science.

[99] Jordan J, Hildebrand S, Pfeifer A (2019). cGMP manipulation in cardiometabolic disease: chances and challenges. Current opinion in cardiology.

[100] McMurray JJ (2014). Angiotensin-neprilysin inhibition versus enalapril in heart failure. The New England journal of medicine.

[101] Spannella Francesco, Marini Marco, Giulietti Federico, Rosettani Giulia, Francioni Matteo, Perna Gian Piero, Sarzani Riccardo (2019). Renal effects of Sacubitril/Valsartan in heart failure with reduced ejection fraction: a real life 1-year follow-up study. Internal and Emergency Medicine.

[102] Nougue H (2019). Effects of sacubitril/valsartan on neprilysin targets and the metabolism of natriuretic peptides in chronic heart failure: a mechanistic clinical study. European journal of heart failure.

[103] Ibrahim NE (2019). Effect of Neprilysin Inhibition on Various Natriuretic Peptide Assays. Journal of the American College of Cardiology.

[104] Jordan J (2017). Improved Insulin Sensitivity With Angiotensin Receptor Neprilysin Inhibition in Individuals With Obesity and Hypertension. Clinical pharmacology and therapeutics.

[105] Engeli S (2018). Effect of Sacubitril/Valsartan on Exercise-Induced Lipid Metabolism in Patients With Obesity and Hypertension. Hypertension.

[106] Seferovic JP (2017). Effect of sacubitril/valsartan versus enalapril on glycaemic control in patients with heart failure and diabetes: a post-hoc analysis from the PARADIGM-HF trial. The lancet. Diabetes & endocrinology.

[107] Moro C, Klimcakova E, Lafontan M, Berlan M, Galitzky J (2007). Phosphodiesterase-5A and neutral endopeptidase activities in human adipocytes do not control atrial natriuretic peptide-mediated lipolysis. British journal of pharmacology.

