# Supplementary material for: Association Between Cardiac Natriuretic Peptides and Lipid Profile: a Systematic Review and Meta-Analysis
Source: Sci Rep. 2019 Dec 16;9:19178. doi: 10.1038/s41598-019-55680-z (PMC6915780; doi:10.1038/s41598-019-55680-z)

Supplemental Methods. Search strategy used for PubMed (Medline) and Scopus.

Keywords:

1- Brain natriuretic peptide

2- B-type natriuretic peptide

3- BNP

4- NTproBNP

5- NT-proBNP

6- pro-BNP

7- proBNP

8- N-terminal pro B-type natriuretic peptide

9- NT-proBNP/BNP ratio

10- ratio of NT-proBNP to BNP

11- natriuretic peptides

12- Natriuretic peptides ratio

13- Atrial natriuretic peptide

14- A-type natriuretic peptide

15- ANP

16- NT-proANP

17- NTproANP

18- pro-ANP

19- proANP

20- MR-proANP

21- plasma cholesterol

22- lipid profile

23- lipoprotein

24- cholesterol

25- LDL

26- HDL

27- Triglycerides

28- Non-HDL

29- total cholesterol

30- Low-density lipoprotein cholesterol

31- High-density lipoprotein cholesterol

32- lipid panel

33- lipid metabolism

Search strategy

(1 OR 2 OR 3 OR 4 OR 5 OR 6 OR 7 OR 8 OR 9 OR 10 OR 11 OR 12 OR 13 OR 14 OR 15 OR 16 OR 17 OR 18 OR 19 OR 20) AND (21 OR 22 OR 23 OR 24 OR 25 OR 26 OR 27 OR 28 OR 29 OR 30 OR 31 OR 32 OR 33)

Supplemental table 1. Results of the quality assessment of the 46 studies included in the meta-analysis.

| Reference | Selection score (maximum 3 stars) | Comparability score (maximum 1 star) | Outcome score (maximum 2 stars) | Total score (maximum 6 stars) |
|---|---|---|---|---|
| Akanji AO et al 2009 [1] | * | | ** | 3 |
| Bao Y et al 2011 [2] | *** | * | ** | 6 |
| Brutsaert EF et al 2016 [3] | *** | | ** | 5 |
| Chang HR et al 2013 [4] | * | | ** | 3 |
| Chang HR et al 2014 [5] | * | | ** | 3 |
| Chen SF et al 2016 [6] | *** | * | ** | 6 |
| Cushman M et al 2014 [7] | *** | * | ** | 6 |
| Daniels LB et al 2011 [8] | *** | | * | 4 |
| Everett BM et al [9] | *** | * | ** | 6 |
| Fu S et al 2016 [10] | ** | * | * | 4 |
| Goharian TS et al 2017 [11] | ** | | * | 3 |
| Greene SJ et al 2013 [12] | ** | | ** | 4 |
| He WT et al 2016 [13] | ** | * | ** | 5 |
| Hong et al 2008 [14] | * | | * | 2 |
| Hsieh et al 2013 [15] | ** | | ** | 4 |
| Kawase S et al 2015 [16] | | * | ** | 3 |
| Lauria PB et al 2013 [17] | * | * | ** | 4 |
| Lazo M et al 2013 [18] | *** | | ** | 5 |

| | | | | |
|---|---|---|---|---|
| Lee KM et al 2018 [19] | * | | ** | 3 |
| Li J et al 2018 [20] | ** | | ** | 4 |
| Li WY et al 2011 [21] | *** | * | ** | 6 |
| Mansoor A et al 2009 [22] | * | | ** | 3 |
| Mizuno Y et al 2013 [23] | * | | ** | 3 |
| Murphy CA et al 2018 [24] | * | * | * | 3 |
| Nakatsuji H et al 2012 [25] | ** | | ** | 4 |
| Nayak BS et al 2015 [26] | * | | * | 2 |
| Olalla J et al 2015 [27] | * | | | 1 |
| Olsen et al 2005 [28] | *** | * | * | 5 |
| Oztekin S et al 2011 [29] | * | | * | 2 |
| Price AH et al 2014 [30] | *** | | ** | 5 |
| Ribeiro A et al 2015 [31] | *** | | * | 4 |
| Ricci MA et al 2017 [32] | ** | | ** | 4 |
| Sanchez OA et al 2014 [33] | *** | | ** | 5 |
| Sezen Y. et al 2009 [34] | ** | * | ** | 5 |
| Shivananda Nayak B et al 2013 [35] | ** | * | * | 4 |
| Siervo M et al 2010 [36] | *** | * | ** | 6 |
| Spannella F et al 2018 [37] | *** | * | ** | 6 |
| Takeuchi H et al 2012 [38] | | | * | 1 |
| Tanaka A et al 2017 [39] | *** | | ** | 5 |
| Theilade S et al 2015 [40] | ** | * | ** | 5 |

| | | | | |
|---|---|---|---|---|
| Then C et al 2013 [41] | *** | | * | 4 |
| Tsai JP et al 2016 [42] | * | | ** | 3 |
| Wang JH et al 2014 [43] | ** | * | ** | 5 |
| Wang TJ et al 2007 [44] | *** | * | ** | 6 |
| Yuan J et al 2016 [45] | * | | * | 2 |
| Zhu WH et al 2016 [46] | *** | * | * | 5 |

According to the adapted Newcastle-Ottawa Scale (NOS) for assessing the quality of nonrandomized studies in meta-analyses. The maximum attainable quality score is 6. High quality studies were identified by a score $\geq 5$.

**REFERENCES**


1 Akanji, A. O., Suresh, C. G., Al-Radwan, R. & Fatania, H. R. Body mass and atherogenic dyslipidemia as major determinants of blood levels of B-type natriuretic peptides in Arab subjects with acute coronary syndromes. *Metabolic syndrome and related disorders* **7**, 563-569, doi:10.1089/met.2009.0034 (2009).

2 Bao, Y. *et al.* Relationship between N-terminal pro-B-type natriuretic peptide levels and metabolic syndrome. *Archives of medical science : AMS* **7**, 247-256, doi:10.5114/aoms.2011.22075 (2011).

3 Brutsaert, E. F. *et al.* Longitudinal assessment of N-terminal pro-B-type natriuretic peptide and risk of diabetes in older adults: The cardiovascular health study. *Metabolism: clinical and experimental* **65**, 1489-1497, doi:10.1016/j.metabol.2016.06.002 (2016).

4 Chang, H. R. *et al.* Inverse association of N-terminal pro-B-type natriuretic peptide with metabolic syndrome in patients with congestive heart failure. *PloS one* **8**, e79096, doi:10.1371/journal.pone.0079096 (2013).

5 Chang, H. R. *et al.* N-terminal pro-B-type natriuretic peptide is inversely associated with metabolic syndrome in hypertensive patients. *The American journal of the medical sciences* **348**, 210-214, doi:10.1097/MAJ.0000000000000234 (2014).

6 Chen, S. F. *et al.* Impact of Protein Nutritional Status on Plasma BNP in Elderly Patients. *The journal of nutrition, health & aging* **20**, 937-943, doi:10.1007/s12603-016-0716-z (2016).

7 Cushman, M. *et al.* N-terminal pro-B-type natriuretic peptide and stroke risk: the reasons for geographic and racial differences in stroke cohort. *Stroke* **45**, 1646-1650, doi:10.1161/STROKEAHA.114.004712 (2014).

8 Daniels, L. B. *et al.* Elevated natriuretic peptide levels and cognitive function in community-dwelling older adults. *The American journal of medicine* **124**, 670 e671-678, doi:10.1016/j.amjmed.2011.02.027 (2011).

9 Everett, B. M., Zeller, T., Glynn, R. J., Ridker, P. M. & Blankenberg, S. High-sensitivity cardiac troponin I and B-type natriuretic Peptide as predictors of vascular events in primary prevention: impact of statin therapy. *Circulation* **131**, 1851-1860, doi:10.1161/CIRCULATIONAHA.114.014522 (2015).

10 Fu, S., Ping, P., Luo, L. & Ye, P. Deep analyses of the associations of a series of biomarkers with insulin resistance, metabolic syndrome, and diabetes risk in nondiabetic middle-aged and elderly individuals: results from a Chinese community-based study. *Clinical interventions in aging* **11**, 1531-1538, doi:10.2147/CIA.S109583 (2016).

11 Goharian, T. S. *et al.* Associations of Proatrial Natriuretic Peptide with Components of the Metabolic Syndrome in Adolescents and Young Adults from the General Population. *American journal of hypertension* **30**, 561-568, doi:10.1093/ajh/hpx026 (2017).

12 Greene, S. J. *et al.* Prognostic significance of serum total cholesterol and triglyceride levels in patients hospitalized for heart failure with reduced ejection fraction (from the EVEREST Trial). *The American journal of cardiology* **111**, 574-581, doi:10.1016/j.amjcard.2012.10.042 (2013).

13 He, W. T., Mori, M., Yu, X. F. & Kanda, T. Higher BNP levels within physiological range correlate with beneficial nonfasting lipid profiles in the elderly: a cross-sectional study. *Lipids in health and disease* **15**, 3, doi:10.1186/s12944-015-0168-1 (2016).

14 Hong, S. N. *et al.* N-terminal pro-B-type natriuretic peptide level is depressed in patients with significant coronary artery disease who have high body mass index. *International heart journal* **49**, 403-412 (2008).

15 Hsieh, J. C. *et al.* Low serum long-acting natriuretic peptide level correlates with metabolic syndrome in hypertensive patients: a cross-sectional study. *Archives of medical research* **44**, 215-220, doi:10.1016/j.arcmed.2013.03.001 (2013).

16 Kawase, S. *et al.* Plasma Brain Natriuretic Peptide is a Marker of Prognostic Functional Outcome in Non-Cardioembolic Infarction. *Journal of stroke and cerebrovascular diseases : the official journal of National Stroke Association* **24**, 2285-2290, doi:10.1016/j.jstrokecerebrovasdis.2015.06.006 (2015).

17 Lauria, P. B., Del Puerto, H. L., Reis, A. M., Candido, A. L. & Reis, F. M. Low plasma atrial natriuretic peptide: a new piece in the puzzle of polycystic ovary syndrome. *The Journal of clinical endocrinology and metabolism* **98**, 4882-4889, doi:10.1210/jc.2013-2141 (2013).

18 Lazo, M. *et al.* NH2-terminal pro-brain natriuretic peptide and risk of diabetes. *Diabetes* **62**, 3189-3193, doi:10.2337/db13-0478 (2013).

19 Lee, K. M., Lee, M. C., Lee, C. J., Chen, Y. C. & Hsu, B. G. Inverse Association of N-terminal ProB-type Natriuretic Peptide Level With Metabolic Syndrome in Kidney Transplant Patients. *Transplantation proceedings* **50**, 2496-2501, doi:10.1016/j.transproceed.2018.04.005 (2018).

20 Li, J. *et al.* Effects of serum N-terminal pro B-type natriuretic peptide and D-dimer levels on patients with acute ischemic stroke. *Pakistan journal of medical sciences* **34**, 994-998, doi:10.12669/pjms.344.15432 (2018).

21 Li, W. Y., Chiu, F. C., Chien, Y. F., Lin, J. W. & Hwang, J. J. Association of amino-terminal pro-brain natriuretic peptide with metabolic syndrome. *Internal medicine* **50**, 1143-1147 (2011).

22 Mansoor, A. *et al.* Elevated NT-pro-BNP levels are associated with comorbidities among HIV-infected women. *AIDS research and human retroviruses* **25**, 997-1004, doi:10.1089/aid.2009.0038 (2009).

23 Mizuno, Y. *et al.* Cardiac production of B-type natriuretic peptide is inversely related to the plasma level of free fatty acids in obese individuals - possible involvement of the insulin resistance. *Endocrine journal* **60**, 87-95 (2013).

24 Murphy, C. A. *et al.* Excessive Adiposity and Metabolic Dysfunction Relate to Reduced Natriuretic Peptide During RAAS Activation in HIV. *The Journal of clinical endocrinology and metabolism* **103**, 1558-1565, doi:10.1210/jc.2017-02198 (2018).

25 Nakatsuji, H., Kishida, K., Funahashi, T., Nakagawa, T. & Shimomura, I. Hyperinsulinemia correlates with low levels of plasma B-type natriuretic peptide in Japanese men irrespective of fat distribution. *Cardiovascular diabetology* **11**, 22, doi:10.1186/1475-2840-11-22 (2012).

26 Nayak, B. S. *et al.* Evaluation of N-terminal pro-B-type natriuretic peptide and high-sensitivity C-reactive protein relationship with features of metabolic syndrome in high-risk subgroups for cardiovascular disease. *International journal of applied & basic medical research* **5**, 190-194, doi:10.4103/2229-516X.165369 (2015).

27 Olalla, J. *et al.* Factors related to NT-proBNP levels in HIV patients aged over 40 years. *AIDS research and therapy* **12**, 17, doi:10.1186/s12981-015-0058-7 (2015).

28 Olsen, M. H. *et al.* N-terminal pro brain natriuretic peptide is inversely related to metabolic cardiovascular risk factors and the metabolic syndrome. *Hypertension* **46**, 660-666, doi:10.1161/01.HYP.0000179575.13739.72 (2005).

29 Oztekin, S., Karakurt, O., Yazihan, N. & Unal, I. Relationship of brain natriuretic peptide with metabolic syndrome parameters: an observational study. *Anadolu kardiyoloji dergisi : AKD = the Anatolian journal of cardiology* **11**, 678-684, doi:10.5152/akd.2011.188 (2011).

30 Price, A. H. *et al.* N-terminal pro-brain natriuretic peptide and risk of cardiovascular events in older patients with type 2 diabetes: the Edinburgh Type 2 Diabetes Study. *Diabetologia* **57**, 2505-2512, doi:10.1007/s00125-014-3375-9 (2014).

31 Ribeiro, A. *et al.* Predictors of natriuretic peptide non-response in patients hospitalized with acute heart failure. *The American journal of cardiology* **115**, 69-74, doi:10.1016/j.amjcard.2014.09.053 (2015).

32 Ricci, M. A. *et al.* Determinants of low levels of brain natriuretic peptide in morbid obesity. *Clinical nutrition* **36**, 1075-1081, doi:10.1016/j.clnu.2016.06.024 (2017).

33 Sanchez, O. A. *et al.* The associations between metabolic variables and NT-proBNP are blunted at pathological ranges: the Multi-Ethnic Study of Atherosclerosis. *Metabolism: clinical and experimental* **63**, 475-483, doi:10.1016/j.metabol.2013.11.017 (2014).

34 Sezen, Y. *et al.* N-terminal pro-brain natriuretic peptide in cases with metabolic syndrome and its relationship with components of metabolic syndrome and left ventricular mass index. *Clinical biochemistry* **42**, 1500-1503, doi:10.1016/j.clinbiochem.2009.07.007 (2009).

35 Shivananda Nayak, B., Teelucksingh, S., Jagessar, A., Maharaj, S. & Maharaj, N. A cross sectional study comparing traditional risk factors with N-terminal pro-BNP in high risk groups for cardiovascular disease in Trinidad, West Indies. *Diabetes & metabolic syndrome* **7**, 8-11, doi:10.1016/j.dsx.2013.02.021 (2013).

36 Siervo, M. *et al.* Angiogenesis and biomarkers of cardiovascular risk in adults with metabolic syndrome. *Journal of internal medicine* **268**, 338-347, doi:10.1111/j.1365-2796.2010.02255.x (2010).

37 Spannella, F. *et al.* N-terminal pro B-Type natriuretic peptide is inversely correlated with low density lipoprotein cholesterol in the very elderly. *Nutrition, metabolism, and cardiovascular diseases : NMCD* **28**, 629-635, doi:10.1016/j.numecd.2018.02.013 (2018).

38 Takeuchi, H. & Sata, M. The relationship among brain natriuretic peptide (BNP), cholesterol and lipoprotein. *Heart Asia* **4**, 11-15, doi:10.1136/heartasia-2011-010042 (2012).

39 Tanaka, A. *et al.* N-terminal pro-brain natriuretic peptide and associated factors in the general working population: a baseline survey of the Uranosaki cohort study. *Scientific reports* **7**, 5810, doi:10.1038/s41598-017-06090-6 (2017).

40 Theilade, S., Hansen, T. W., Goetze, J. P. & Rossing, P. Increased plasma concentrations of midregional proatrial natriuretic Peptide is associated with risk of cardiorenal dysfunction in type 1 diabetes. *American journal of hypertension* **28**, 772-779, doi:10.1093/ajh/hpu227 (2015).

41 Then, C. *et al.* Plasma MR-proANP levels are associated with carotid intima-media thickness in the general community: the KORA F4 study. *Atherosclerosis* **230**, 235-241, doi:10.1016/j.atherosclerosis.2013.07.047 (2013).

42 Tsai JP, L. C., Wang CH, Lai YH, Lin YL, Hsu BG. Inverse association of long-acting natriuretic peptide with metabolic syndrome in peritoneal dialysis patients. *Int J Clin Exp Pathol* **9** (2016).

43 Wang, J. H., Lee, C. J., Hsieh, J. C., Chen, Y. C. & Hsu, B. G. N-terminal pro-B-type natriuretic peptide level inversely associates with metabolic syndrome in elderly persons. *Diabetology & metabolic syndrome* **6**, 15, doi:10.1186/1758-5996-6-15 (2014).

44 Wang, T. J. *et al.* Association of plasma natriuretic peptide levels with metabolic risk factors in ambulatory individuals. *Circulation* **115**, 1345-1353, doi:10.1161/CIRCULATIONAHA.106.655142 (2007).

45 Yuan, J., Li, L. I., Wang, Z., Song, W. & Zhang, Z. Dyslipidemia in patients with systemic lupus erythematosus: Association with disease activity and B-type natriuretic peptide levels. *Biomedical reports* **4**, 68-72, doi:10.3892/br.2015.544 (2016).

46 Zhu, W. H. *et al.* Correlation between B type natriuretic peptide and metabolic risk factors. *Archives of medical science : AMS* **12**, 334-340, doi:10.5114/aoms.2015.57001 (2016).

Supplemental table 2. Characteristics of the 46 studies included in the meta-analysis

| Study | Design | Participants | Sample size | Age (years) | Sex (%, male) | Type of NP analyzed | NP levels | Type of lipids analyzed | TC levels (mg/dl) | LDLc levels (mg/dl) | HDLc levels (mg/dl) | TG levels (mg/dl) |
|---|---|---|---|---|---|---|---|---|---|---|---|---|
| Akanji AO et al 2009 [1] | Case-control study | Inpatients with CHD vs healthy controls | 257 vs 142 | 52 (31-71) vs 48 (29-68) | 89.1 vs 99.3 | BNP | 110 (2-4050) vs 70 (1-920) pg/ml | TC, LDLc, HDLc, TG | 190 (89-441) vs 182 (93-325) | 124 (27-391) vs 116 (8-201) | 71 (37-147) vs 73 (37-179) | 124 (18-779) vs 89 (35-505) |
| Bao Y et al 2011 [2] | Cross-sectional study | Patients without HF | 469 | 65.6±12.3 | 56.7 | NT-proBNP | / | LDLc, HDLc, TG | / | 100.5±31 | 40.6±10.0 | 154.2±83.4 |
| Brutsaert EF et al 2016 [3] | Cohort study | Older adults free of DM and CVD | 2359 | 74 (64 - 103) | 33.8 | NT-proBNP | 111.5 (60.6-203.8) pg/ml | HDLc, LDLc, TG | / | 128.3±32.5 | 56.5±14.5 | 131.8±67.3 |
| Chang HR et al 2013 [4] | Cross-sectional study | Outpatients with HF | 49 | 66.7±11.2 | / | NT-proBNP | 495.4±142.9 pg/ml | TC, LDLc, HDLc, TG | 192.8±34.0 | 124.9±29.8 | 45.5±12.7 | 161.1±95.4 |
| Chang HR et al 2014 [5] | Cross-sectional study | Hypertensives free of cardiac and renal diseases | 74 | 64.1±9.5 | 36.5 | NT-proBNP | 222.2±289.3 pg/ml | TC, LDLc, HDLc, TG | 196.0±38.3 | 128.7±36.4 | 47.2±13.6 | 154.8±95.0 |
| Chen SF et al 2016 [6] | Cross-sectional study | Patients aged between 60-90 years in a stable conditio | 599 vs 519 | 76.7±7.5 vs 73.8±8.6 | 0 vs 100 | BNP | 108.0±3.4 vs 81.3±3.6 pg/ml | TC, TG | 171±44 | / | / | 132±86 |

| | | n (females vs males) | | | | | | | | | | |
|---|---|---|---|---|---|---|---|---|---|---|---|---|
| Cushman M et al 2014 [7] | Cohort study | Individuals ≥45 years | 1502 | 64.8 | 49.8 | NT-proBNP | 67 (34-137) pg/ml | LDLc, HDLc, TG | / | 112.8 | 51.3 | 130.3 |
| Daniels LB et al 2011 [8] | Cohort study | Ambulatory older adults | 950 | 76.5±7.9 | 39.1 | NT-proBNP | 291.5 pg/ml | HDLc, LDLc, TG | / | 120.9±32.3 | 57.7 | 108.5 |
| Everett BM et al 2015 [9] | Cohort study | Participants without CVD | 11076 | 66 (61-71) | 63.7 | BNP | 22.3 (19.0-42.7) ng/L | TC, LDLc, HDLc | 186 (170-200) | 109 (95-119) | 50 (42-61) | / |
| Fu S et al 2016 [10] | Cohort study | Residents aged ≥45 years free of DM | 396 | 66 (58-71) | 53 | NT-proBNP | 41.5 (17.4-80.4) pg/ml | LDLc, HDLc, TG | / | 109.3 (91.5-128.3) | 53.6 (44.9-61.5) | 125.9 (97.2-162.9) |
| Goharian TS et al 2017 [11] | Cross-sectional study | Healthy young adults | 616 | 24.2±3.0 | 47.7 | MR-proANP | 47.3 (38.2-60.7) pmol/l | LDLc, HDLc, TG | / | 104±29.1 | 52.3±12.0 | 97 (71-124) |
| Greene SJ et al 2013 [12] | Cohort study | Inpatients with worsening HF and EF ≤ 40% | 1383 | / | / | NT-proBNP | 4766 (2358-9484) pg/ml | TC | 159 (129-197) | / | / | / |
| He WT et al 2016 [13] | Cross-sectional study | Community-dwelling older adults | 680 | 68.8±5.3 | 49.1 | BNP | 29.5 (23.7-36,6) pg/ml | TC, LDLc, HDLc, TG | 189.8±37.2 | 112,7±34.7 | 52.9±14.2 | 128.8 (93.3-183.8) |

| | | | | | | | | | | | | |
|---|---|---|---|---|---|---|---|---|---|---|---|---|
| | | without overt HF | | | | | | | | | | |
| Hong et al 2008 [14] | cohort study | Patients who underwent PCI with normal EF | 348 | 61.5±9.2 | 67.5 | NT-proBNP | 173.1±89.7 pg/ml | TC, LDLc, HDLc, TG | 188.0±88.1 | 124.5±93.6 | 45.7±10.4 | 96.7±56.0 |
| Hsieh et al 2013 [15] | Cross-sectional study | Hypertensives free of cardiac and renal diseases | 148 | 64.11±9.50 | 36.5 | NT-proANP | 2.64±2.35 ng/ml | TC, LDLc, HDLc, TG | 195.96±38.35 | 128.69±36.41 | 47.19±13.60 | 154.79±95.00 |
| Kawase S et al 2015 [16] | Cross-sectional study | Patients with acute ischemic stroke (cardioembolic vs non-cardioembolic) | 241 vs 477 | 77.7±10.2 vs 72.0±11.8 | 57.7 vs 64.2 | BNP | 366.6±516.2 vs 105.6±240.8 | HDLc, TG | / | / | 55.5±16.2 vs 54.1±18.6 | 90.7±44.0 vs 113.0±56.9 |
| Lauria PB et al 2013 [17] | Cross-sectional study | Women with PCOS vs healthy women | 36 vs 40 | 30 (15-43) vs 29 (25-34) | 0 vs 0 | ANP | 120 (83-170) vs 81 (55-108) pg/ml | TG | / | / | / | 71 (58-94) vs 81 (65-108) |
| Lazo M et al 2013 [18] | Cohort study | Adults free of cardiac and renal diseases | 7822 | 62.2±5.4 | 40.9 | NT-proBNP | 61.8 (31.1-114.4) pg/ml | LDLc, HDLc, TG | / | 123.6±32.7 | 51.8±16.3 | / |

| | | | | | | | | | | | | | |
|---|---|---|---|---|---|---|---|---|---|---|---|---|---|
| Lee KM et al 2018 [19] | Cross-sectional study | Kidney transplant patients free of cardiac diseases | 66 | 52.08± 9.90 | 53 | NT-proBNP | 9.76 (4.30-24.33) pg/ml | TC, LDLc, HDLc, TG | 197.58± 47.08 | 108.93± 40.67 | 51.58±16.68 | 108.00 (80.75-167.00) |
| Li J et al 2018 [20] | Cross-sectional study | Patients with acute ischemic stroke | 246 | 64.2±4.8 | 52.8 | NT-proBNP | 625.25± 59.83 pg/ml | TC, LDLc, HDLc, TG | 202±42 | 129±31 | 42±9 | 182±77 |
| Li WY et al 2011 [21] | Cross-sectional study | Population free of HF and renal disease | 540 | 52±11 | 55.9 | NT-proBNP | / | HDLc, TG | / | / | 49.5±21.0 | 149±102 |
| Mansoor A et al 2009 [22] | Cross-sectional study | HIV-infected women | 454 | 41.6±8.7 | 0 | NT-proBNP | 142.44± 524.80 ng/l | TC, HDLc, TG | 174±39 | / | 47±17 | 139±93 |
| Mizuno Y et al 2013 [23] | Cross-sectional study | Patients who underwent cardiac catheterization | 62 | 62.5±11.7 | 61.3 | BNP | 57.9 (20.2-167.4) pg/ml | TG | / | / | / | 147.6 (98.7-225.7) |
| Murphy CA et al 2018 [24] | Cross-sectional study | HIV-infected adults vs Non-HIV-infected adults free of CVD | 20 vs 10 | / | / | BNP | 60 (44-152) vs 196 (91-251) pg/ml | TC | / | / | / | / |

| | | | | | | | | | | | | |
|---|---|---|---|---|---|---|---|---|---|---|---|---|
| Nakatsuji H et al 2012 [25] | Cross-sectional study | Male employees | 500 | 55±9 | 100 | BNP | 10.8±13.0 pg/ml | LDLc, HDLc, TG | / | 122±28 | 56±13 | 140±95 |
| Nayak BS et al 2015 [26] | Cross-sectional study | High CV risk Africans vs East Indians free of cardiac and renal diseases | 78 vs 82 | 61.8±8.1 vs 59.3±8.2 | 47.4 vs 43.9 | NT-proBNP | 116.8±45.8 vs 144.3±93.4 pg/ml | LDLc, HDLc, TG | / | 142.5±34.7 vs 139.1±46.9 | 45.5±9.6 vs 43.7±11.9 | 141.2±46.8 vs 169.0±65.0 |
| Olalla J et al 2015 [27] | Cross-sectional study | HIV patients on active antiretroviral therapy | 146 | 50.14 (48.79-51.50) | 76 | NT-proBNP | 77.7 pg/ml | TG | / | / | / | 167.28 (149.43-185.12) |
| Olsen et al 2005 [28] | Cohort study | Untreated patients free of CVD | 2070 | / | / | NT-proBNP | / | TC, LDLc, HDLc, TG | / | / | / | / |
| Oztekin S et al 2011 [29] | Cross-sectional study | Patients with MetS free of cardiac and renal diseases | 192 | 54.32±8.48 | 22.9 | BNP | 7.73 (0.01-99) pg/ml | TG | / | / | / | 224.33 (40-1729) |
| Price AH et al 2014 [30] | Cohort study | Patients with DM aged between 60 and 75 years | 1066 | 67.9±4.2 | 51.3 | NT-proBNP | 75 (37, 169) pg/ml | TC, HDLc | 166±35 | / | 50±15 | / |

| | | | | | | | | | | | | |
|---|---|---|---|---|---|---|---|---|---|---|---|---|
| Ribeiro A et al 2015 [31] | Cohort study | Acute HF patients | 496 | 78 (72-84) | 42.3 | BNP | 1637.8 (915.0-2762.8) pg/ml | TC | 150 (126-186) | / | / | / |
| Ricci MA et al 2017 [32] | Cross-sectional study | Obese patients free of cardiac and renal diseases | 154 | 43.4±11 | 33.8 | BNP | 13.0 (6.7-21.0) pg/ml | HDLc, TG | / | / | 46.0 (40.0-56.0) | 131.5 (89.7-178.2) |
| Sanchez OA et al 2014 [33] | Cohort study | Asymptomatic adults free of overt CVD | 5597 | 62.9±10.3 | 48.5 | NT-proBNP | / | TC, LDLc, HDLc, TG | 194.3±36 | 117±31.5 | 50.8±15 | 133.7±90.3 |
| Sezen Y. et al 2009 [34] | Case-control study | 39 patients with MetS and 59 sex- and age-matched controls | 98 | 49.8±47.4 | 49.0 | NT-proBNP | 68.9 (median) pg/ml | LDLc, HDLc, TG | / | 113.8±33.4 | 43.4±9.6 | 174.4±84.9 |
| Shivananda Nayak B et al 2013 [35] | Cross-sectional study | Diabetic vs non-diabetic elderly free of heart, kidney and liver diseases | 51 vs 65 | 61.6± 9.0 vs 62.0± 7.6 | 33.33 vs 30.77 | NT-proBNP | 125.5± 49.7 vs 64.3 ± 34.6 pg/ml | HDLc, TG | / | / | 48.8 ± 16.8 vs 52.6 ± 14.4 | 181.4 ± 75.6 vs 107.8 ± 46.5 |

| | | | | | | | | | | | | |
|---|---|---|---|---|---|---|---|---|---|---|---|---|
| Siervo M et al 2010 [36] | Cross-sectional study | Healthy volunteers | 1000 | 47.6 ± 17.5 | 44.1 | NT-proBNP | 41.0 (20.1-74.8) pg/ml | TC, HDLc, TG | 207.8 ± 41.0 | / | 62.5 ± 17.3 | 113 (81-163) |
| Spannella F et al 2018 [37] | Cross-sectional study | Hospitalized elderly free of medical conditions or medications with potential interference | 288 | 87.7 ± 6.2 | 57.3 | NT-proBNP | 2949 (1005-7335) pg/ml | TC, LDLc, HDLc, TG | 145.1 ± 40.3 | 84.0 ± 29.5 | 38.4 ± 18.6 | 100 (75-129) |
| Takeuchi H et al 2012 [38] | Cross-sectional study | Inpatients and outpatients with active cardiac conditions | 46 | 57±17 | 39 | BNP | 71±159 pg/ml | TC, LDLc, HDLc, TG | 196±38 | 109±31 | 72±25 | 122±88 |
| Tanaka A et al 2017 [39] | Cohort study | General working population | 2140 | 49.9±8.2 | 62.4 | NT-proBNP | 26.7 (14.6-48.6) | TC, LDLc, HDLc, TG | 205.2±34.1 | 125.7±32.4 | 65.4±17.0 | 83 (60-123) |
| Theilade S et al 2015 [40] | Cohort study | Patients with type 1 DM without ESRD | 667 | 55±13 | 56 | MR-proANP | 74.7 (49.2-116.8) pmol/L | TC | 182±35 | / | / | / |

| | | | | | | | | | | | | |
|---|---|---|---|---|---|---|---|---|---|---|---|---|
| Then C et al 2013 [41] | Cohort study | Subjects participating in the KORA F4 study | 1272 | 56.6±12.7 | 49 | MR-proANP | / | LDLc, HDLc, TG | / | 136±34 | 57±14 | 104 (72-151) |
| Tsai JP et al 2016 [42] | Cross-sectional study | Peritoneal dialysis patients | 52 | 52.85±13.16 | 37.3 | NT-proBNP | 88.12±63.72 ng/ml | TC, HDLc, TG | 194.60±51.62 | / | 43.96±13.73 | 209.06±134.13 |
| Wang JH et al 2014 [43] | Cross-sectional study | Older subjects free of cardiac and renal diseases | 84 | 73.31±4.66 | 33.3 | NT-proBNP | 251.26±282.69 pg/ml | TC, LDLc, HDLc, TG | 192.60±37.74 | 125.58±35.26 | 49.69±13.34 | 142.30±82.39 |
| Wang TJ et al 2007 [44] | Cohort study | Females vs males by Framingham Offspring Study free of HF and CKD | 1784 vs<br>1549 | 58±10 vs<br>58±10 | 0 vs<br>100 | BNP | 16.4±20.8 vs 14.2±20.7 pg/mL | TC, HDLc, TG | 212±38<br>199±41 | / | 58±16<br>43±12 | 133±83<br>148±167 |
| Yuan J et al 2016 [45] | Cross-sectional study | Patients with active SLE | 46 | 41.7 (24-66) | 13 | BNP | 643.1±98.4 pg/ml | TC, LDLc, HDLc, TG | 209±15 | 109±10 | 41±3 | 389±49 |
| Zhu WH et al 2016 [46] | Cross-sectional study | Adults free of cardiac/renal diseases and CV | 11508 | 44.56±0.15 | 54 | NT-proBNP | 18.6±0.3 pg/ml | TC, LDLc, HDLc, TG | 183.9±0.5 | 102.6±0.0 | 49.1±1.5 | 122.9±1.5 |

| | | |
|---|---|---|
| | medications | |

Normal continuous variables were expressed as mean ± SD. Skewed variables were expressed as median and interquartile range. / indicates data not available. NP: natriuretic peptide; NT-proBNP: n-terminal pro b-type natriuretic peptide; ANP: atrial natriuretic peptide; TC: total cholesterol; LDLc: low density lipoprotein cholesterol; HDLc: high density lipoprotein cholesterol; TG: triglycerides; HF: chronic heart failure; DM: diabetes mellitus; CVD: cardiovascular disease; CHD: coronary heart disease; EF: ejection fraction; PCI: percutaneous coronary intervention; PCOS: polycystic ovary syndrome; HIV: human immunodeficiency virus; MetS: metabolic syndrome; ESRD: end-stage renal disease; CKD: chronic kidney disease; SLE: systemic lupus erythematosus

## REFERENCES


1 Akanji, A. O., Suresh, C. G., Al-Radwan, R. & Fatania, H. R. Body mass and atherogenic dyslipidemia as major determinants of blood levels of B-type natriuretic peptides in Arab subjects with acute coronary syndromes. *Metabolic syndrome and related disorders* **7**, 563-569, doi:10.1089/met.2009.0034 (2009).

2 Bao, Y. *et al.* Relationship between N-terminal pro-B-type natriuretic peptide levels and metabolic syndrome. *Archives of medical science : AMS* **7**, 247-256, doi:10.5114/aoms.2011.22075 (2011).

3 Brutsaert, E. F. *et al.* Longitudinal assessment of N-terminal pro-B-type natriuretic peptide and risk of diabetes in older adults: The cardiovascular health study. *Metabolism: clinical and experimental* **65**, 1489-1497, doi:10.1016/j.metabol.2016.06.002 (2016).

4 Chang, H. R. *et al.* Inverse association of N-terminal pro-B-type natriuretic peptide with metabolic syndrome in patients with congestive heart failure. *PloS one* **8**, e79096, doi:10.1371/journal.pone.0079096 (2013).

5 Chang, H. R. *et al.* N-terminal pro-B-type natriuretic peptide is inversely associated with metabolic syndrome in hypertensive patients. *The American journal of the medical sciences* **348**, 210-214, doi:10.1097/MAJ.0000000000000234 (2014).

6 Chen, S. F. *et al.* Impact of Protein Nutritional Status on Plasma BNP in Elderly Patients. *The journal of nutrition, health & aging* **20**, 937-943, doi:10.1007/s12603-016-0716-z (2016).

7 Cushman, M. *et al.* N-terminal pro-B-type natriuretic peptide and stroke risk: the reasons for geographic and racial differences in stroke cohort. *Stroke* **45**, 1646-1650, doi:10.1161/STROKEAHA.114.004712 (2014).

8 Daniels, L. B. *et al.* Elevated natriuretic peptide levels and cognitive function in community-dwelling older adults. *The American journal of medicine* **124**, 670 e671-678, doi:10.1016/j.amjmed.2011.02.027 (2011).

9 Everett, B. M., Zeller, T., Glynn, R. J., Ridker, P. M. & Blankenberg, S. High-sensitivity cardiac troponin I and B-type natriuretic Peptide as predictors of vascular events in primary prevention: impact of statin therapy. *Circulation* **131**, 1851-1860, doi:10.1161/CIRCULATIONAHA.114.014522 (2015).

10 Fu, S., Ping, P., Luo, L. & Ye, P. Deep analyses of the associations of a series of biomarkers with insulin resistance, metabolic syndrome, and diabetes risk in nondiabetic middle-aged and elderly individuals: results from a Chinese community-based study. *Clinical interventions in aging* **11**, 1531-1538, doi:10.2147/CIA.S109583 (2016).

11 Goharian, T. S. *et al.* Associations of Proatrial Natriuretic Peptide with Components of the Metabolic Syndrome in Adolescents and Young Adults from the General Population. *American journal of hypertension* **30**, 561-568, doi:10.1093/ajh/hpx026 (2017).

12 Greene, S. J. *et al.* Prognostic significance of serum total cholesterol and triglyceride levels in patients hospitalized for heart failure with reduced ejection fraction (from the EVEREST Trial). *The American journal of cardiology* **111**, 574-581, doi:10.1016/j.amjcard.2012.10.042 (2013).

13 He, W. T., Mori, M., Yu, X. F. & Kanda, T. Higher BNP levels within physiological range correlate with beneficial nonfasting lipid profiles in the elderly: a cross-sectional study. *Lipids in health and disease* **15**, 3, doi:10.1186/s12944-015-0168-1 (2016).

14 Hong, S. N. *et al.* N-terminal pro-B-type natriuretic peptide level is depressed in patients with significant coronary artery disease who have high body mass index. *International heart journal* **49**, 403-412 (2008).

15 Hsieh, J. C. *et al.* Low serum long-acting natriuretic peptide level correlates with metabolic syndrome in hypertensive patients: a cross-sectional study. *Archives of medical research* **44**, 215-220, doi:10.1016/j.arcmed.2013.03.001 (2013).

16 Kawase, S. *et al.* Plasma Brain Natriuretic Peptide is a Marker of Prognostic Functional Outcome in Non-Cardioembolic Infarction. *Journal of stroke and cerebrovascular diseases : the official journal of National Stroke Association* **24**, 2285-2290, doi:10.1016/j.jstrokecerebrovasdis.2015.06.006 (2015).

17 Lauria, P. B., Del Puerto, H. L., Reis, A. M., Candido, A. L. & Reis, F. M. Low plasma atrial natriuretic peptide: a new piece in the puzzle of polycystic ovary syndrome. *The Journal of clinical endocrinology and metabolism* **98**, 4882-4889, doi:10.1210/jc.2013-2141 (2013).

18 Lazo, M. *et al.* NH2-terminal pro-brain natriuretic peptide and risk of diabetes. *Diabetes* **62**, 3189-3193, doi:10.2337/db13-0478 (2013).

19 Lee, K. M., Lee, M. C., Lee, C. J., Chen, Y. C. & Hsu, B. G. Inverse Association of N-terminal ProB-type Natriuretic Peptide Level With Metabolic Syndrome in Kidney Transplant Patients. *Transplantation proceedings* **50**, 2496-2501, doi:10.1016/j.transproceed.2018.04.005 (2018).

20 Li, J. *et al.* Effects of serum N-terminal pro B-type natriuretic peptide and D-dimer levels on patients with acute ischemic stroke. *Pakistan journal of medical sciences* **34**, 994-998, doi:10.12669/pjms.344.15432 (2018).

21 Li, W. Y., Chiu, F. C., Chien, Y. F., Lin, J. W. & Hwang, J. J. Association of amino-terminal pro-brain natriuretic peptide with metabolic syndrome. *Internal medicine* **50**, 1143-1147 (2011).

22 Mansoor, A. *et al.* Elevated NT-pro-BNP levels are associated with comorbidities among HIV-infected women. *AIDS research and human retroviruses* **25**, 997-1004, doi:10.1089/aid.2009.0038 (2009).

23 Mizuno, Y. *et al.* Cardiac production of B-type natriuretic peptide is inversely related to the plasma level of free fatty acids in obese individuals - possible involvement of the insulin resistance. *Endocrine journal* **60**, 87-95 (2013).

24 Murphy, C. A. *et al.* Excessive Adiposity and Metabolic Dysfunction Relate to Reduced Natriuretic Peptide During RAAS Activation in HIV. *The Journal of clinical endocrinology and metabolism* **103**, 1558-1565, doi:10.1210/jc.2017-02198 (2018).

25 Nakatsuji, H., Kishida, K., Funahashi, T., Nakagawa, T. & Shimomura, I. Hyperinsulinemia correlates with low levels of plasma B-type natriuretic peptide in Japanese men irrespective of fat distribution. *Cardiovascular diabetology* **11**, 22, doi:10.1186/1475-2840-11-22 (2012).

26 Nayak, B. S. *et al.* Evaluation of N-terminal pro-B-type natriuretic peptide and high-sensitivity C-reactive protein relationship with features of metabolic syndrome in high-risk subgroups for cardiovascular disease. *International journal of applied & basic medical research* **5**, 190-194, doi:10.4103/2229-516X.165369 (2015).

27 Olalla, J. *et al.* Factors related to NT-proBNP levels in HIV patients aged over 40 years. *AIDS research and therapy* **12**, 17, doi:10.1186/s12981-015-0058-7 (2015).

28 Olsen, M. H. *et al.* N-terminal pro brain natriuretic peptide is inversely related to metabolic cardiovascular risk factors and the metabolic syndrome. *Hypertension* **46**, 660-666, doi:10.1161/01.HYP.0000179575.13739.72 (2005).

29 Oztekin, S., Karakurt, O., Yazihan, N. & Unal, I. Relationship of brain natriuretic peptide with metabolic syndrome parameters: an observational study. *Anadolu kardiyoloji dergisi : AKD = the Anatolian journal of cardiology* **11**, 678-684, doi:10.5152/akd.2011.188 (2011).

30 Price, A. H. *et al.* N-terminal pro-brain natriuretic peptide and risk of cardiovascular events in older patients with type 2 diabetes: the Edinburgh Type 2 Diabetes Study. *Diabetologia* **57**, 2505-2512, doi:10.1007/s00125-014-3375-9 (2014).

31 Ribeiro, A. *et al.* Predictors of natriuretic peptide non-response in patients hospitalized with acute heart failure. *The American journal of cardiology* **115**, 69-74, doi:10.1016/j.amjcard.2014.09.053 (2015).

32 Ricci, M. A. *et al.* Determinants of low levels of brain natriuretic peptide in morbid obesity. *Clinical nutrition* **36**, 1075-1081, doi:10.1016/j.clnu.2016.06.024 (2017).

33 Sanchez, O. A. *et al.* The associations between metabolic variables and NT-proBNP are blunted at pathological ranges: the Multi-Ethnic Study of Atherosclerosis. *Metabolism: clinical and experimental* **63**, 475-483, doi:10.1016/j.metabol.2013.11.017 (2014).

34 Sezen, Y. *et al.* N-terminal pro-brain natriuretic peptide in cases with metabolic syndrome and its relationship with components of metabolic syndrome and left ventricular mass index. *Clinical biochemistry* **42**, 1500-1503, doi:10.1016/j.clinbiochem.2009.07.007 (2009).

35 Shivananda Nayak, B., Teelucksingh, S., Jagessar, A., Maharaj, S. & Maharaj, N. A cross sectional study comparing traditional risk factors with N-terminal pro-BNP in high risk groups for cardiovascular disease in Trinidad, West Indies. *Diabetes & metabolic syndrome* **7**, 8-11, doi:10.1016/j.dsx.2013.02.021 (2013).

36 Siervo, M. *et al.* Angiogenesis and biomarkers of cardiovascular risk in adults with metabolic syndrome. *Journal of internal medicine* **268**, 338-347, doi:10.1111/j.1365-2796.2010.02255.x (2010).

37 Spannella, F. *et al.* N-terminal pro B-Type natriuretic peptide is inversely correlated with low density lipoprotein cholesterol in the very elderly. *Nutrition, metabolism, and cardiovascular diseases : NMCD* **28**, 629-635, doi:10.1016/j.numecd.2018.02.013 (2018).

38 Takeuchi, H. & Sata, M. The relationship among brain natriuretic peptide (BNP), cholesterol and lipoprotein. *Heart Asia* **4**, 11-15, doi:10.1136/heartasia-2011-010042 (2012).

39 Tanaka, A. *et al.* N-terminal pro-brain natriuretic peptide and associated factors in the general working population: a baseline survey of the Uranosaki cohort study. *Scientific reports* **7**, 5810, doi:10.1038/s41598-017-06090-6 (2017).

40 Theilade, S., Hansen, T. W., Goetze, J. P. & Rossing, P. Increased plasma concentrations of midregional proatrial natriuretic Peptide is associated with risk of cardiorenal dysfunction in type 1 diabetes. *American journal of hypertension* **28**, 772-779, doi:10.1093/ajh/hpu227 (2015).

41 Then, C. *et al.* Plasma MR-proANP levels are associated with carotid intima-media thickness in the general community: the KORA F4 study. *Atherosclerosis* **230**, 235-241, doi:10.1016/j.atherosclerosis.2013.07.047 (2013).

42 Tsai JP, L. C., Wang CH, Lai YH, Lin YL, Hsu BG. Inverse association of long-acting natriuretic peptide with metabolic syndrome in peritoneal dialysis patients. *Int J Clin Exp Pathol* **9** (2016).

43 Wang, J. H., Lee, C. J., Hsieh, J. C., Chen, Y. C. & Hsu, B. G. N-terminal pro-B-type natriuretic peptide level inversely associates with metabolic syndrome in elderly persons. *Diabetology & metabolic syndrome* **6**, 15, doi:10.1186/1758-5996-6-15 (2014).

44 Wang, T. J. *et al.* Association of plasma natriuretic peptide levels with metabolic risk factors in ambulatory individuals. *Circulation* **115**, 1345-1353, doi:10.1161/CIRCULATIONAHA.106.655142 (2007).

45 Yuan, J., Li, L. I., Wang, Z., Song, W. & Zhang, Z. Dyslipidemia in patients with systemic lupus erythematosus: Association with disease activity and B-type natriuretic peptide levels. *Biomedical reports* **4**, 68-72, doi:10.3892/br.2015.544 (2016).

46 Zhu, W. H. *et al.* Correlation between B type natriuretic peptide and metabolic risk factors. *Archives of medical science : AMS* **12**, 334-340, doi:10.5114/aoms.2015.57001 (2016).

Supplemental Table 3. Moderator analysis: Association between cardiac NPs and TC

| | K | N | ES | 95% CI | P | Q | $I^2$ | $p^§$ |
|---|---|---|---|---|---|---|---|---|
| **Study design** | | | | | | | | 0.465 |
| Case-control study | 2 | 399 | 0.01 | -0.19 – 0.21 | 0.900 | 3.81 | 73.76 | |
| Cohort study | 12 | 27480 | -0.11 | -0.15 – -0.07 | <0.001 | 101.97‡ | 89.21 | |
| Cross-sectional study | 18 | 15549 | -0.09 | -0.16 – -0.02 | 0.015 | 116.58‡ | 85.42 | |
| **Age classes (years)** | | | | | | | | 0.024 |
| Age<65 | 18 | 26124 | -0.05 | -0.11 – 0.01 | 0.079 | 220.36‡ | 92.29 | |
| Age≥65 | 10 | 14517 | -0.14 | -0.19 – -0.09 | <0.001 | 53.22‡ | 83.09 | |
| **BMI classes** | | | | | | | | 0.035 |
| Normal weight | 8 | 4050 | -0.17 | -0.26 – -0.08 | <0.001 | 39.27‡ | 82.17 | |
| Overweight/obese | 14 | 23493 | -0.06 | -0.11 – 0.02 | 0.007 | 111.56‡ | 88.35 | |
| **Cardiac NP type** | | | | | | | | 0.253 |
| A-type NP | 3 | 867 | -0.01 | -0.16 – 0.14 | 0.921 | 5.57 | 64.11 | |

| | | | | | | | | |
|---|---|---|---|---|---|---|---|---|
| B-type NP | 29 | 42561 | -0.10 | -0.14 – -0.06 | <0.001 | 312.01‡ | 91.03 | |
| **Special populations** | | | | | | | | 0.835 |
| No | 22 | 41058 | -0.09 | -0.13 – -0.05 | <0.001 | 303.14‡ | 93.07 | |
| Yes | 10 | 2370 | -0.10 | -0.15 – -0.04 | 0.001 | 13.91 | 35.28 | |
| **NT-proBNP assays** | | | | | | | | 0.002 |
| ECLIA | 12 | 13236 | -0.09 | -0.15 – -0.04 | 0.001 | 78.89‡ | 86.06 | |
| EIA | 2 | 11560 | 0.02 | -0.00 – 0.03 | 0.101 | 0.14 | 0.00 | |
| IFA | 1 | 246 | 0.02 | -0.11 – 0.14 | 0.758 | / | / | |
| **BNP assays** | | | | | | | | 0.084 |
| Assays for COOH-terminal fragment | 2 | 3333 | -0.08 | -0.11 – -0.05 | <0.001 | 0.03 | 0.00 | |
| Assays for $NH_2$-terminal fragment | 9 | 13106 | -0.15 | -0.22 – -0.09 | <0.001 | 54.36‡ | 85.28 | |

| | | | | | | | | |
|---|---|---|---|---|---|---|---|---|
| RIA | 2 | 399 | 0.01 | -0.19 – 0.21 | 0.900 | 3.81 | 73.76 | |
| **Study quality** | | | | | | | | 0.403 |
| High quality | 21 | 42006 | -0.10 | -0.14 – -0.05 | <0.001 | 309.12‡ | 93.53 | |
| Low quality | 11 | 1422 | -0.07 | -0.12 – -0.01 | 0.012 | 8.96 | 0 | |

* p < 0.05
† p < 0.01
‡ p < 0.001
§ p for comparison between subgroups
Overweight/obesity were defined as BMI ≥25 kg/$m^2$. NOS score of 5 was chosen as cutoff to indicate high quality studies.
NOS: adapted Newcastle-Ottawa Scale; BMI: body mass index; NP: natriuretic peptide; NT-proBNP: N-terminal pro B-type natriuretic peptide; ECLIA: electrochemiluminescence immunoassay; EIA: enzyme immunoassay; IFA: immunofluorescence assay; RIA: radioimmunoassay.

Supplemental Table 4. Moderator analysis: Association between cardiac NPs and LDLc

| | k | N | ES | 95% CI | P | Q | $I^2$ | $p^§$ |
|---|---|---|---|---|---|---|---|---|
| **Study design** | | | | | | | | 0.815 |
| Case-control study | 3 | 497 | -0.03 | -0.23 – 0.18 | 0.800 | 9.35† | 78.61 | |
| Cohort study | 12 | 29692 | -0.09 | -0.12 – -0.06 | <0.001 | 63.23‡ | 82.60 | |
| Cross-sectional study | 16 | 14640 | -0.08 | -0.15 – -0.01 | 0.031 | 92.11‡ | 83.72 | |
| **Age classes (years)** | | | | | | | | 0.253 |
| Age<65 | 20 | 27927 | -0.07 | -0.10 – -0.04 | <0.001 | 64.38‡ | 70.49 | |
| Age≥65 | 10 | 14832 | -0.11 | -0.18 – -0.05 | 0.001 | 89.14‡ | 89.90 | |
| **BMI classes** | | | | | | | | 0.531 |
| Normal weight | 7 | 3996 | -0.11 | -0.15 – -0.06 | <0.001 | 9.01 | 33.38 | |
| Overweight/obese | 19 | 26615 | -0.09 | -0.13 – -0.04 | <0.001 | 133.77‡ | 86.54 | |
| **Cardiac NP type** | | | | | | | | 0.246 |
| A-type NP | 3 | 2036 | -0.03 | -0.13 – 0.07 | 0.546 | 7.76* | 74.23 | |

| | | | | | | | | |
|---|---|---|---|---|---|---|---|---|
| B-type NP | 28 | 42793 | -0.009 | -0.12 – -0.06 | <0.001 | 159.29‡ | 83.05 | |
| **Special populations** | | | | | | | | 0.075 |
| No | 26 | 44168 | -0.10 | -0.13 – -0.07 | <0.001 | 152.29‡ | 83.58 | |
| Yes | 5 | 661 | 0.02 | -0.11 – 0.14 | 0.776 | 8.19 | 51.18 | |
| **NT-proBNP assays** | | | | | | | | 0.001 |
| ECLIA | 18 | 18632 | -0.12 | -0.16 – -0.08 | <0.001 | 90.39‡ | 81.19 | |
| EIA | 1 | 11508 | -0.10 | -0.12 – -0.08 | <0.001 | / | / | |
| IFA | 1 | 246 | 0.13 | 0.01 – 0.25 | 0.041 | / | / | |
| **BNP assays** | | | | | | | | 0.081 |
| Assays for COOH-terminal fragment | 1 | 500 | -0.14 | -0.23 – -0.06 | 0.001 | / | / | |
| Assay for $NH_2$-terminal fragment | 4 | 11462 | -0.05 | -0.07 – -0.03 | <0.001 | 2.17 | 0.00 | |

| | | | | | | | | |
|---|---|---|---|---|---|---|---|---|
| RIA | 1 | 399 | 0.06 | -0.17 – 0.28 | 0.625 | 4.66* | 78.52 | |
| **Study quality** | | | | | | | | 0.134 |
| High quality | 21 | 43167 | -0.10 | -0.13 – -0.07 | <0.001 | 155.77‡ | 87.16 | |
| Low quality | 10 | 1662 | -0.04 | -0.11 – 0.02 | 0.156 | 11.55 | 22.10 | |

* p < 0.05
† p < 0.01
‡ p < 0.001
§ p for comparison between subgroups
Overweight/obesity were defined as BMI ≥25 $kg/m^2$. NOS score of 5 was chosen as cutoff to indicate high quality studies.
NOS: adapted Newcastle-Ottawa Scale; BMI: body mass index; NP: natriuretic peptide; NT-proBNP: N-terminal pro B-type natriuretic peptide; ECLIA: electrochemiluminescence immunoassay; EIA: enzyme immunoassay; IFA: immunofluorescence assay; RIA: radioimmunoassay.

Supplemental Table 5. Moderator analysis: Association between cardiac NPs and HDLc

| | k | N | ES | 95% CI | p | Q | $I^2$ | $p^§$ |
|---|---|---|---|---|---|---|---|---|
| **Study design** | | | | | | | | 0.865 |
| Case-control study | 3 | 497 | 0.03 | -0.25 – 0.32 | 0.814 | 18.72‡ | 89.31 | |
| Cohort study | 14 | 32021 | 0.07 | 0.03 – 0.11 | 0.002 | 181.59‡ | 92.84 | |
| Cross-sectional study | 24 | 17433 | 0.05 | 0.00 – 0.10 | 0.040 | 110.72‡ | 79.23 | |
| **Age classes (years)** | | | | | | | | 0.767 |
| Age<65 | 29 | 33576 | 0.06 | 0.02 – 0.10 | 0.006 | 257.18‡ | 89.11 | |
| Age≥65 | 12 | 16375 | 0.07 | 0.02 – 0.12 | 0.008 | 74.46‡ | 85.23 | |
| **BMI classes** | | | | | | | | 0.816 |
| Normal weight | 9 | 4525 | 0.08 | 0.01 – 0.15 | 0.024 | 29.32‡ | 72.71 | |
| Overweight/obese | 27 | 32824 | 0.09 | 0.05 – 0.12 | <0.001 | 204.89‡ | 87.31 | |
| **Cardiac NP type** | | | | | | | | 0.001 |
| A-type NP | 4 | 2088 | 0.14 | 0.10 – 0.18 | <0.001 | 1.25 | 0.00 | |

| | | | | | | | | |
|---|---|---|---|---|---|---|---|---|
| B-type NP | 37 | 47863 | 0.05 | 0.02 – 0.09 | 0.001 | 320.02‡ | 88.75 | |
| **Special populations** | | | | | | | | 0.023 |
| No | 333 | 48307 | 0.08 | 0.05 – 0.11 | <0.001 | 272.00‡ | 88.24 | |
| Yes | 8 | 1644 | -0.08 | -0.21 – 0.06 | 0.253 | 41.80‡ | 83.25 | |
| **NT-proBNP assays** | | | | | | | | 0.022 |
| ECLIA | 23 | 19738 | 0.05 | 0.00 – 0.10 | 0.046 | 206.40‡ | 89.34 | |
| EIA | 2 | 11560 | 0.03 | 0.01 – 0.05 | 0.001 | 0.00 | 0.00 | |
| IFA | 1 | 246 | -0.14 | -0.26 – -0.01 | 0.031 | / | / | |
| **BNP assays** | | | | | | | | 0.128 |
| Assays for COOH-terminal fragment | 3 | 3833 | 0.11 | 0.03 – 0.18 | 0.007 | 10.49† | 80.93 | |
| Assays for $NH_2$-terminal fragment | 5 | 11616 | -0.04 | -0.06 – 0.13 | 0.421 | 53.93‡ | 92.58 | |

| | | | | | | | | |
|---|---|---|---|---|---|---|---|---|
| RIA | 2 | 399 | 0.13 | 0.08 – 0.28 | <0.001 | 0.50 | 0.00 | |
| **Study quality** | | | | | | | | 0.216 |
| High quality | 29 | 47783 | 0.07 | 0.04 – 0.11 | <0.001 | 266.15‡ | 89.48 | |
| Low quality | 12 | 2168 | -0.00 | -0.12 – 0.11 | 0.985 | 62.71‡ | 82.46 | |

* $p < 0.05$
† $p < 0.01$
‡ $p < 0.001$
§ p for comparison between subgroups
Overweight/obesity were defined as BMI ≥25 $kg/m^2$. NOS score of 5 was chosen as cutoff to indicate high quality studies.
NOS: adapted Newcastle-Ottawa Scale; BMI: body mass index; NP: natriuretic peptide; NT-proBNP: N-terminal pro B-type natriuretic peptide; ECLIA: electrochemiluminescence immunoassay; EIA: enzyme immunoassay; IFA: immunofluorescence assay; RIA: radioimmunoassay.

Supplemental Table 6. Moderator analysis: Association between cardiac NPs and TG

| | K | N | ES | 95% CI | p | Q | $I^2$ | $p^§$ |
|---|---|---|---|---|---|---|---|---|
| **Study design** | | | | | | | | 0.928 |
| Case-control study | 3 | 497 | -0.12 | -0.23 – -0.01 | 0.030 | 3.00 | 33.23 | |
| Cohort study | 11 | 19877 | -0.12 | -0.23 – -0.02 | 0.019 | 467.75† | 97.86 | |
| Cross-sectional study | 32 | 19275 | -0.11 | -0.15 – -0.06 | <0.001 | 189.52† | 83.64 | |
| **Age classes (years)** | | | | | | | | 0.100 |
| Age<65 | 34 | 34057 | -0.09 | -0.15– -0.03 | 0.005 | 694.33† | 95.25 | |
| Age≥65 | 12 | 5592 | -0.16 | -0.23– -0.09 | <0.001 | 73.43† | 85.02 | |
| **BMI classes** | | | | | | | | 0.266 |
| Normal weight | 13 | 6030 | -0.16 | -0.22– -0.09 | <0.001 | 58.44† | 79.47 | |
| Overweight/obese | 27 | 20955 | -0.10 | -0.18– -0.03 | 0.006 | 550.51† | 95.28 | |
| **Cardiac NP type** | | | | | | | | 0.409 |
| A-type NP | 6 | 2164 | -0.14 | -0.21– -0.08 | <0.001 | 5.67 | 11.82 | |

| | | | | | | | | |
|---|---|---|---|---|---|---|---|---|
| B-type NP | 40 | 37485 | -0.11 | -0.16 – -0.06 | <0.001 | 762.19† | 94.88 | |
| **Special populations** | | | | | | | | 0.088 |
| No | 35 | 37578 | -0.13 | -0.18 – -0.08 | <0.001 | 704.22† | 95.17 | |
| Yes | 11 | 2071 | -0.04 | -0.13 – -0.06 | 0.466 | 38.16† | 73.80 | |
| **NT-proBNP assays** | | | | | | | | <0.001 |
| ECLIA | 22 | 18670 | -0.10 | -0.18 – -0.01 | 0.026 | 561.35† | 96.26 | |
| EIA | 2 | 11560 | -0.07 | -0.09 – -0.05 | <0.001 | 0.59 | 0.00 | |
| CLIA | 1 | 146 | 0.23 | 0.07 – 0.38 | 0.005 | / | / | |
| IFA | 1 | 246 | 0.13 | 0.00 – 0.25 | 0.047 | / | / | |
| **BNP assays** | | | | | | | | 0.079 |
| Assays for COOH-terminal fragment | 4 | 3895 | -0.13 | -0.20 – -0.05 | 0.001 | 11.24* | 73.30 | |

| | | | | | | | | |
|---|---|---|---|---|---|---|---|---|
| Assays for $NH_2$-terminal fragment | 5 | 1658 | -0.23 | -0.29 – -0.17 | <0.001 | 5.76 | 30.55 | |
| RIA | 2 | 399 | -0.17 | -0.26 – -0.07 | 0.001 | 0.01 | 0.00 | |
| **Study quality** | | | | | | | | 0.659 |
| High quality | 30 | 36846 | -0.10 | -0.16– -0.04 | 0.001 | 702.23† | 95.87 | |
| Low quality | 16 | 2816 | -0.12 | -0.20– -0.04 | 0.002 | 56.43† | 73.42 | |

* $p < 0.05$
† $p < 0.001$
§ p for comparison between subgroups
Overweight/obesity were defined as BMI ≥25 $kg/m^2$. NOS score of 5 was chosen as cutoff to indicate high quality studies.
NOS: adapted Newcastle-Ottawa Scale; BMI: body mass index; NP: natriuretic peptide; NT-proBNP: N-terminal pro B-type natriuretic peptide; ECLIA: electrochemiluminescence immunoassay; EIA: enzyme immunoassay; CLIA: chemiluminescence immunoassay; IFA: immunofluorescence assay; RIA: radioimmunoassay.

Supplemental Figure 1, Panel (A). Association between ES and age, regarding total cholesterol.

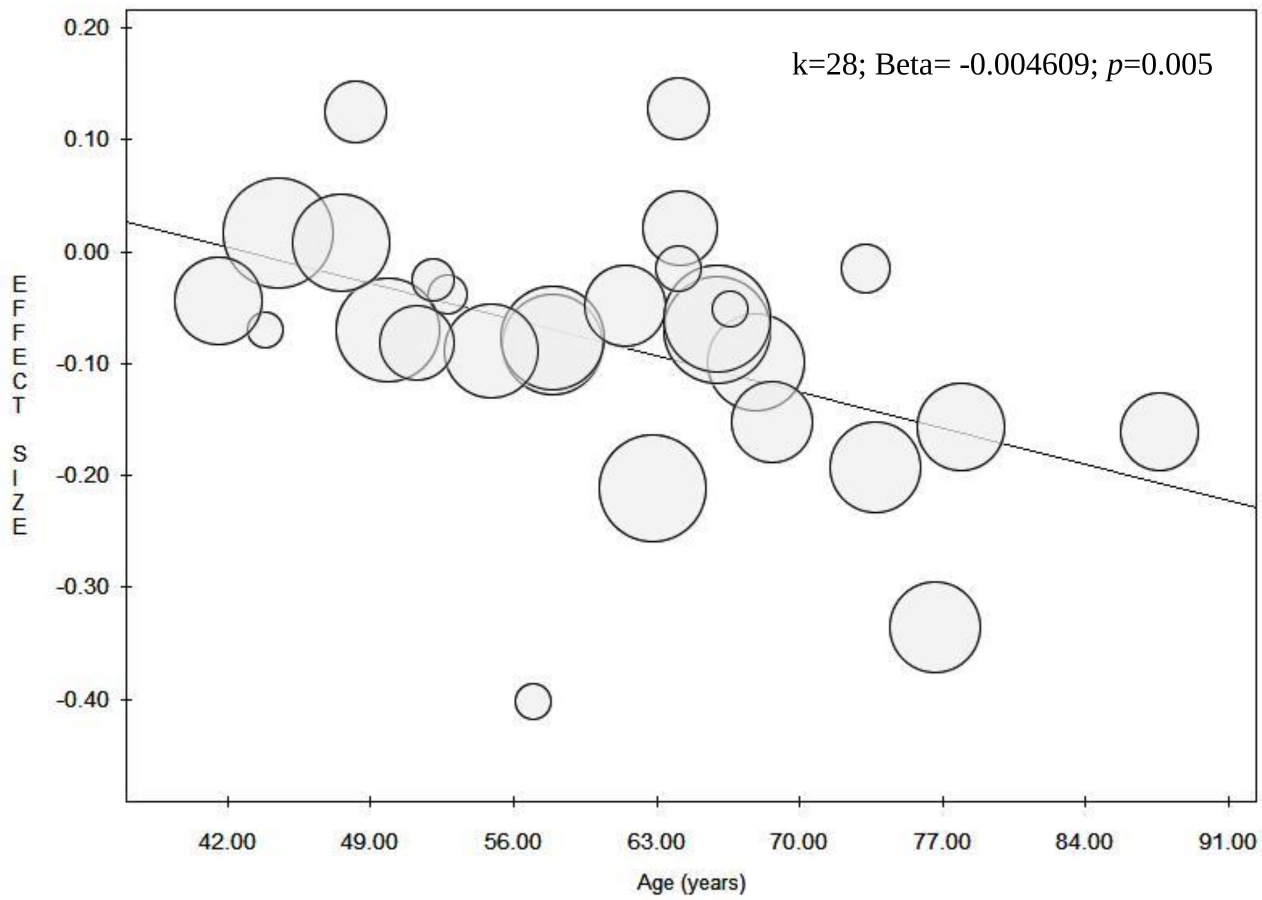

Supplemental Figure 1, Panel (B). Association between ES and body mass index, regarding total cholesterol.

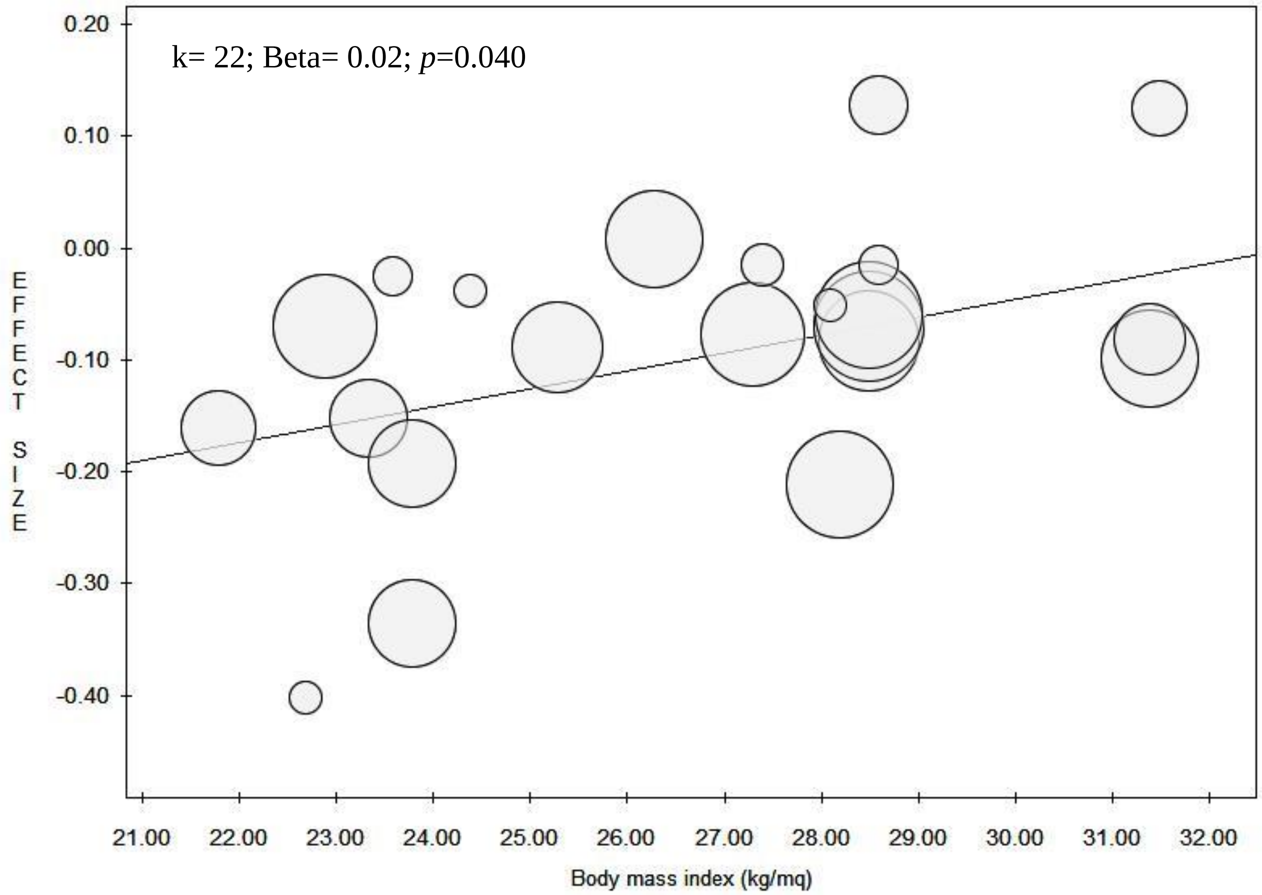

Supplemental Figure 1, Panel (C). Association between ES and NT-proBNP levels, regarding total cholesterol.

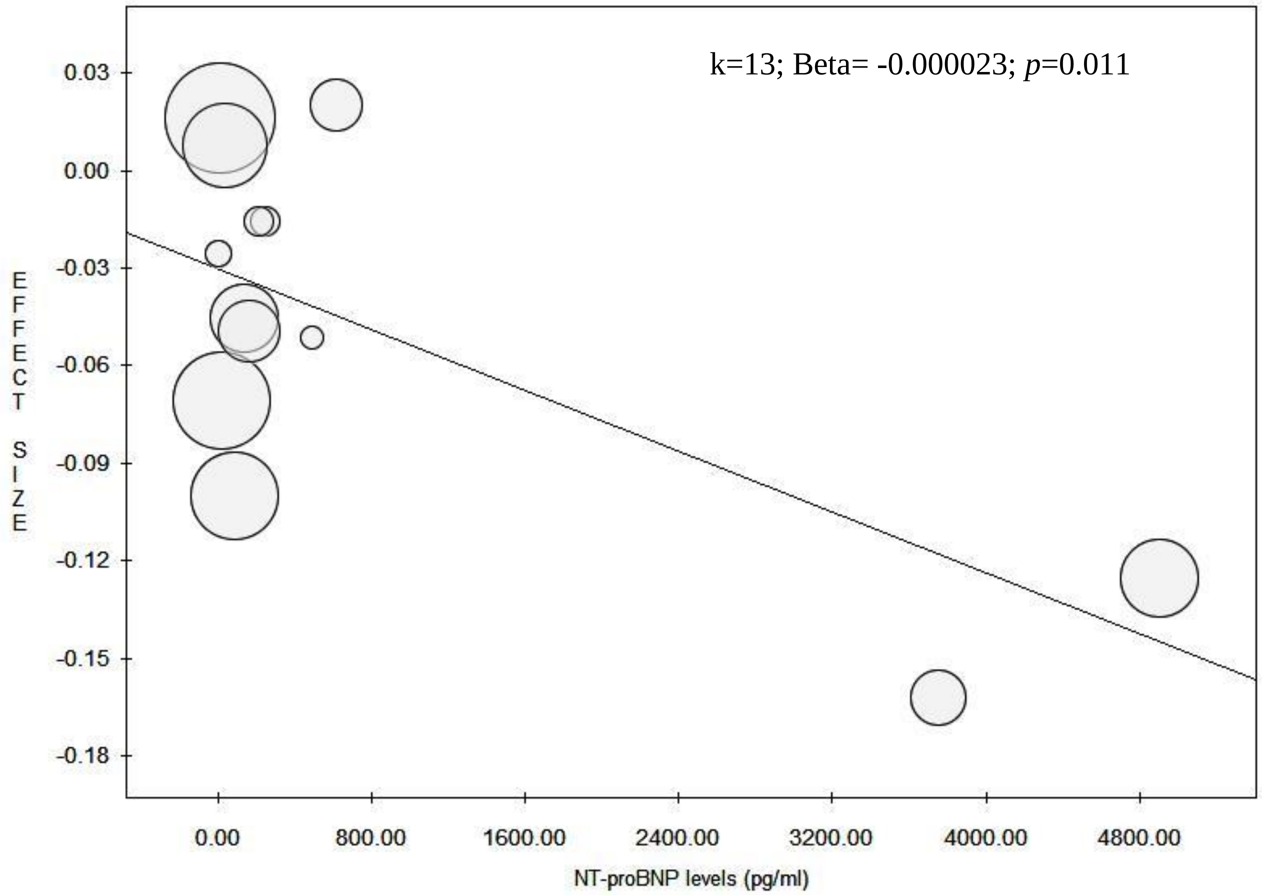

Supplemental Figure 1, Panel (D). Association between ES and prevalence of males, regarding total cholesterol.

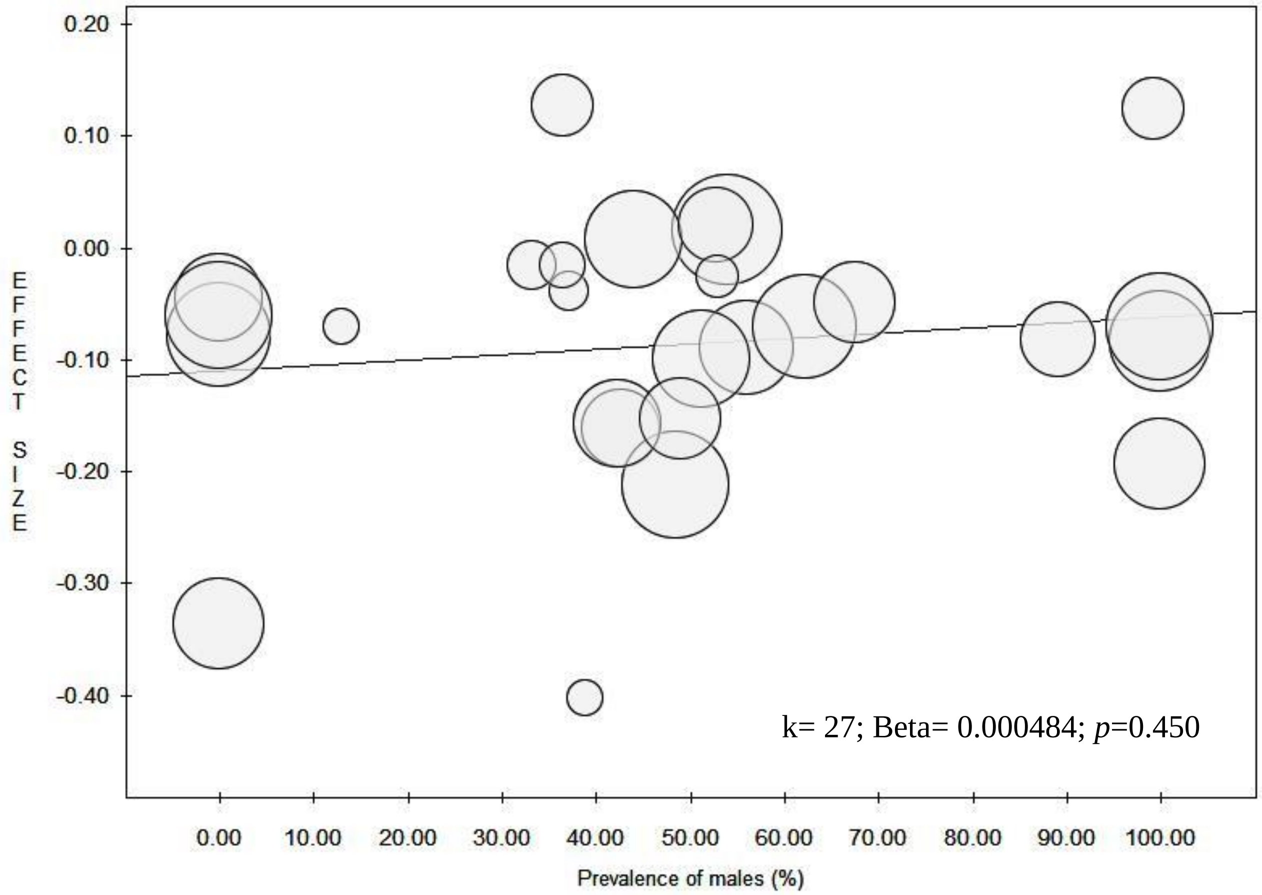

Supplemental Figure 1, Panel (E). Association between ES and estimated glomerular filtration rate, regarding total cholesterol.

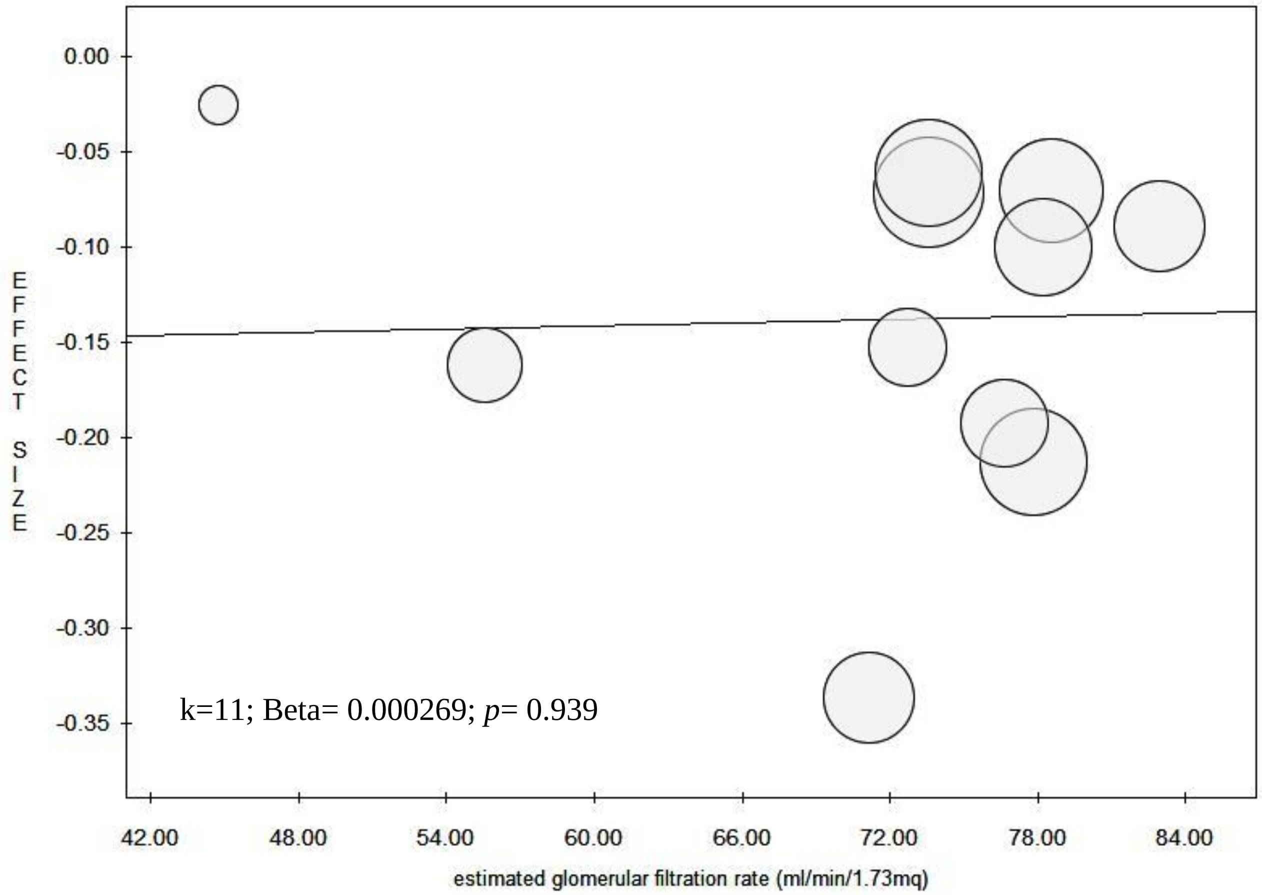

Supplemental Figure 1, Panel (F). Association between ES and prevalence of diabetics, regarding total cholesterol.

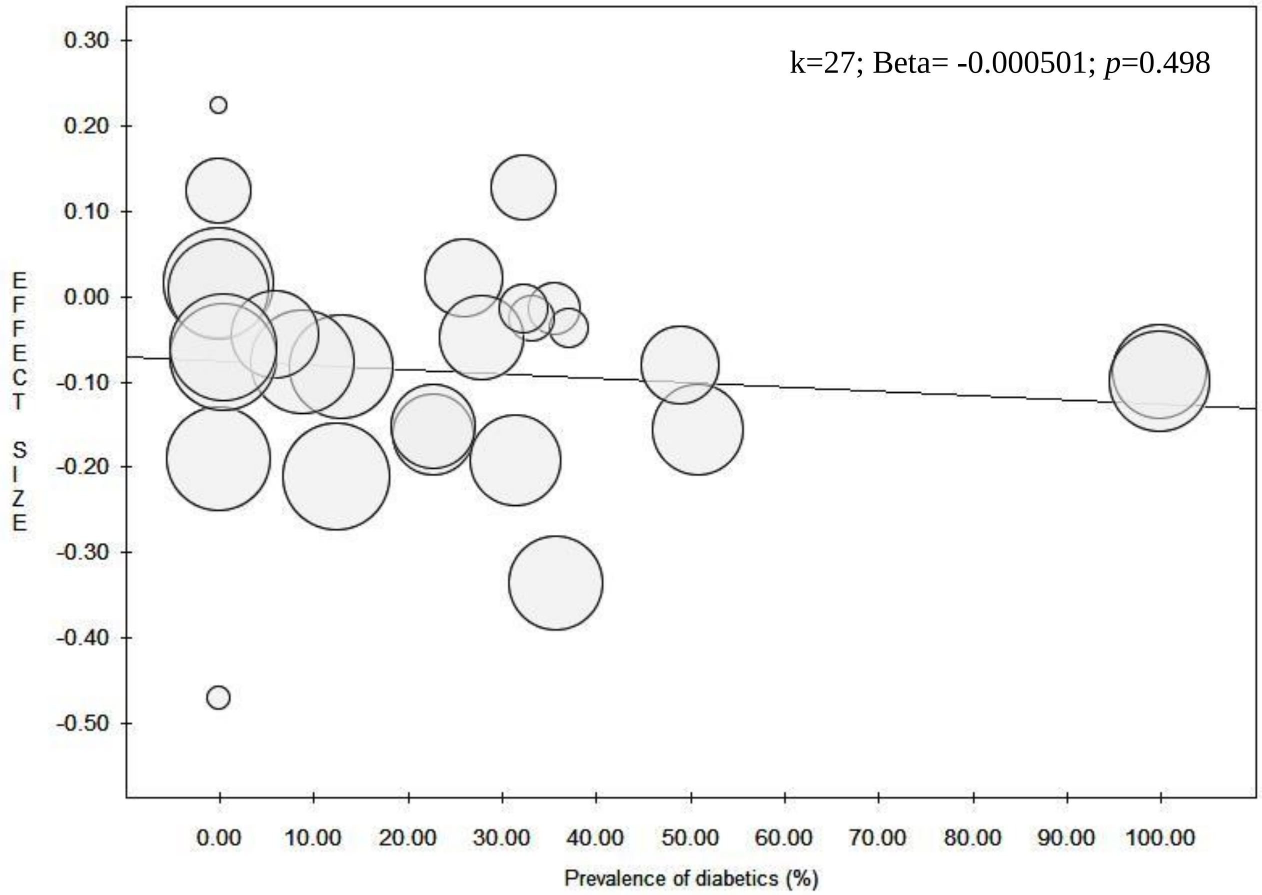

Supplemental Figure 1, Panel (G). Association between ES and prevalence of hypertensives, regarding total cholesterol.

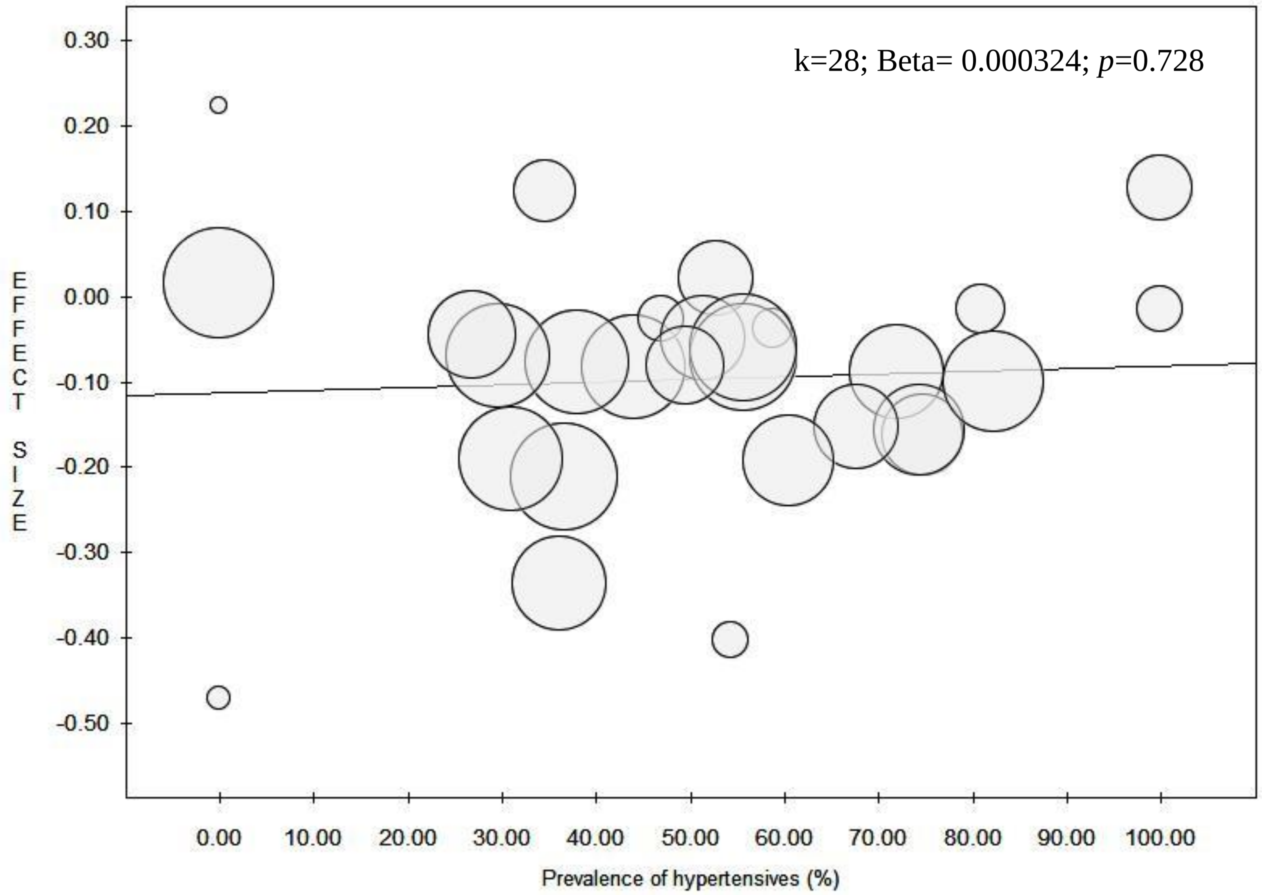

Supplemental Figure 1, Panel (H). Association between ES and prevalence of lipid-lowering therapy, regarding total cholesterol.

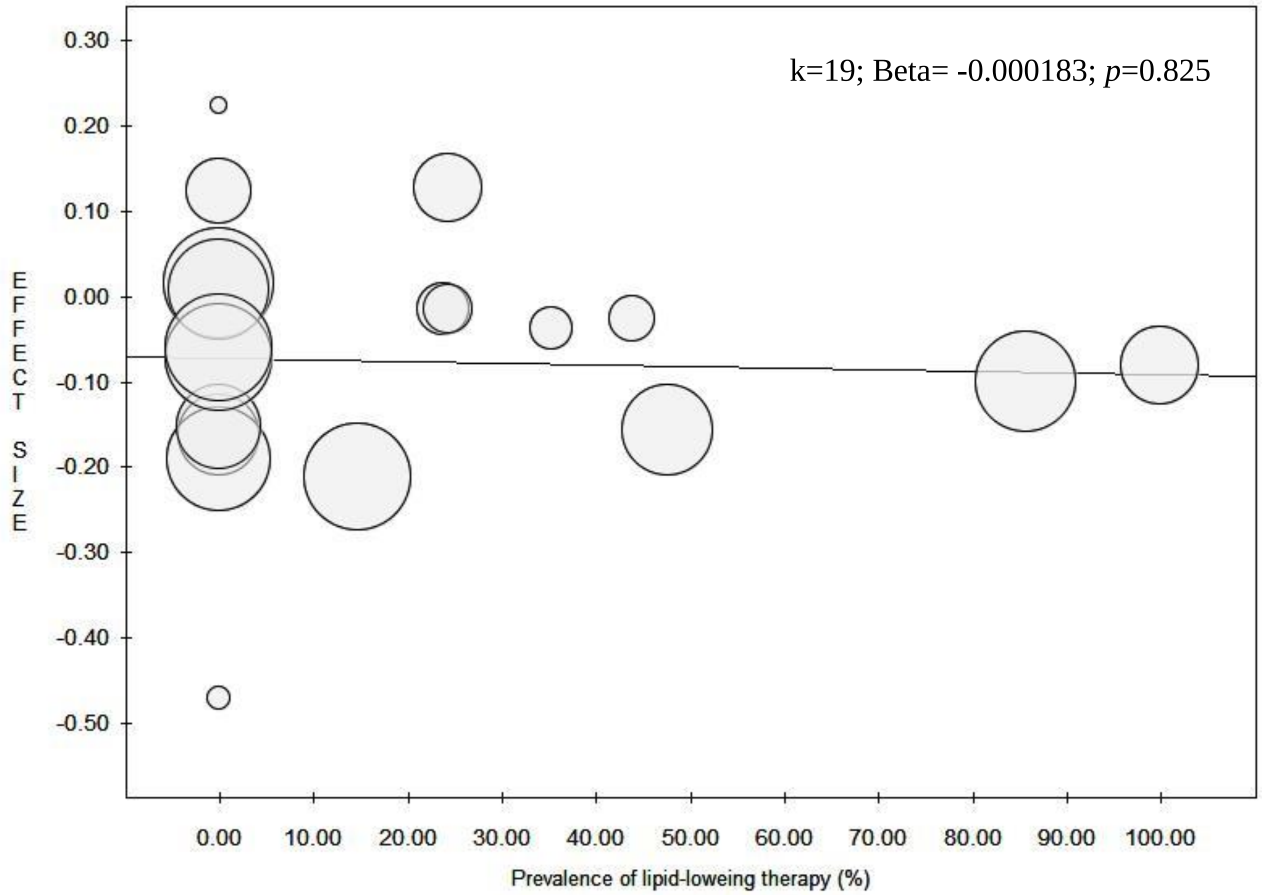

Supplemental Figure 2, Panel (A). Association between ES and age, regarding LDLc.

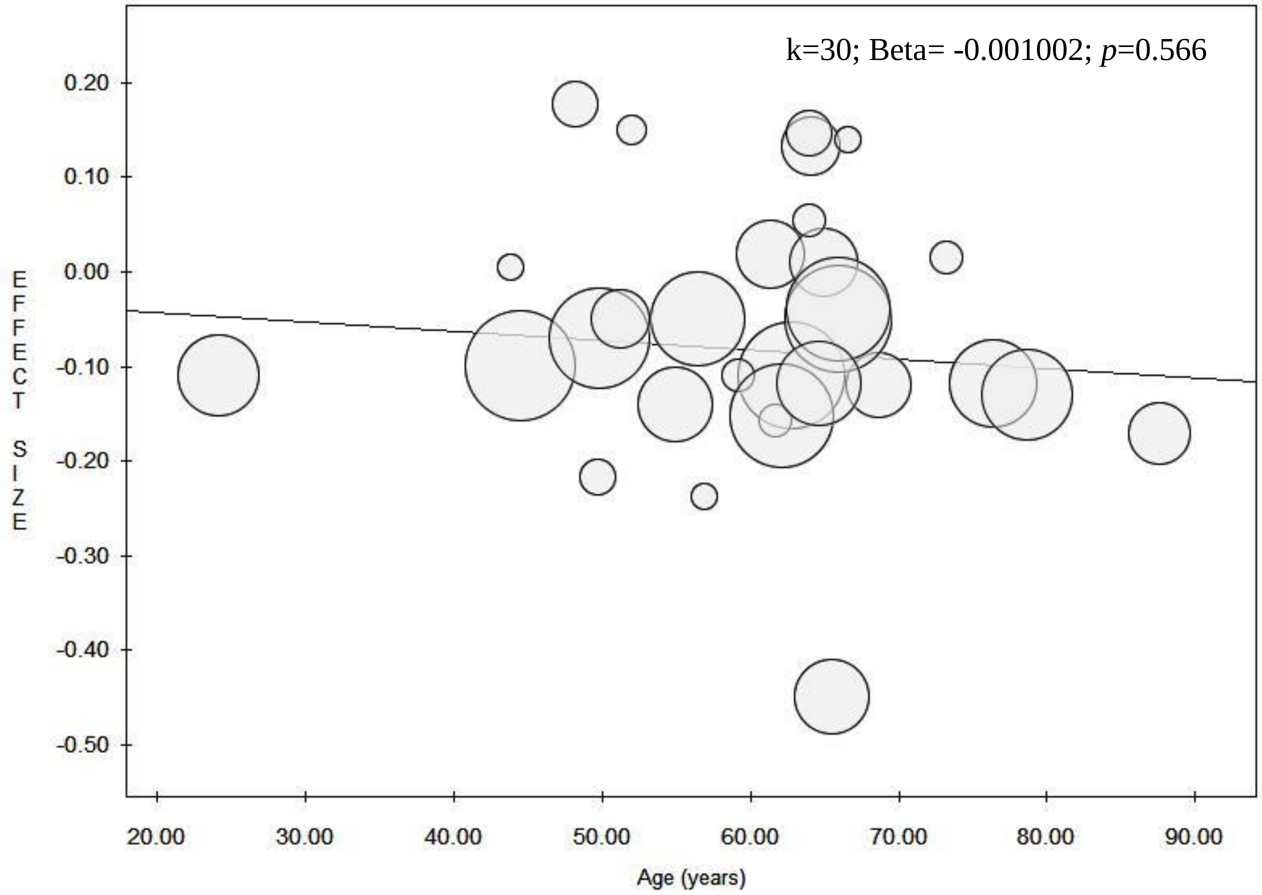

Supplemental Figure 2, Panel (B). Association between ES and body mass index, regarding LDLc.

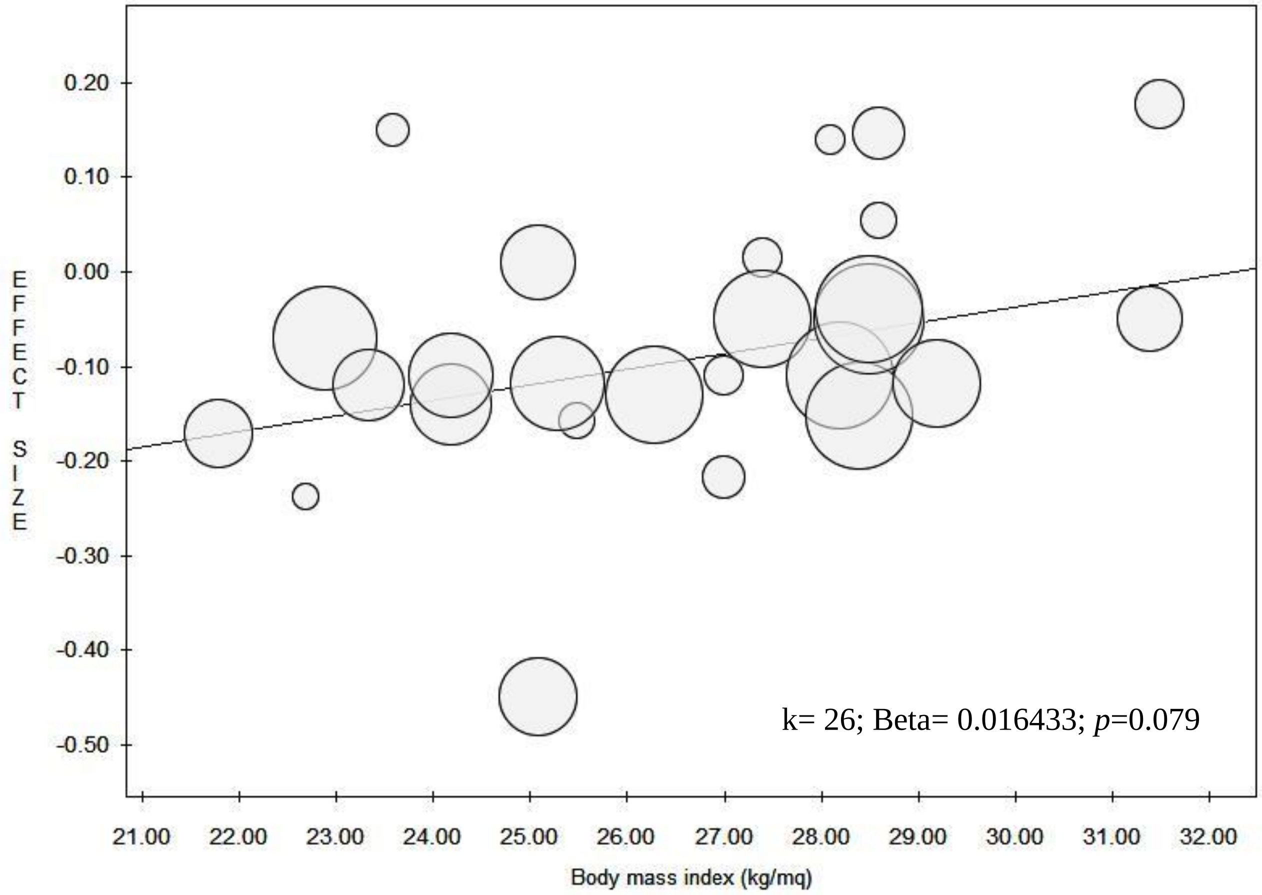

Supplemental Figure 2, Panel (C). Association between ES and NT-proBNP levels, regarding LDLc.

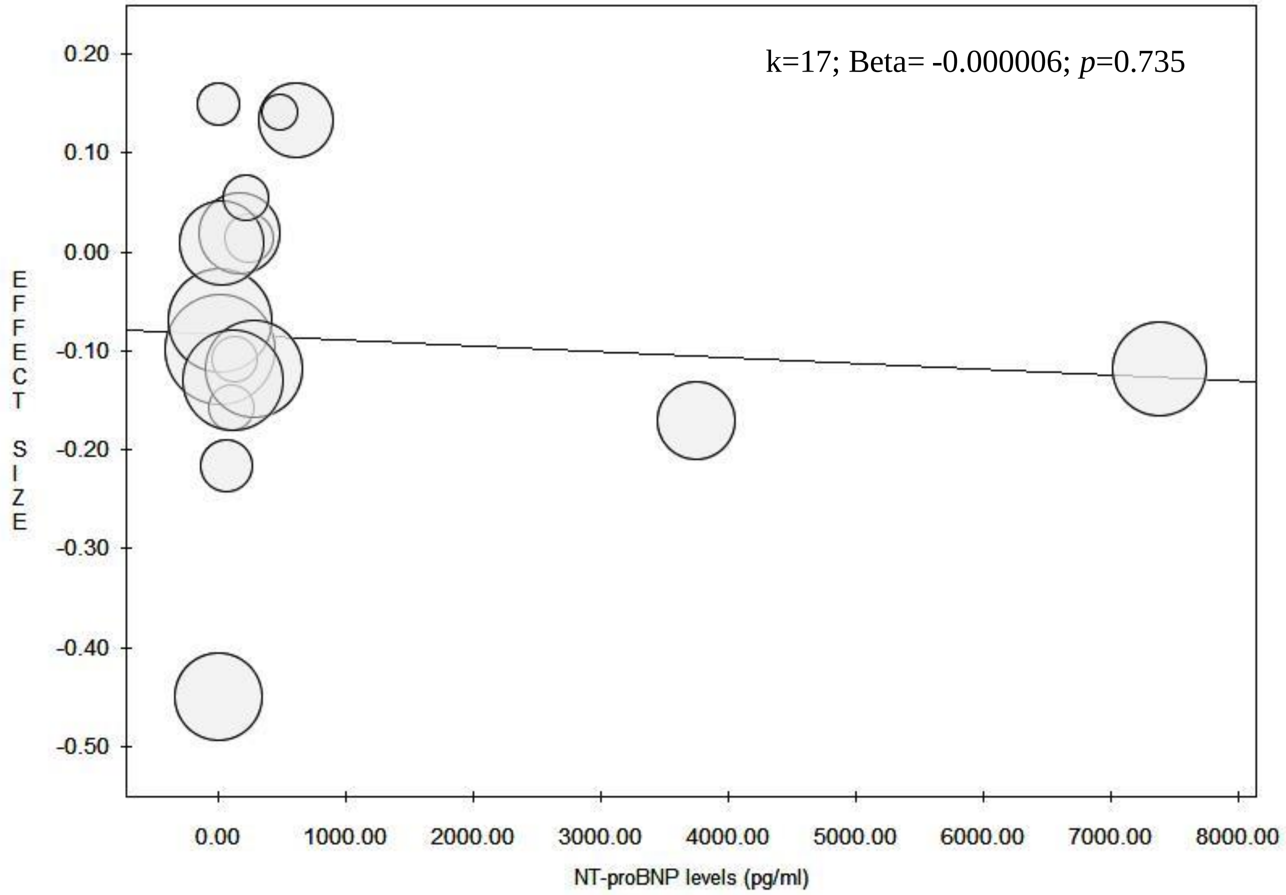

Supplemental Figure 2, Panel (D). Association between ES and prevalence of males, regarding LDLc.

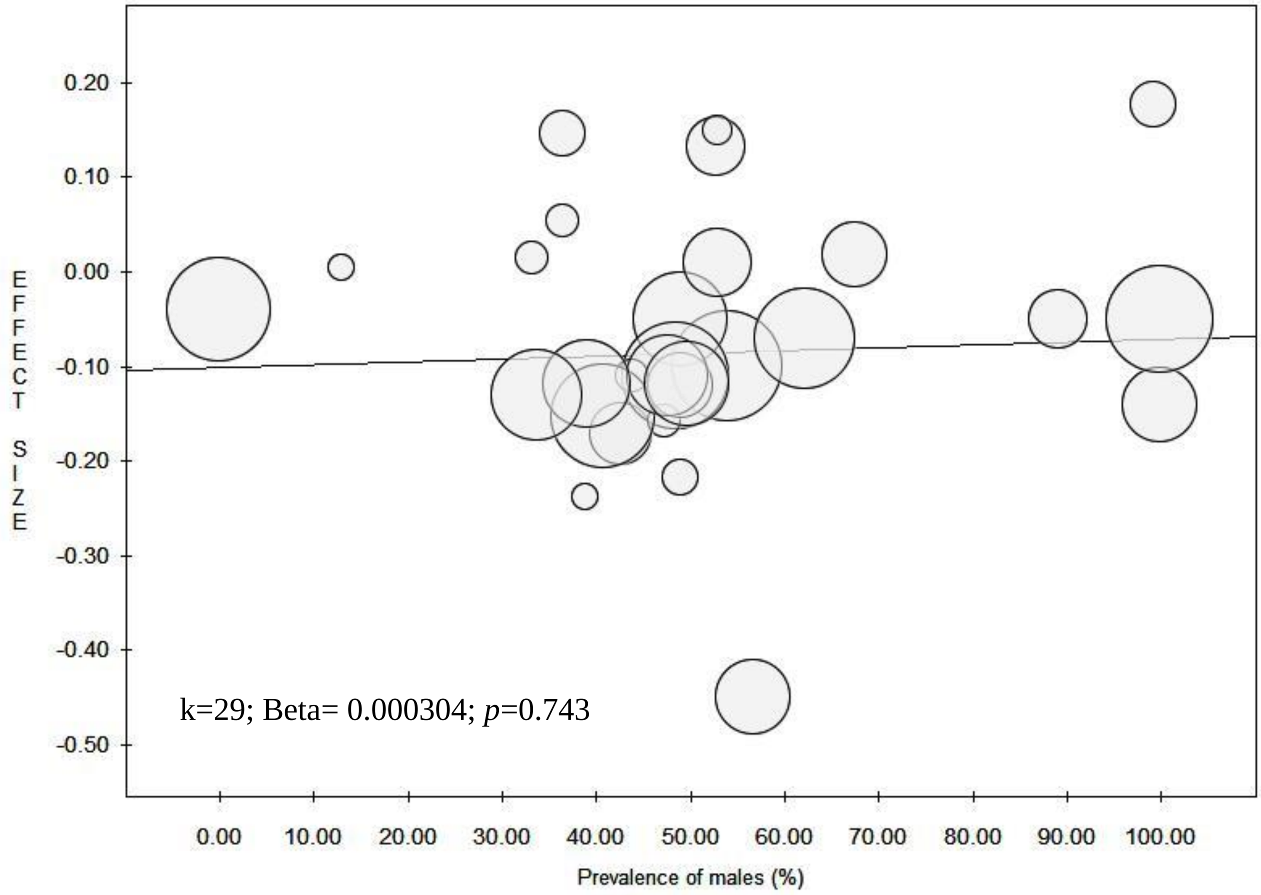

Supplemental Figure 2, Panel (E). Association between ES and estimated glomerular filtration rate, regarding LDLc.

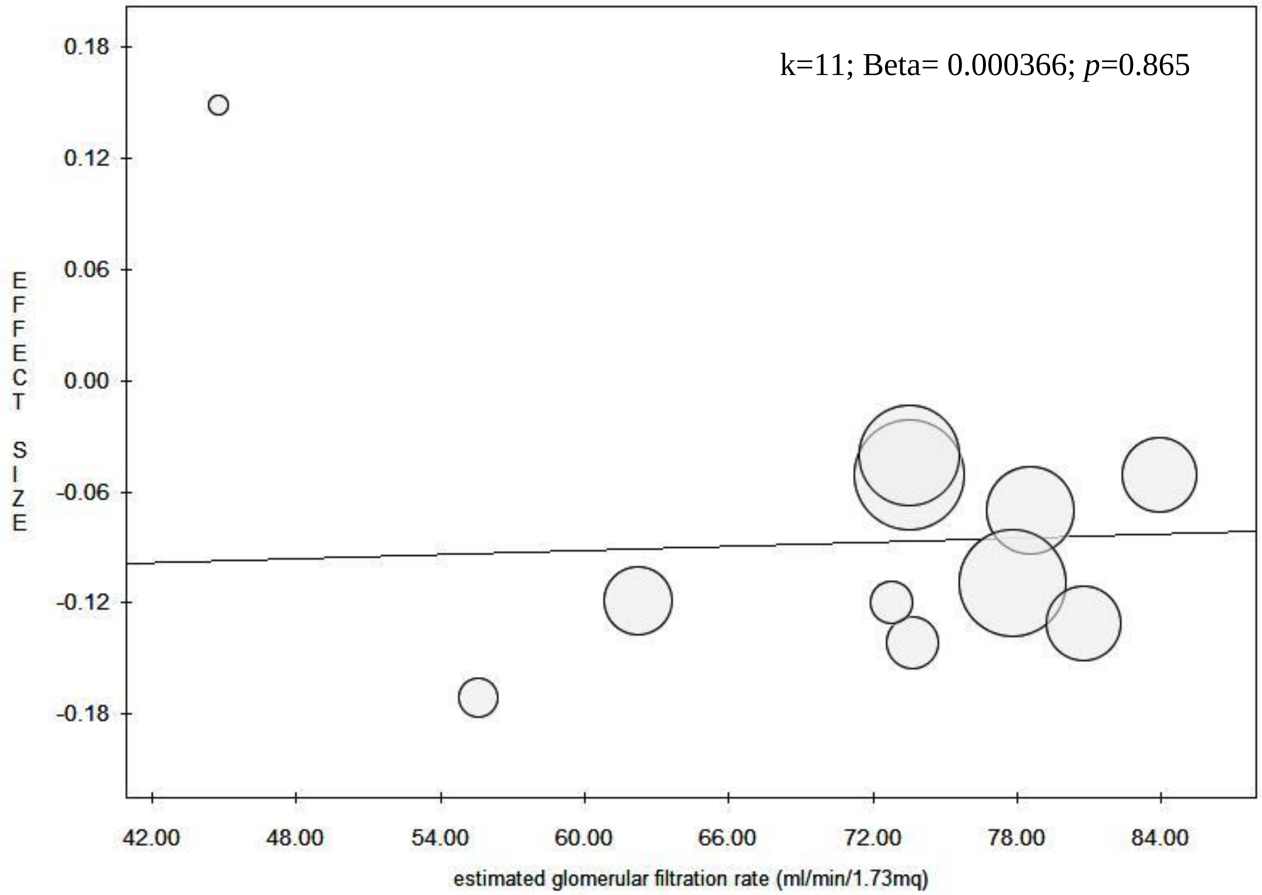

Supplemental Figure 2, Panel (F). Association between ES and prevalence of diabetics, regarding LDLc.

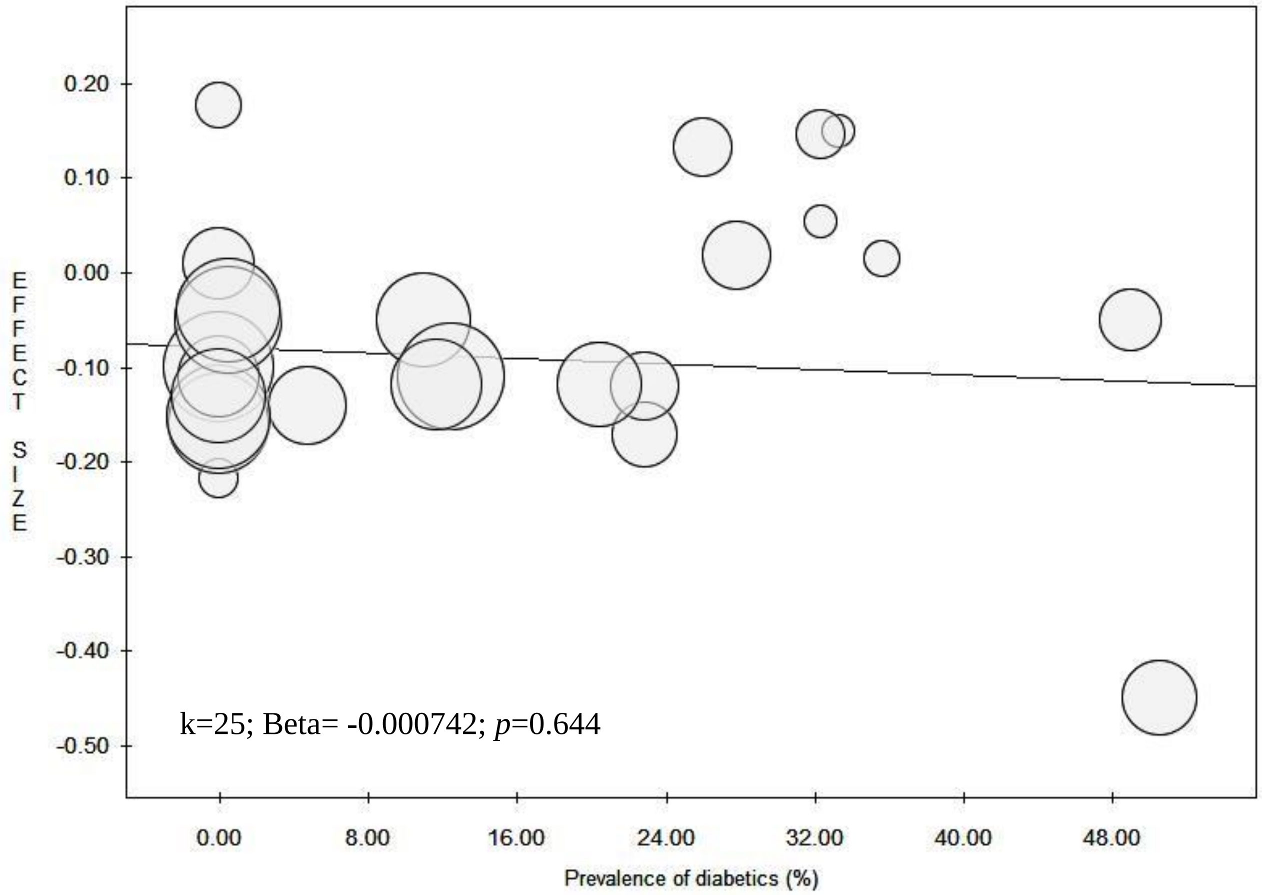

Supplemental Figure 2, Panel (G). Association between ES and prevalence of hypertensives, regarding LDLc.

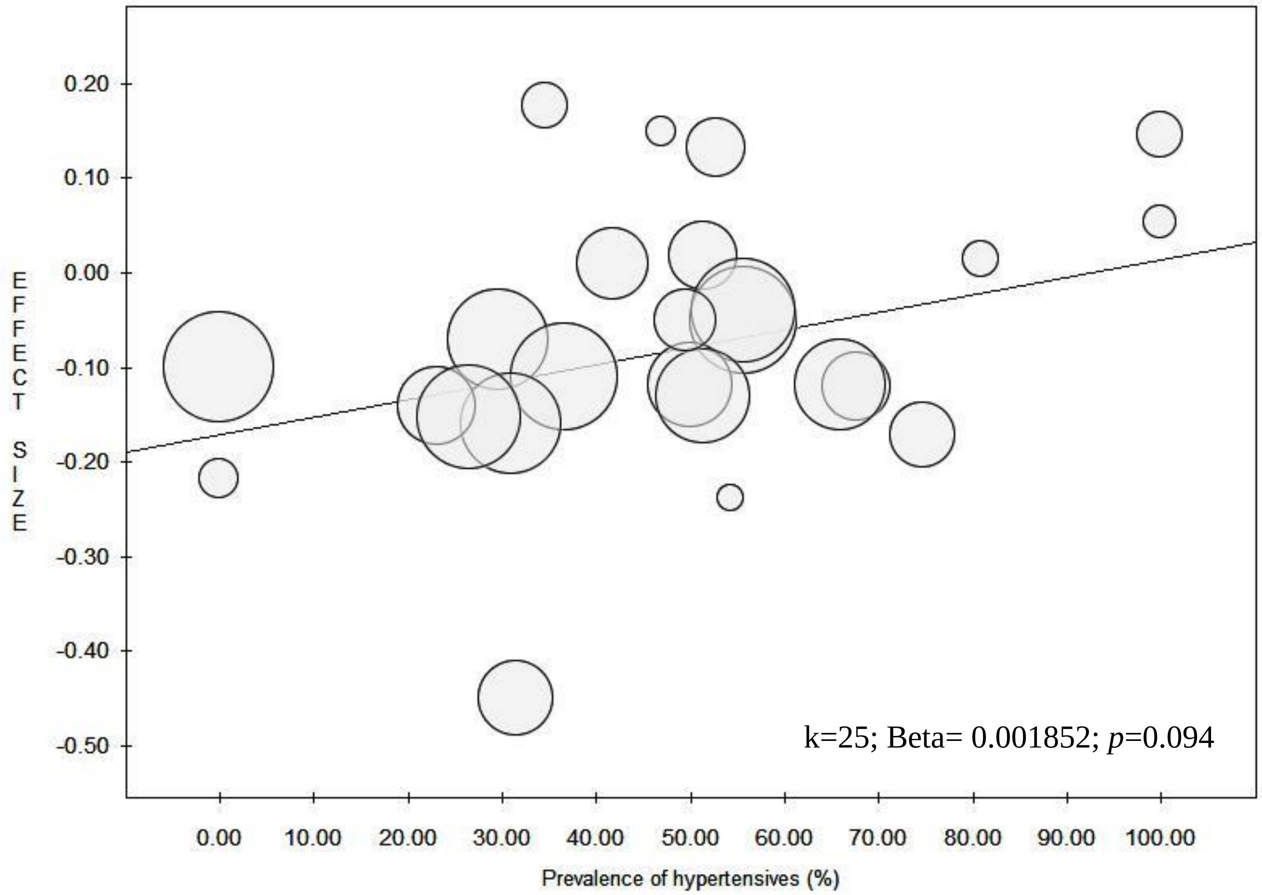

Supplemental Figure 2, Panel (H). Association between ES and prevalence of lipid-lowering therapy, regarding LDLc.

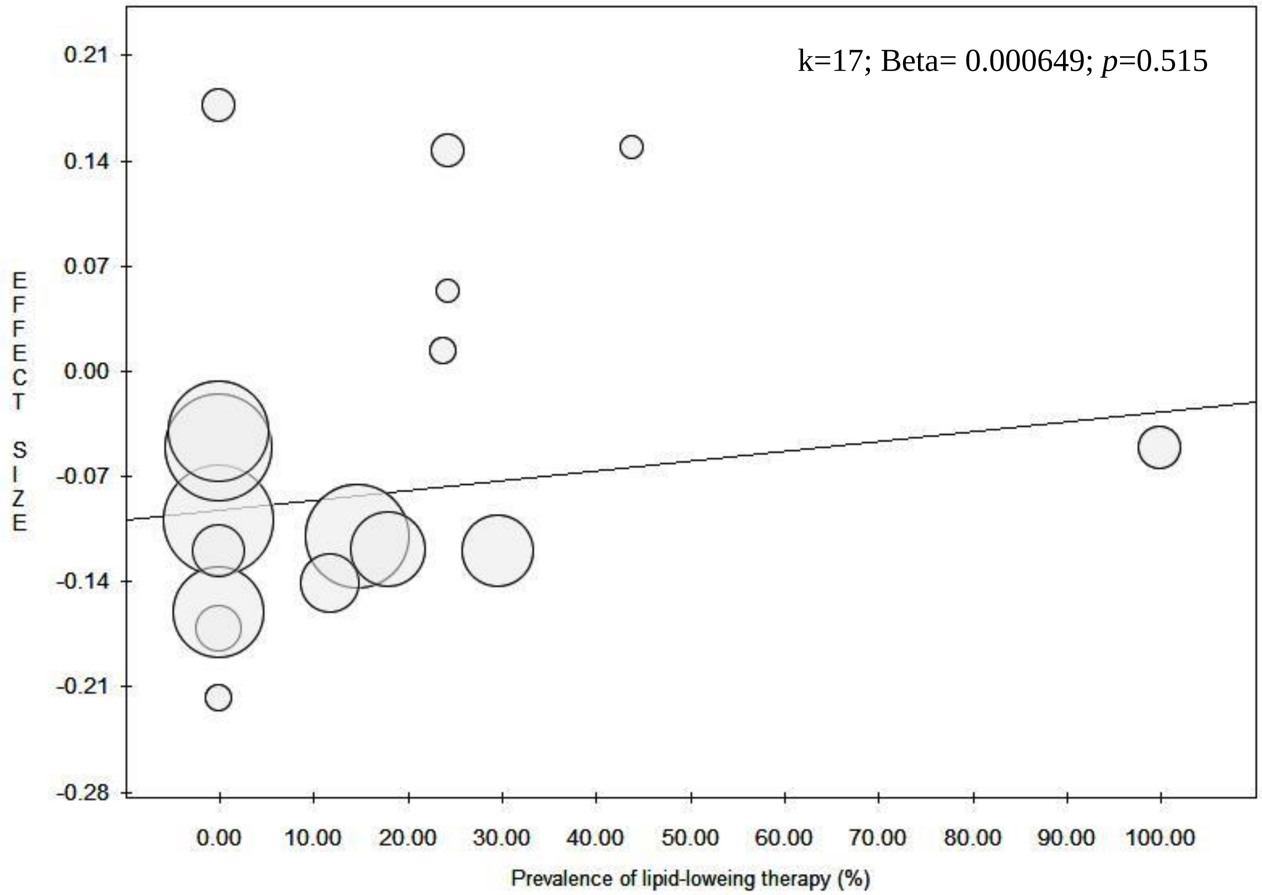

Supplemental Figure 3, Panel (A). Association between ES and age, regarding HDLc.

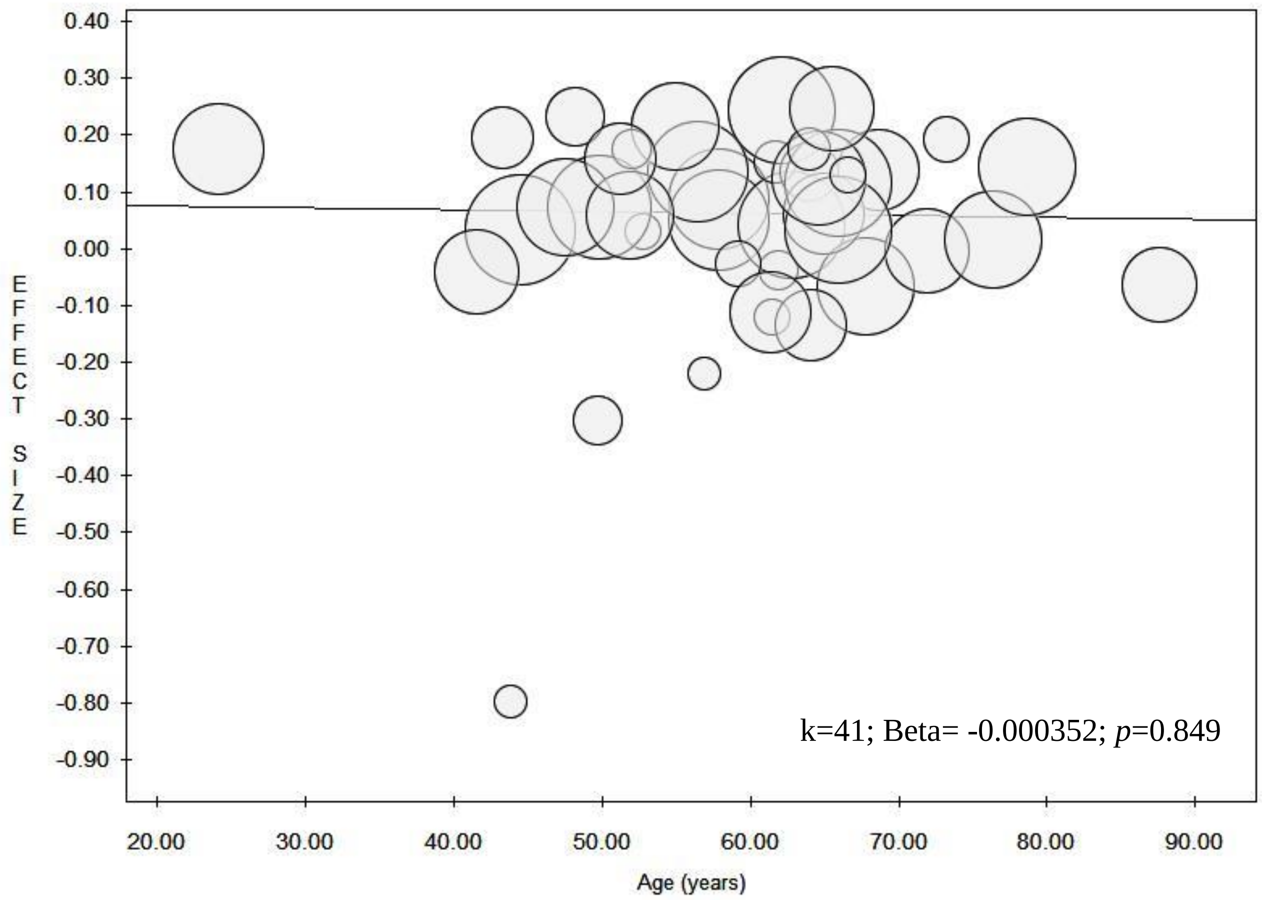

Supplemental Figure 3, Panel (B). Association between ES and body mass index, regarding HDLc.

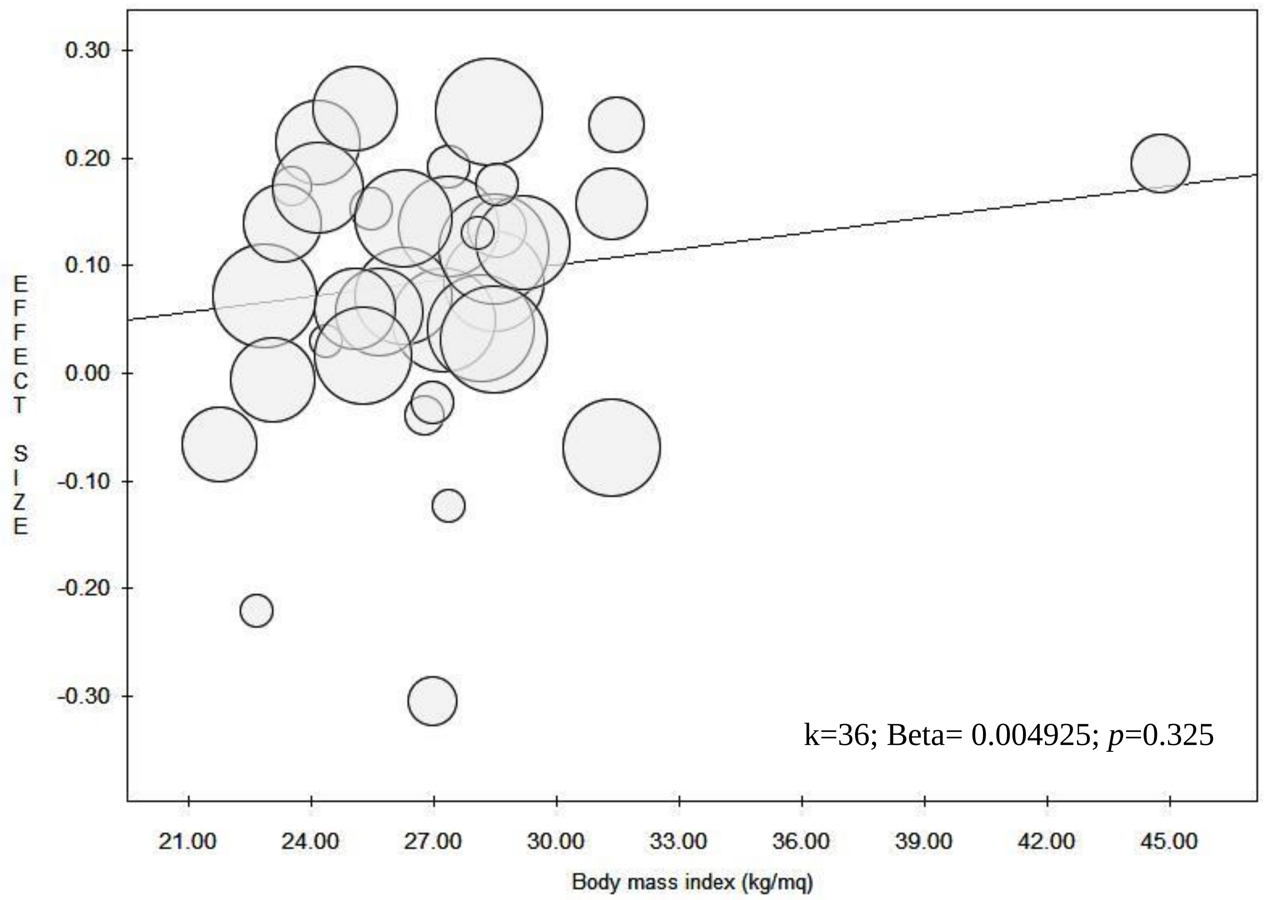

Supplemental Figure 3, Panel (C). Association between ES and NT-proBNP levels, regarding HDLc.

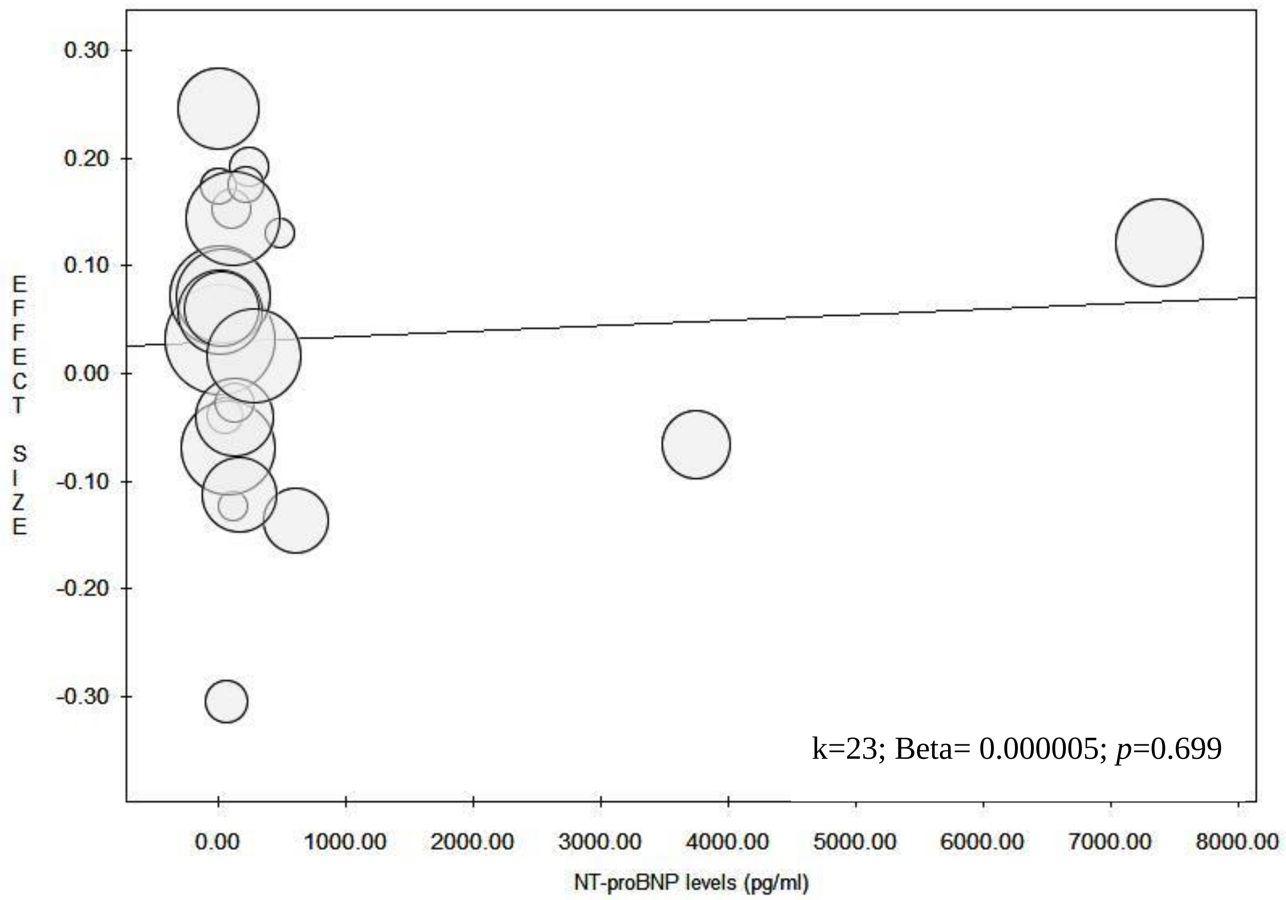

Supplemental Figure 3, Panel (D). Association between ES and prevalence of males, regarding HDLc.

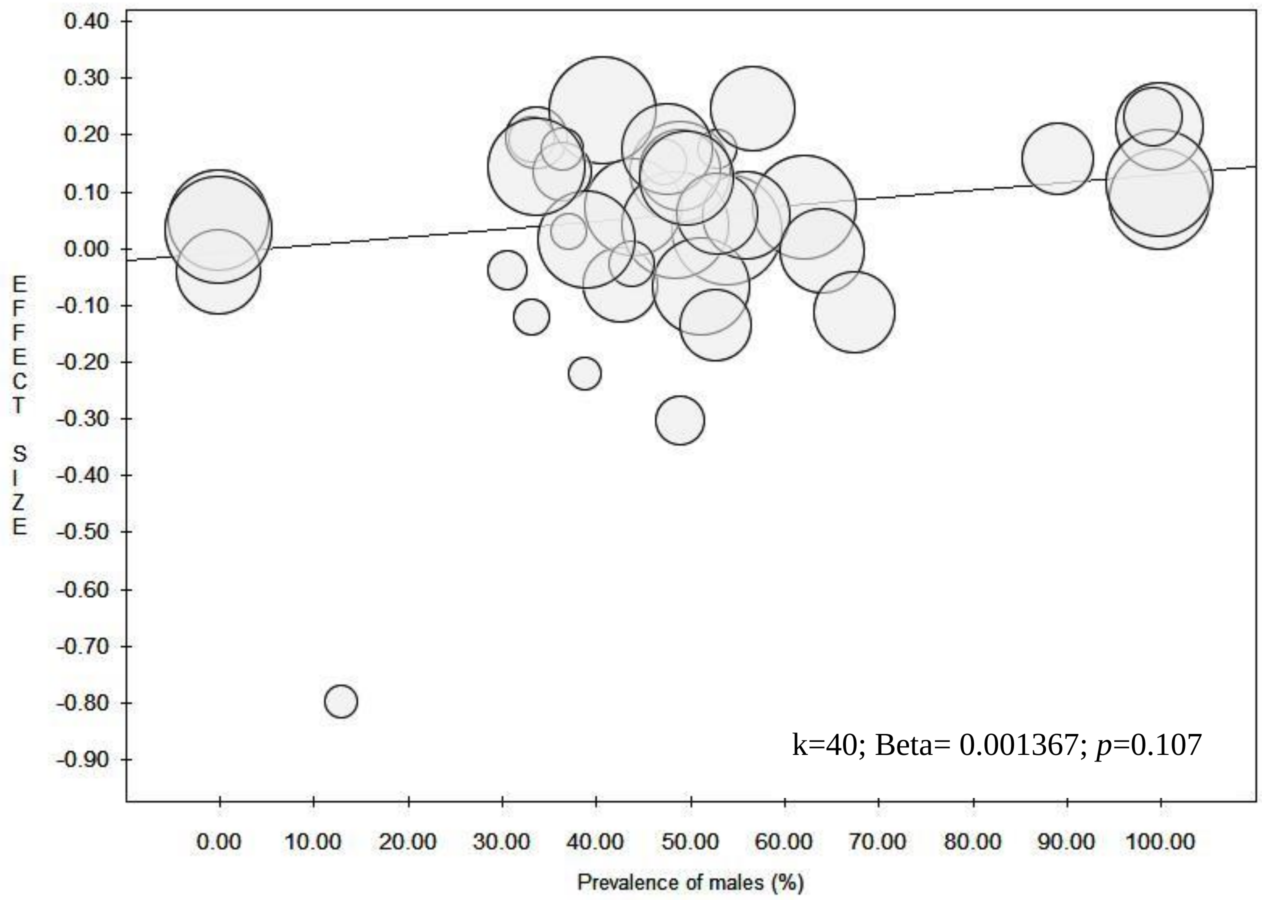

Supplemental Figure 3, Panel (E). Association between ES and estimated glomerular filtration rate, regarding HDLc.

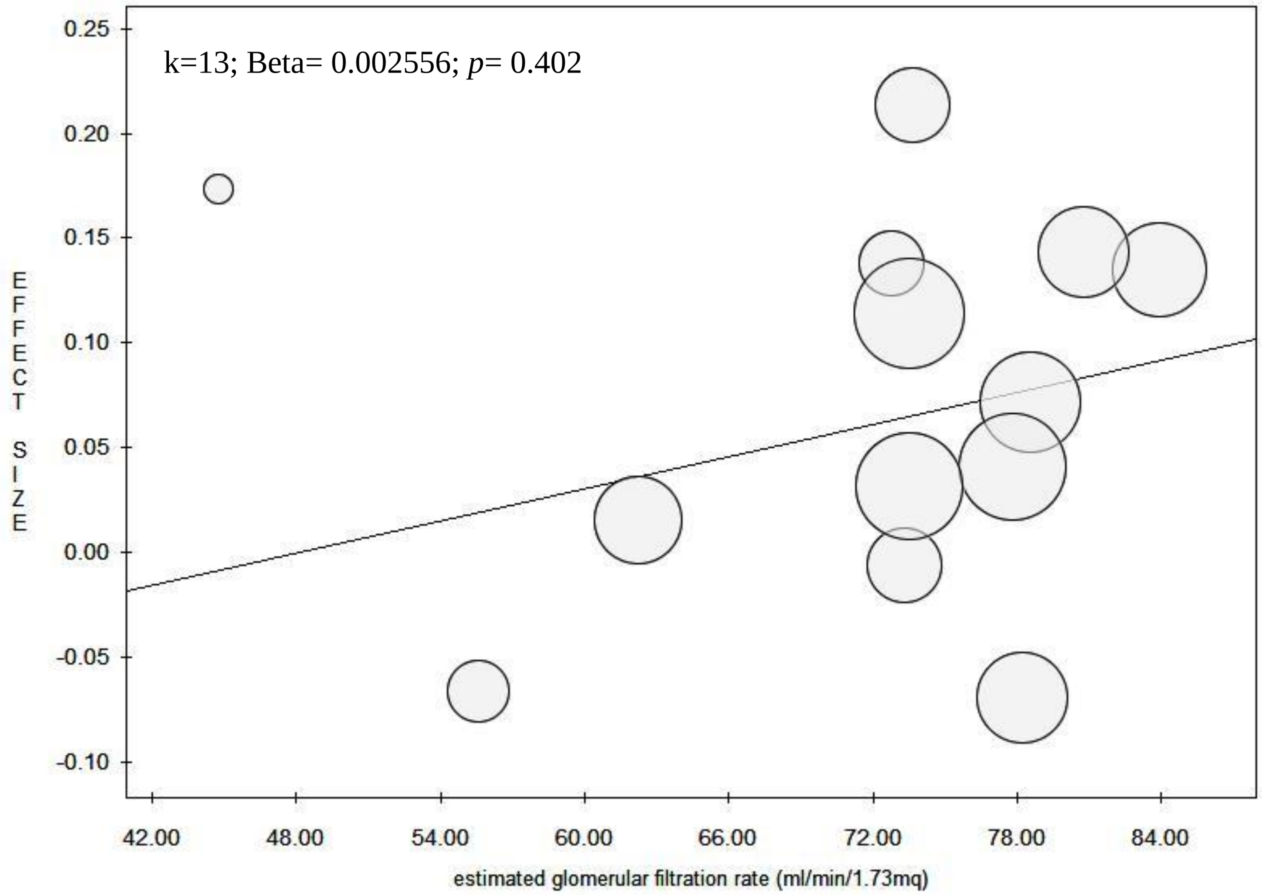

Supplemental Figure 3, Panel (F). Association between ES and prevalence of diabetics, regarding HDLc.

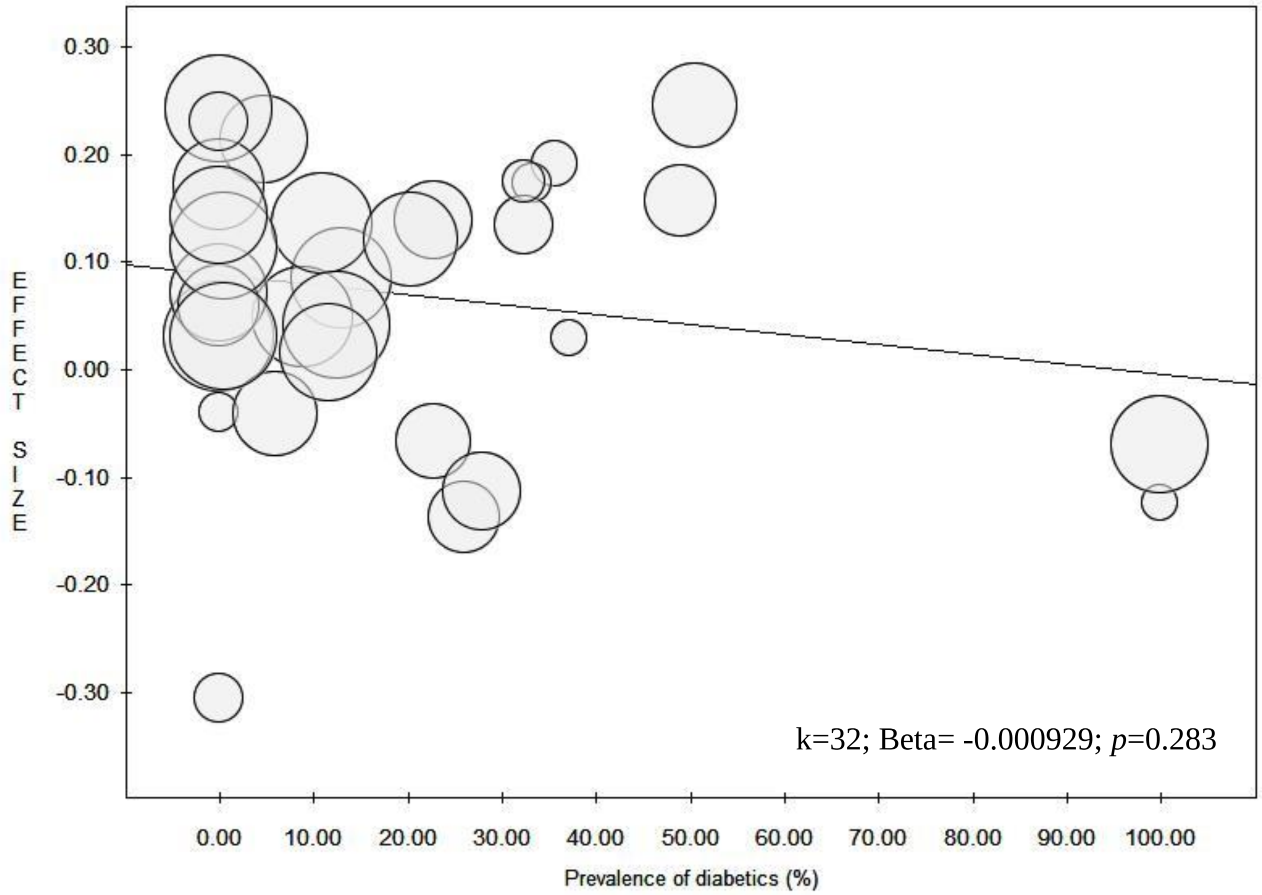

Supplemental Figure 3, Panel (G). Association between ES and prevalence of hypertensives, regarding HDLc.

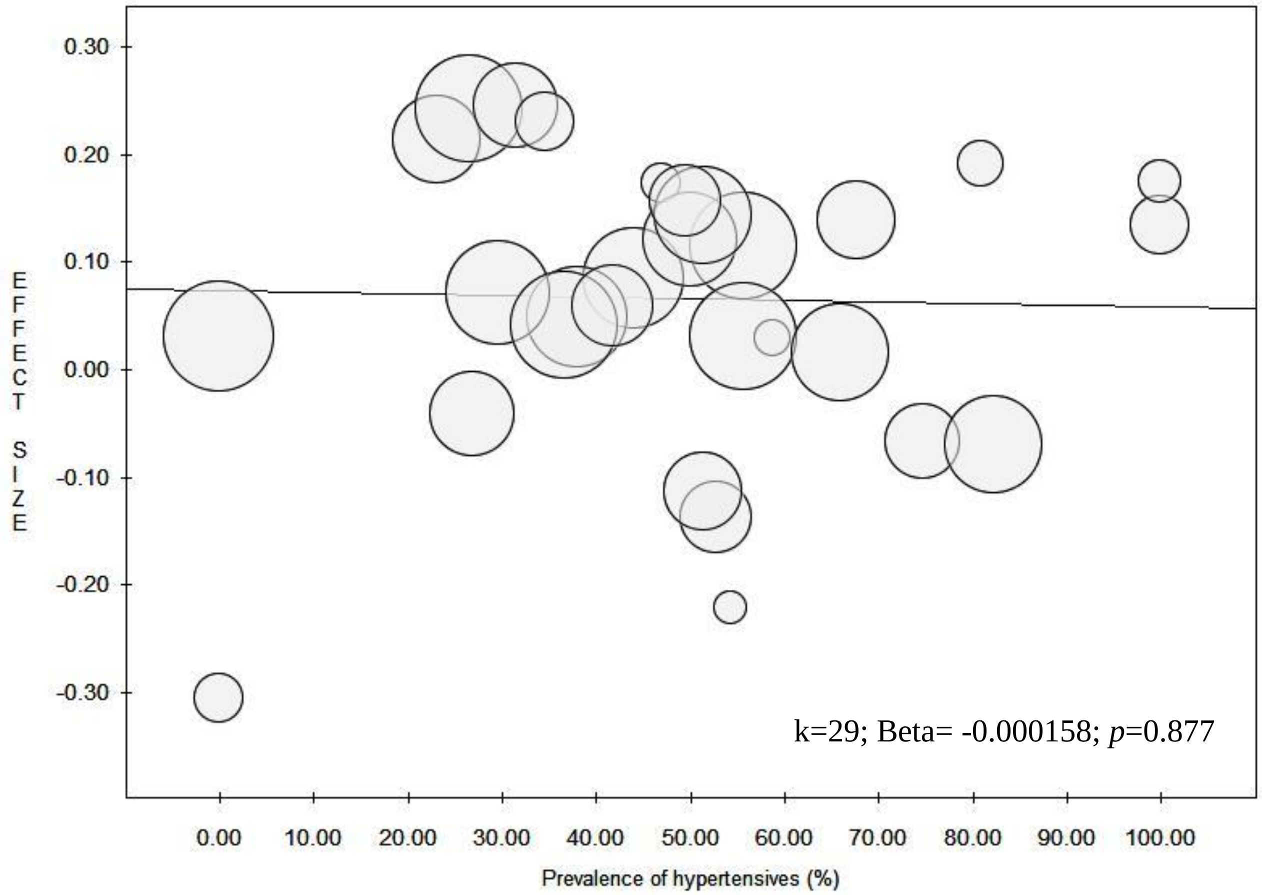

Supplemental Figure 3, Panel (H). Association between ES and prevalence of lipid-lowering therapy, regarding HDLc.

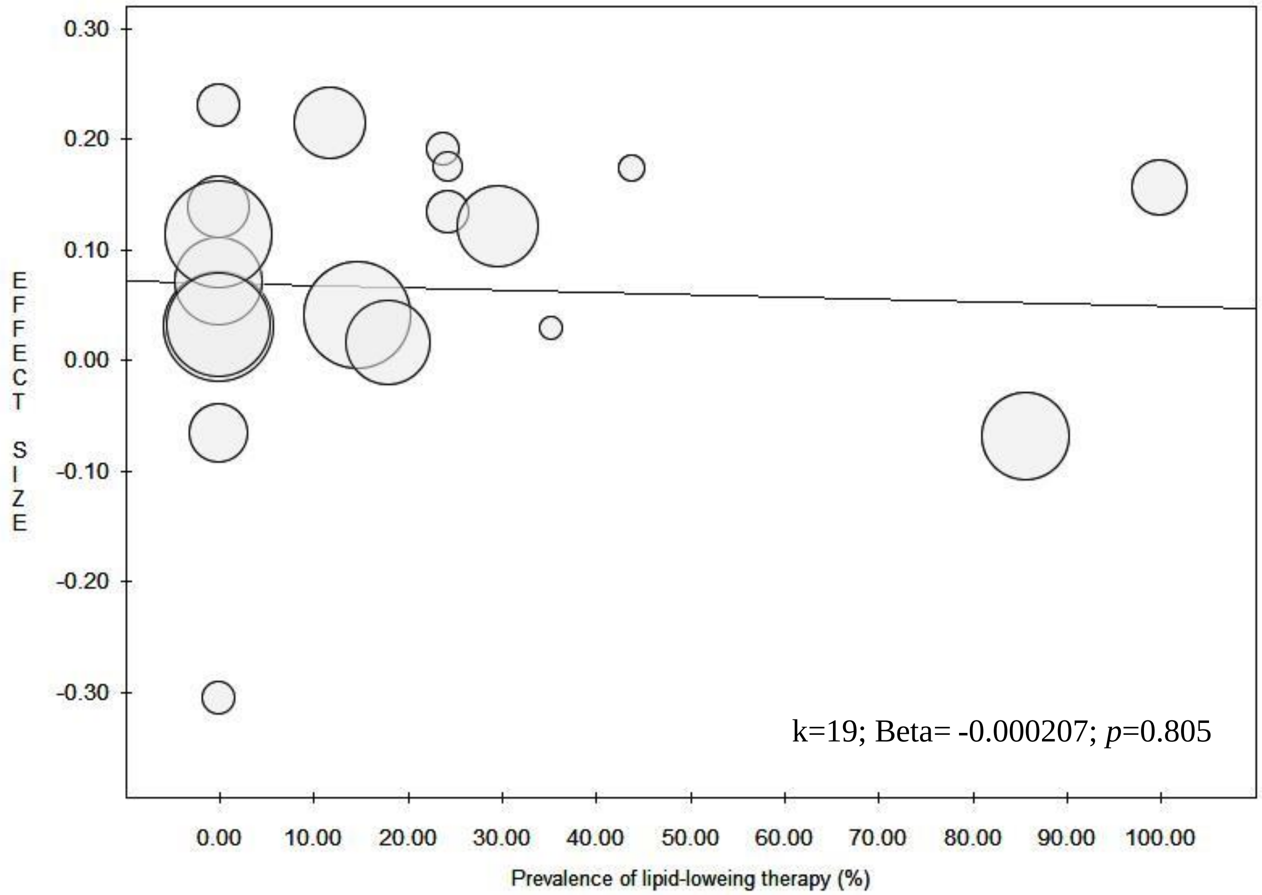

Supplemental Figure 4, Panel (A). Association between ES and age, regarding triglycerides.

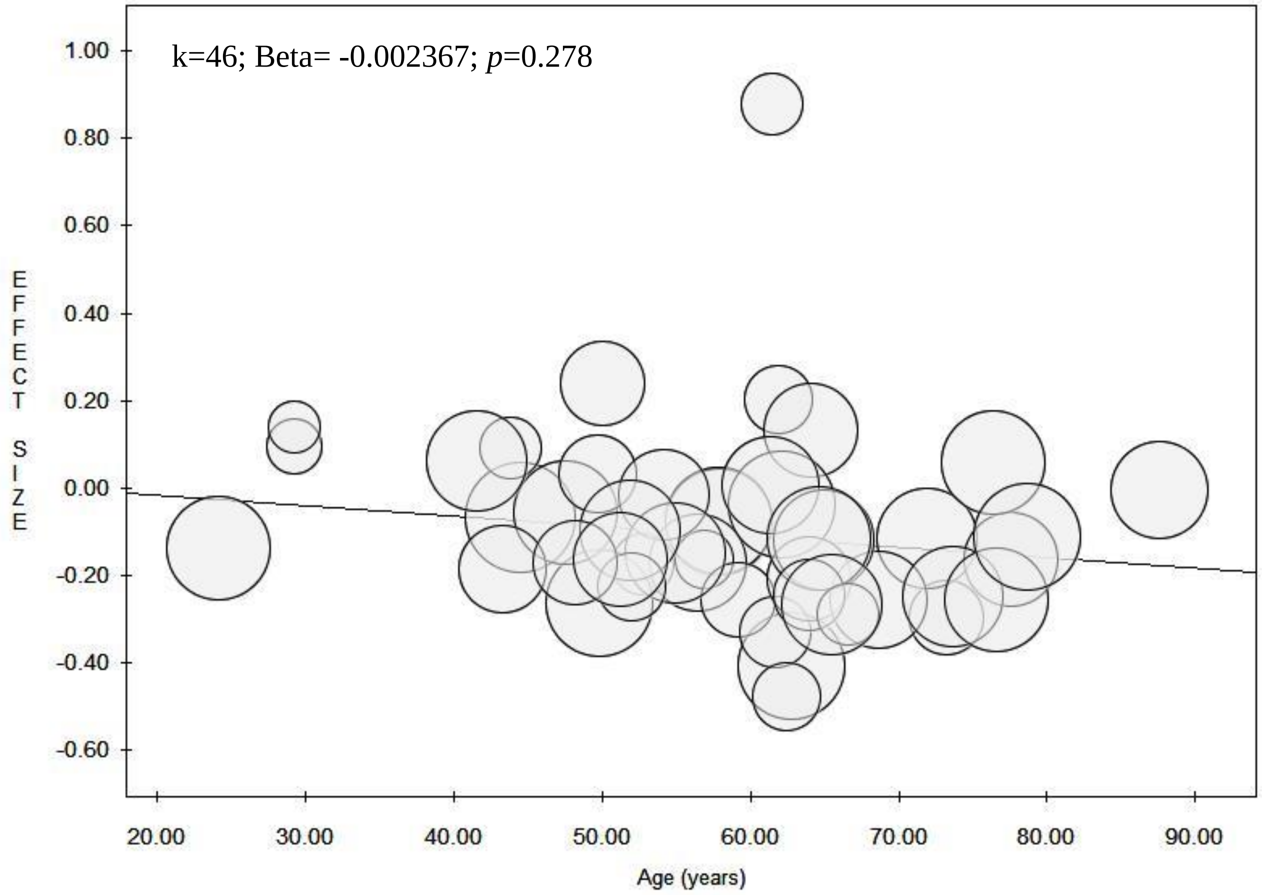

Supplemental Figure 4, Panel (B). Association between ES and body mass index, regarding triglycerides.

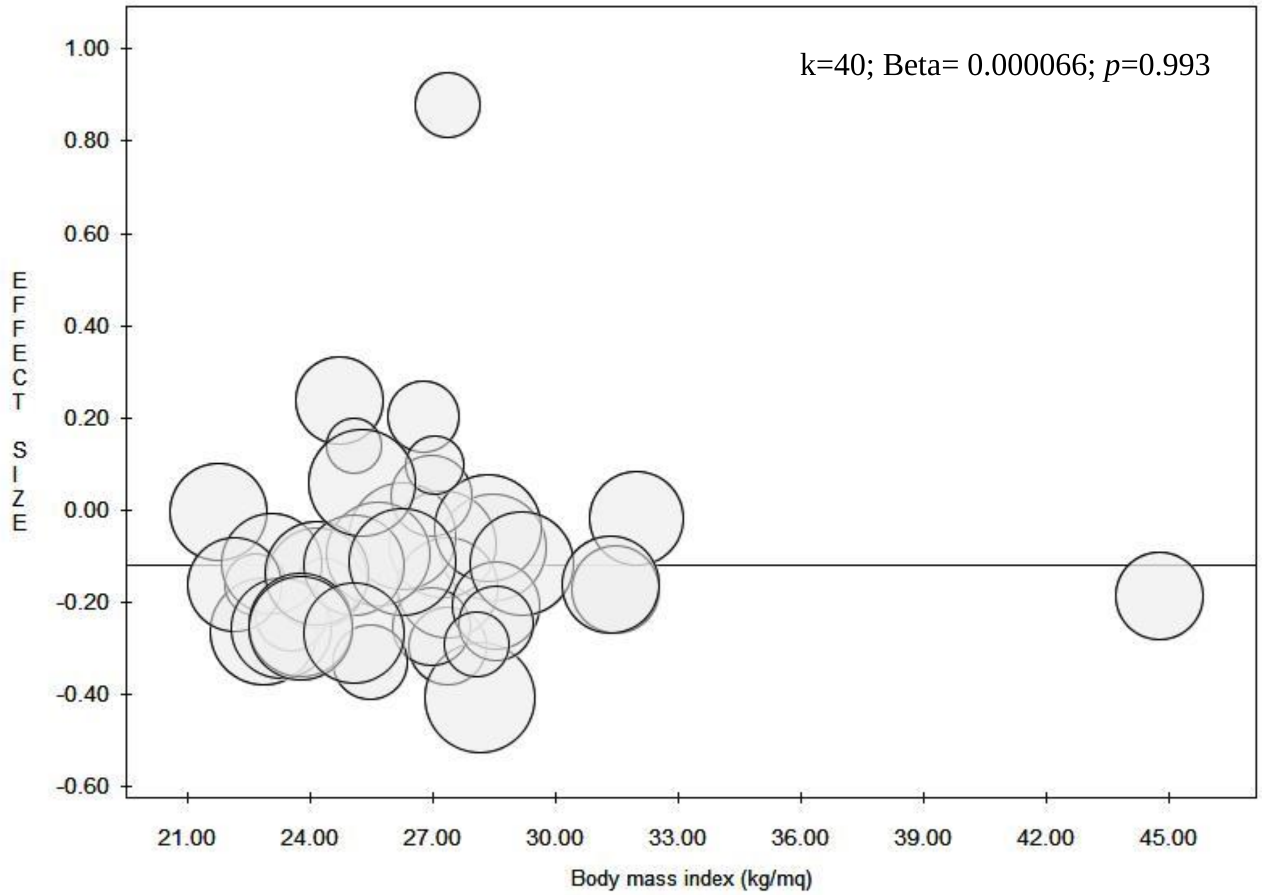

Supplemental Figure 4, Panel (C). Association between ES and NT-proBNP levels, regarding triglycerides.

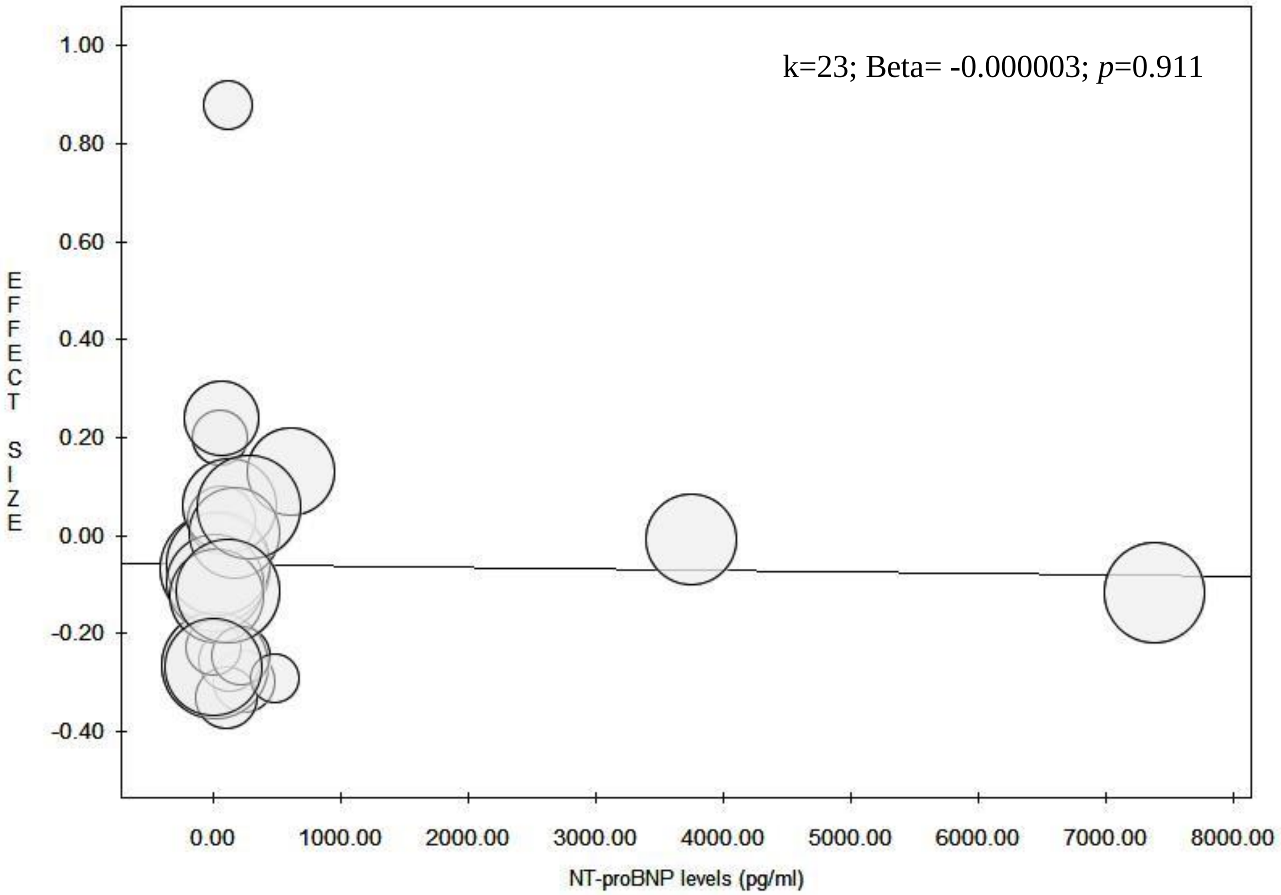

Supplemental Figure 4, Panel (D). Association between ES and prevalence of males, regarding triglycerides.

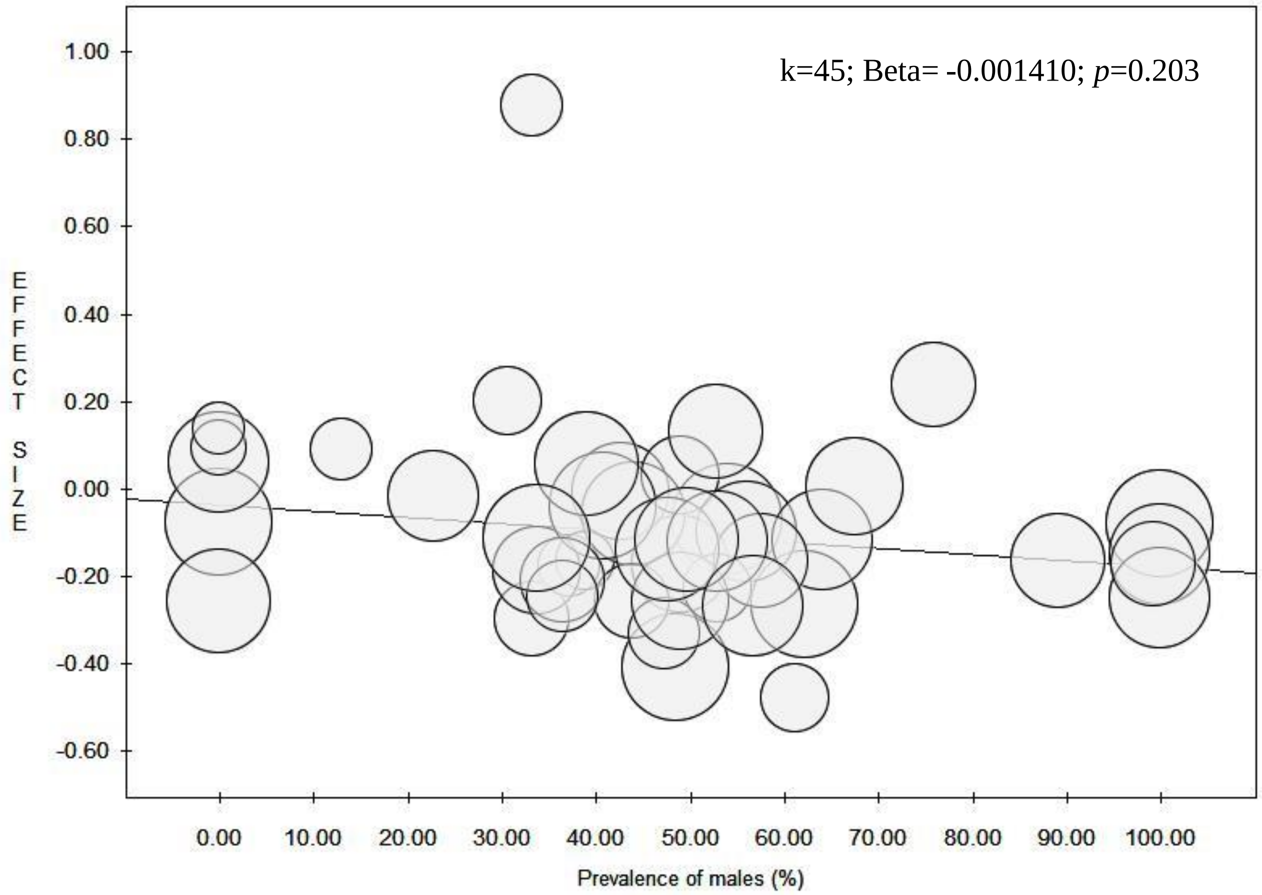

Supplemental Figure 4, Panel (E). Association between ES and estimated glomerular filtration rate, regarding triglycerides.

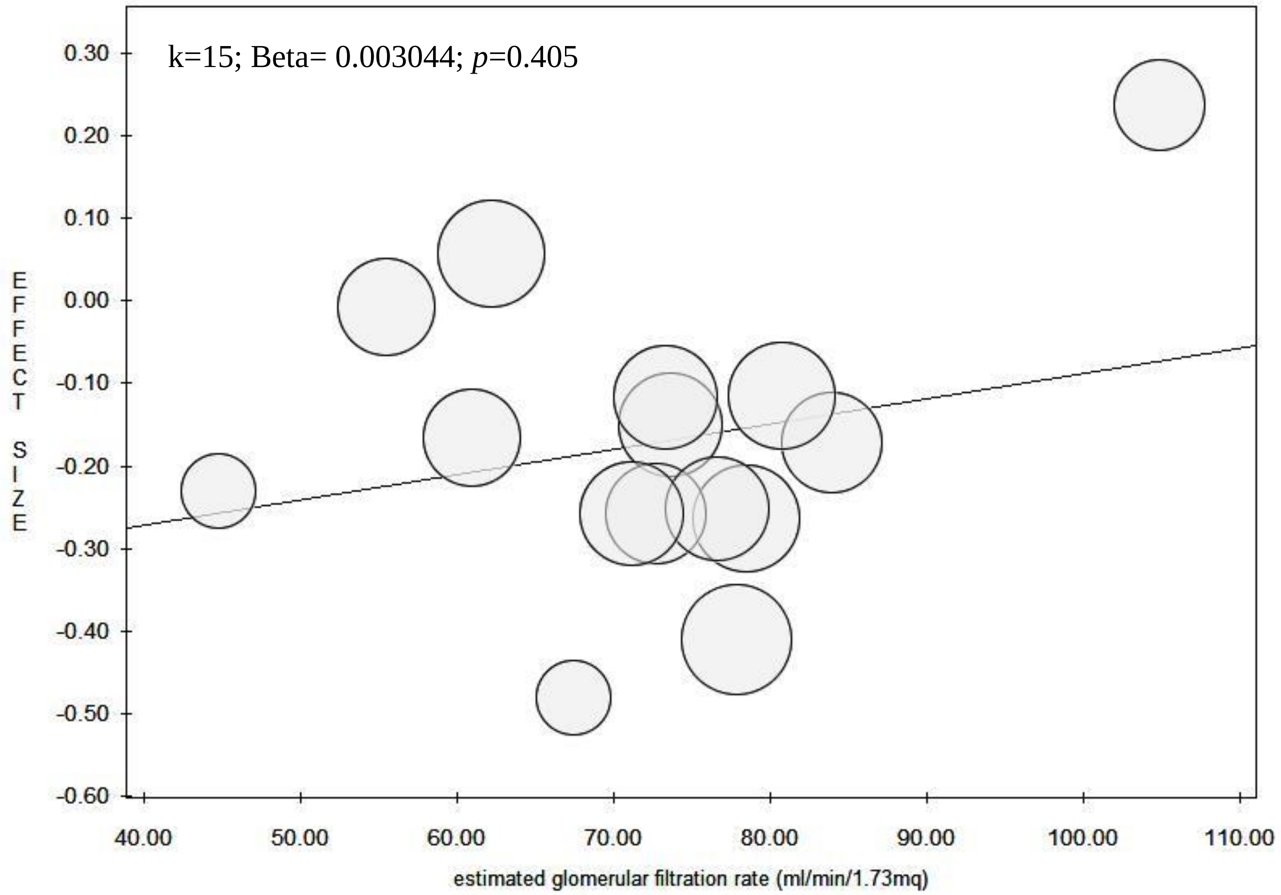

Supplemental Figure 4, Panel (F). Association between ES and prevalence of diabetics, regarding triglycerides.

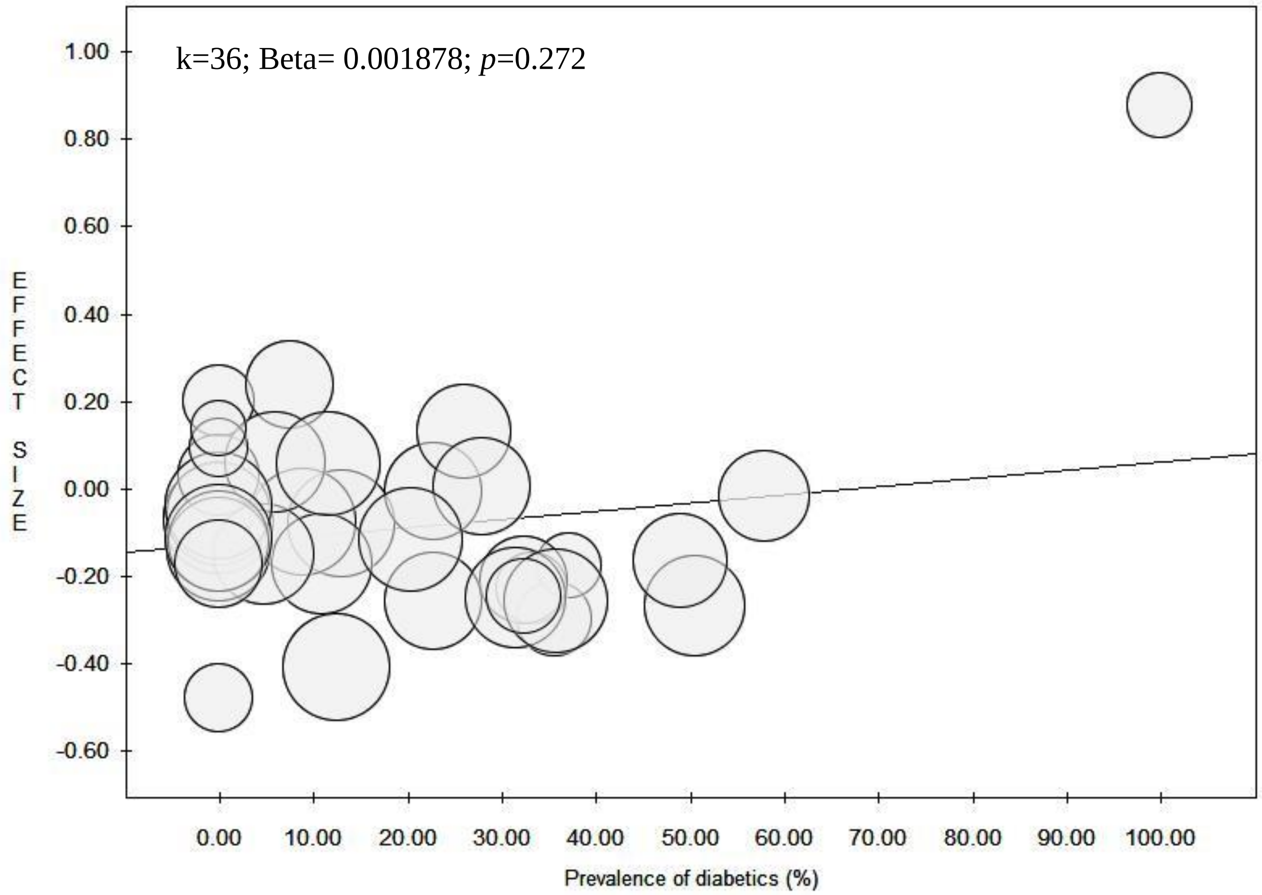

Supplemental Figure 4, Panel (G). Association between ES and prevalence of hypertensives, regarding triglycerides.

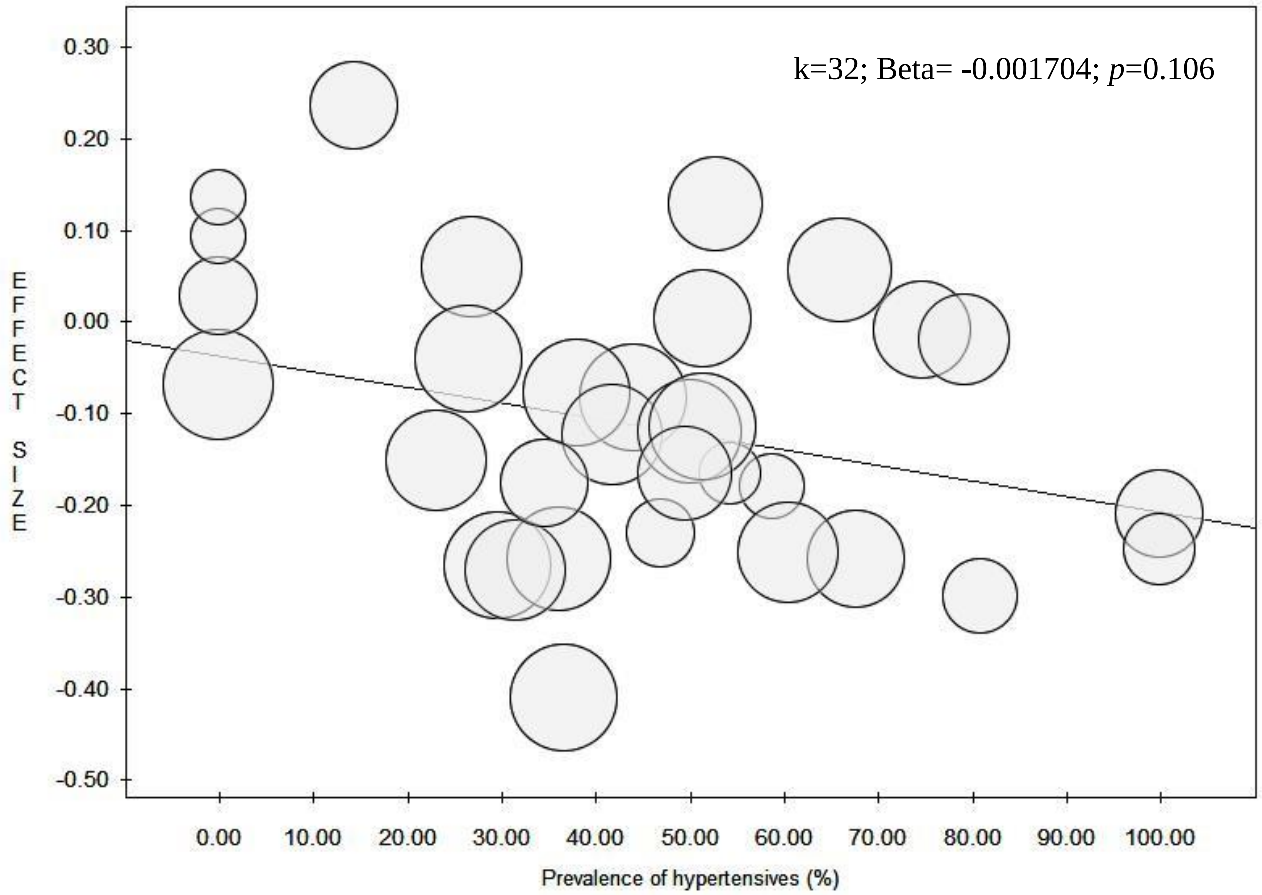

Supplemental Figure 4, Panel (H). Association between ES and prevalence of lipid-lowering therapy, regarding triglycerides.

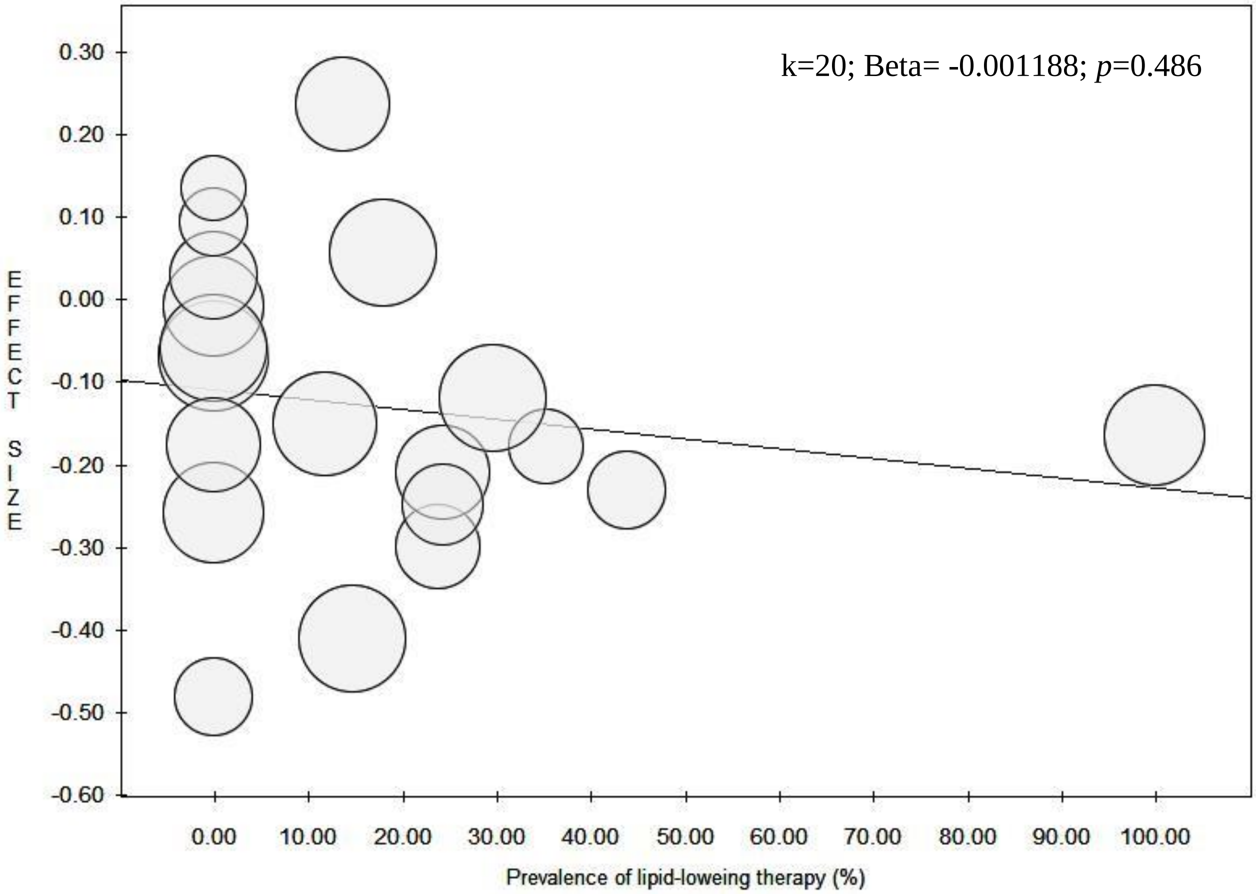

Supplemental Figure 5. Funnel plot and trim-and-fill analysis of studies that evaluated the association between cardiac NPs and total cholesterol (k = 32). Open circles indicate the analyzed studies, full circles indicate the trimmed studies.

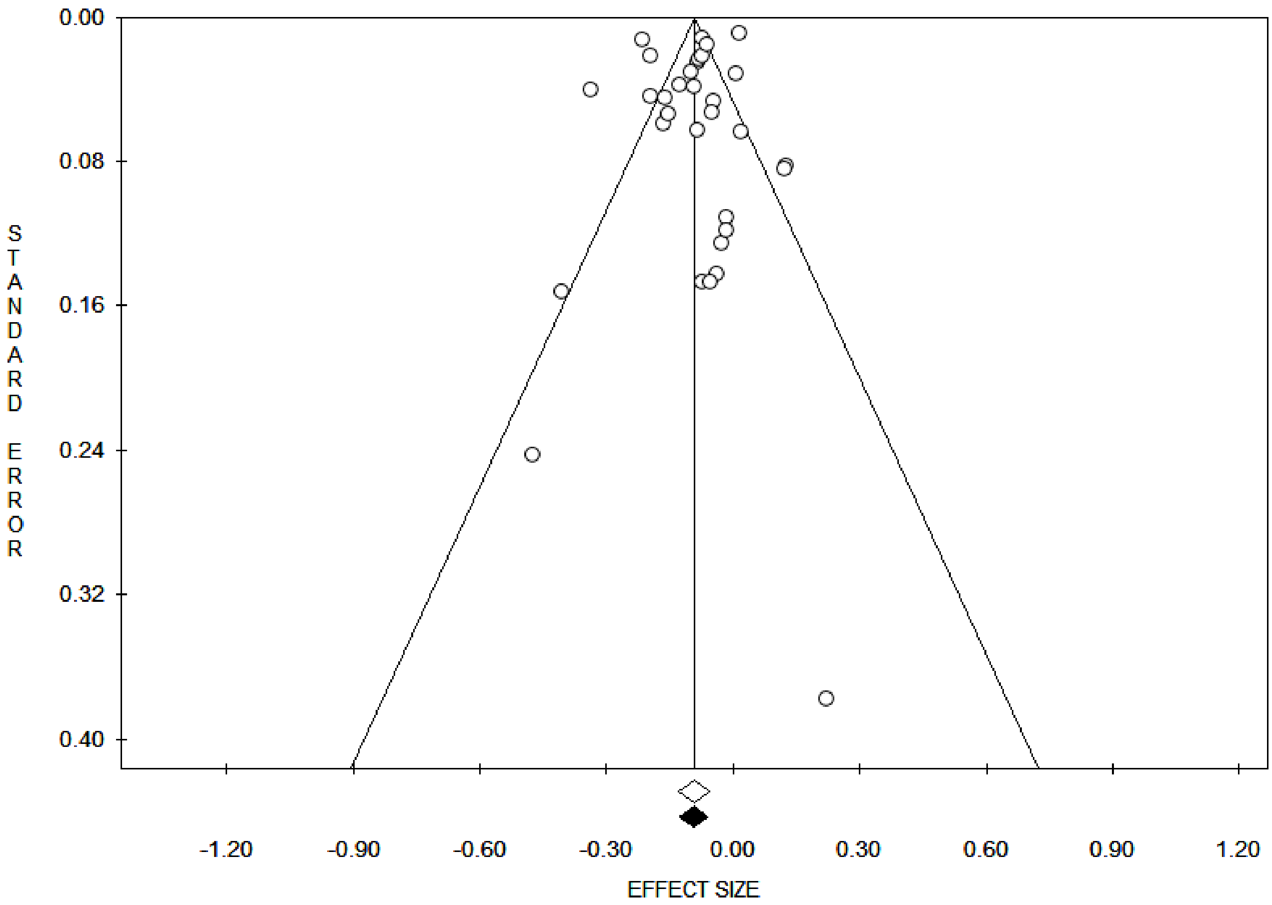

Supplemental Figure 6. Funnel plot and trim-and-fill analysis of studies that evaluated the association between cardiac NPs and LDLc (k = 31). Open circles indicate the analyzed studies, full circles indicate the trimmed studies.

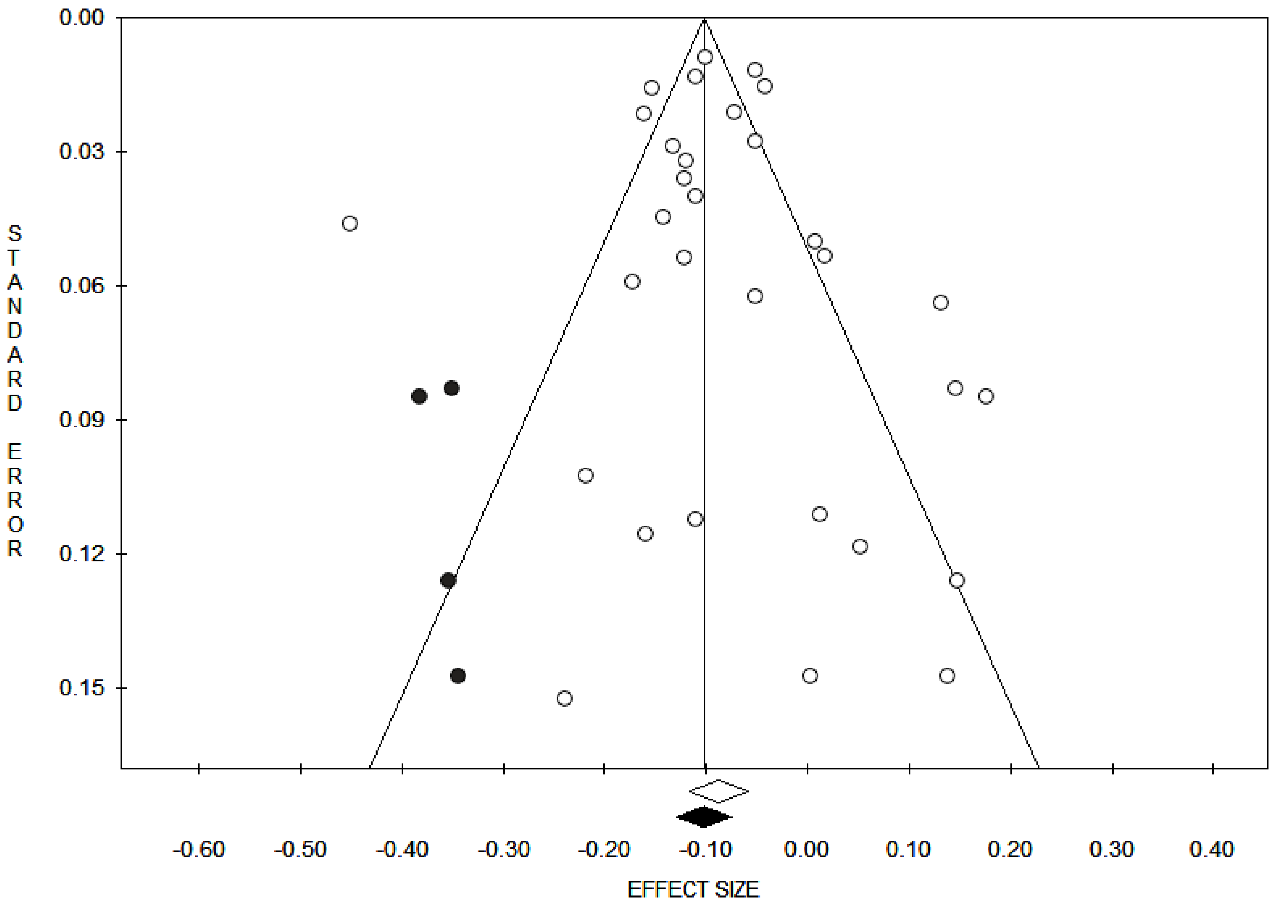

Supplemental Figure 7. Funnel plot and trim-and-fill analysis of studies that evaluated the association between cardiac NPs and HDLc (k = 41). Open circles indicate the analyzed studies, full circles indicate the trimmed studies.

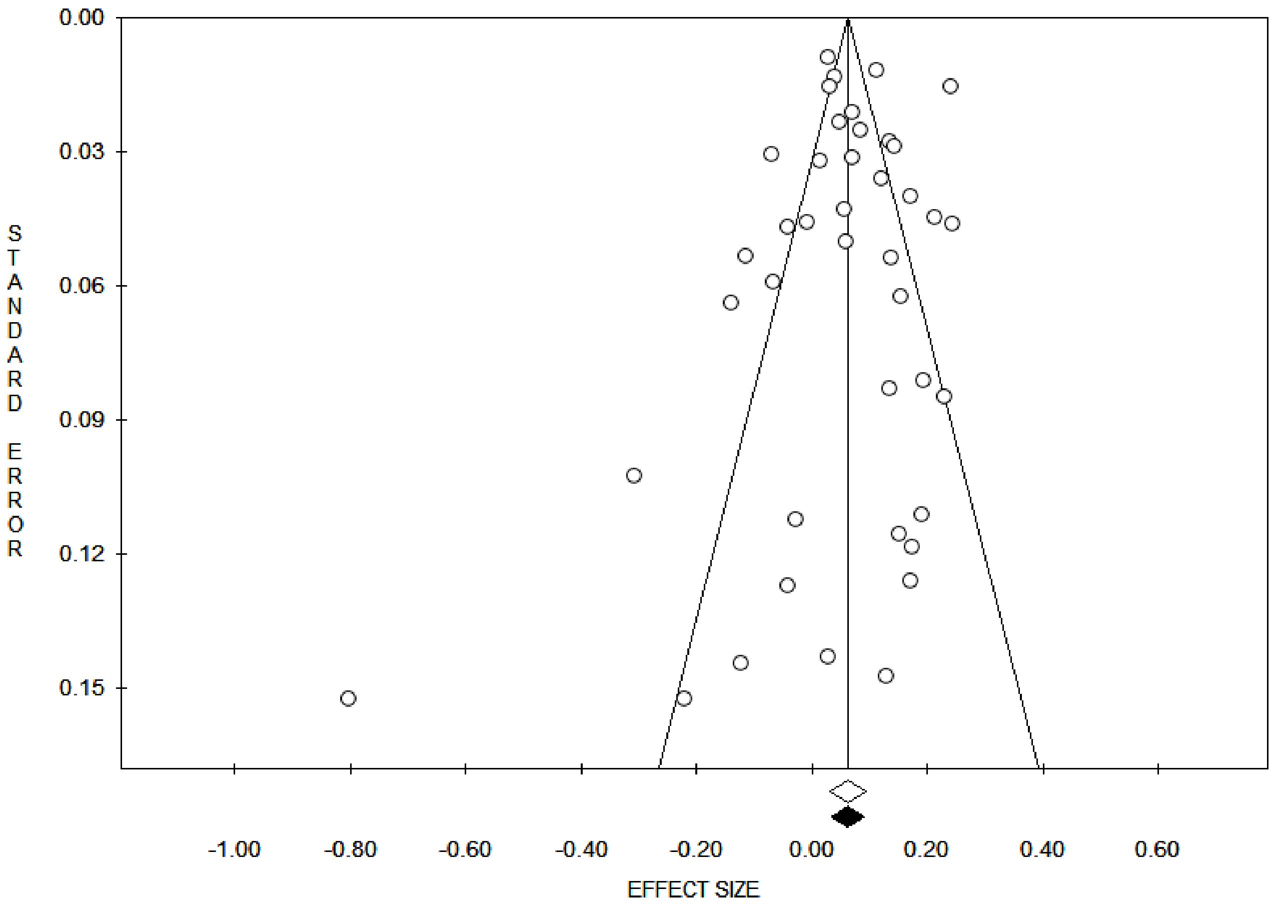

Supplemental Figure 8. Funnel plot and trim-and-fill analysis of studies that evaluated the association between cardiac NPs and triglycerides (k = 46). Open circles indicate the analyzed studies, full circles indicate the trimmed studies.

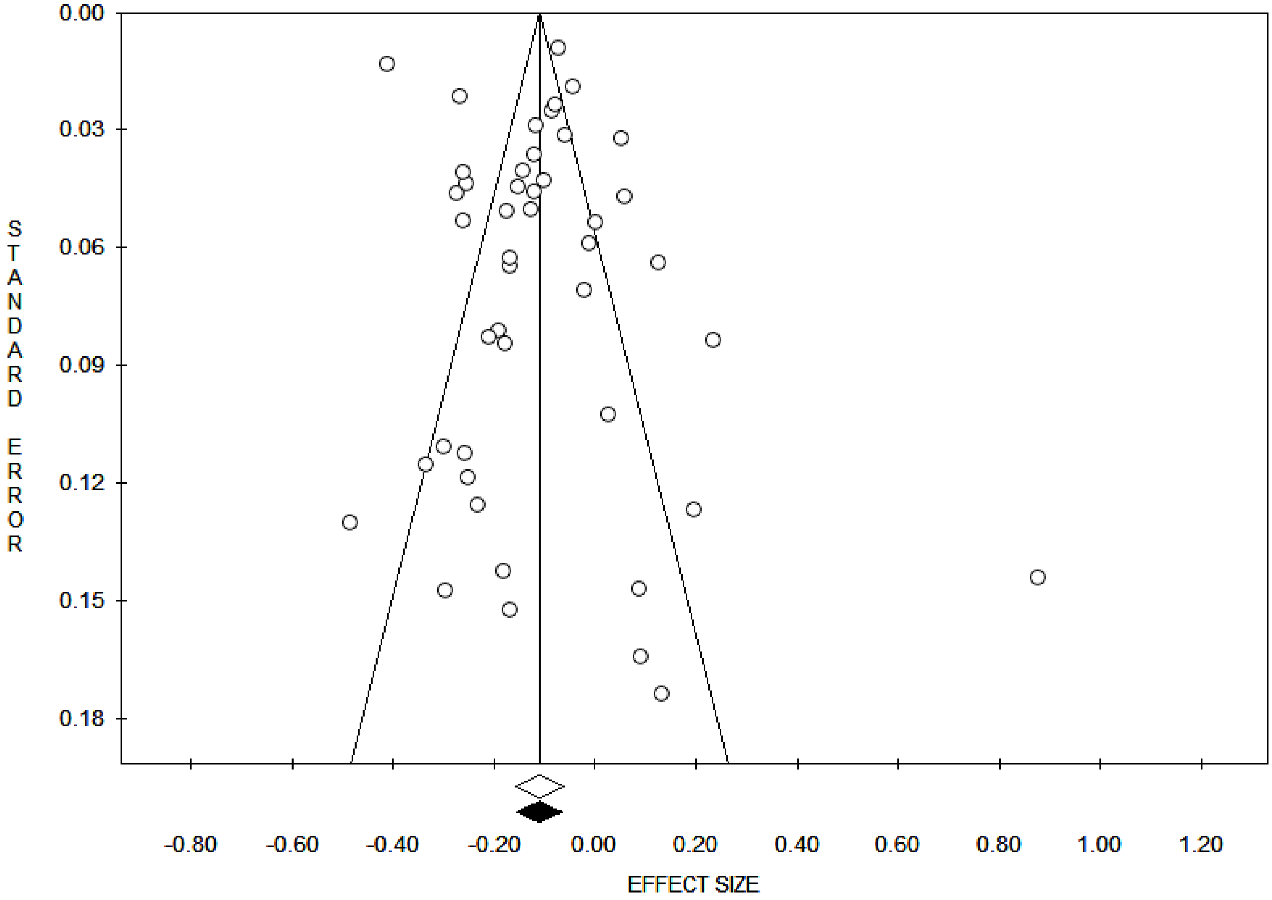

Supplement: Supplementary file 1 — Supplementary Information [file 41598_2019_55680_MOESM1_ESM.pdf]
